# Diphosphino‐Functionalized 1,8‐Naphthyridines: a Multifaceted Ligand Platform for Boranes and Diboranes

**DOI:** 10.1002/chem.202102721

**Published:** 2021-10-11

**Authors:** Jingjing Cui, Maximilian Dietz, Marcel Härterich, Felipe Fantuzzi, Wei Lu, Rian D. Dewhurst, Holger Braunschweig

**Affiliations:** ^1^ School of Chemistry and Environmental Engineering Wuhan Institute of Technology Wuhan 430205 P. R. China; ^2^ Institute for Inorganic Chemistry Julius-Maximilians-Universität Würzburg Am Hubland 97074 Würzburg Germany; ^3^ Institute for Sustainable Chemistry & Catalysis with Boron Julius-Maximilians-Universität Würzburg Am Hubland 97074 Würzburg Germany; ^4^ Institute for Physical and Theoretical Chemistry Julius-Maximilians-Universität Würzburg Emil-Fischer-Str. 42 97074 Würzburg Germany

**Keywords:** pincer ligand, naphthyridine, boron, diborane, potassium reagent

## Abstract

A 1,8‐naphthyridine diphosphine (NDP) reacts with boron‐containing Lewis acids to generate complexes featuring a number of different naphthyridine bonding modes. When exposed to diborane B_2_Br_4_, NDP underwent self‐deprotonation to afford [NDP‐B_2_Br_3_]Br, an unsymmetrical diborane comprised of four fused rings. The reaction of two equivalents of monoborane BBr_3_ and NDP in a non‐polar solvent provided the simple phosphine‐borane adduct [NDP(BBr_3_)_2_], which then underwent intramolecular halide abstraction to furnish the salt [NDP‐BBr_2_][BBr_4_], featuring a different coordination mode from that of [NDP‐B_2_Br_3_]Br. Direct deprotonation of NDP by KHMDS or PhCH_2_K generates mono‐ and dipotassium reagents, respectively. The monopotassium reagent reacts with one or half an equivalent of B_2_(NMe_2_)_2_Cl_2_ to afford NDP‐based diboranes with three or four amino substituents.

## Graphical abstract

Five diphosphino‐functionalized 1,8‐naphthyridine (NDP)‐based boron compounds were synthesized via two different methods: direct coordination and salt elimination. In these NDP‐based boron compounds, NDP behaves as a flexible, multifaceted ligand. For example, it functions as a tridentate ligand toward B_2_Br_4_ and as a bidentate ligand for BBr_3_. Furthermore, NDP binds through only one nitrogen to a diaminodiborane to furnish tri‐ and tetraamino diboranes.

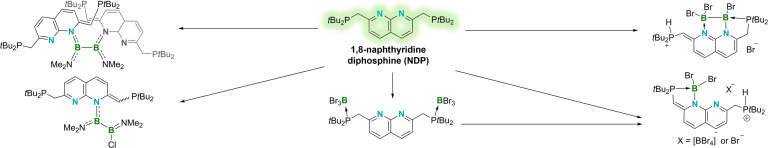

## Introduction

Pincer ligands are versatile scaffolds for transition metal (TM) complex design and have been the focus of organometallic chemistry for almost half a century^[1]^ due to their useful physical and chemical properties, such as geometrical robustness, high thermal stability, facile modification, and in some cases non‐innocent ligand behavior. In contrast, although the application of pincer ligands in main‐group element chemistry has also been investigated,^[2]^ progress in this area remains relatively limited. Reports of pincer complexes of boron are even more limited and, at present, most of the research concerning pincer‐ligand‐based boron compounds has focused on the geometrical perturbation of the boron center[^2e^, ^2g^, ^3^] by pincer ligands and their consequential reactivity.

**Figure 1:**
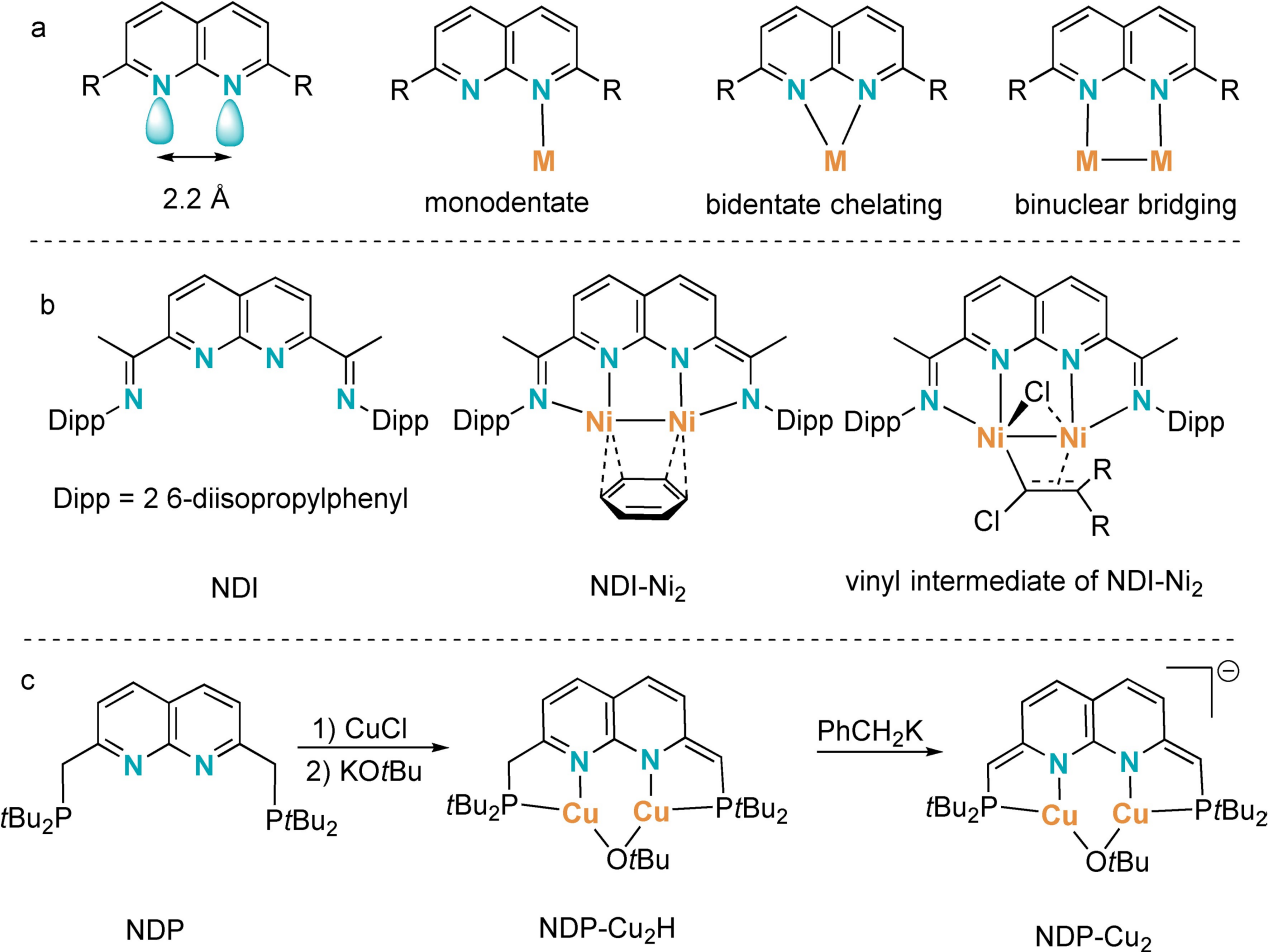
Structure, coordination, and TM complexes of 1,8‐naphthyridines.

When designing pincer ligands, choosing the right core structure for the intended purpose is important as it sets the basic steric and electronic properties of the coordination sites. From this perspective, 1,8‐naphthyridines (napy) are of particular interest due to the diverse coordination patterns (Figure 1a) reported for their TM complexes,^[4]^ and group 1,^[5]^ 2,^[6]^ 13,^[7]^ and 14^[8]^ main‐group‐element‐centered Lewis acids. A particularly interesting possibility of napy derivatives is the potential application of their complexes with boron in the field of fluorescence,[^7b^, ^7c^, ^7e^] two‐photon absorption,^[7j]^ photoluminescence,^[7d]^ and sensing materials.[^7f^, ^7g^] The close proximity of the two N atoms of the napy unit (ca. 2.2 Å)[^6^, ^9^] has also been found promising for metal‐metal cooperation (MMC) effects^[10]^ as demonstrated by Uyeda and coworkers[^5c^, ^10p^] using the diimine‐functionalized napy compound NDI (Figure 1b, left). Furthermore, an [Ni_2_(C_6_H_6_)] adduct of the same ligand (Figure 1b, middle) is an efficient catalyst for reactions such as vinylidene transfer.[^10e^, ^11^] Another interesting characteristic of napy‐based ligands is their propensity to undergo aromatization‐dearomatization processes, as observed by Broere and coworkers.^[12]^ Stepwise deprotonation of a copper(I) complex of NDP generates the partially and fully dearomatized species NDP−Cu_2_H and NDP−Cu_2_, respectively (Figure 1c), while the reverse transformation was realized by stepwise protonation. Complex NDP‐Cu_2_ activates H_2_ with one hydrogen adding to the Cu(I)Cu(I) unit and the other to the vinyl carbon atom, a confirmed example of metal‐ligand cooperation (MLC).^[13]^

Inspired by these works, we became interested in the interactions of NDP (compound **1**) with boranes and diboranes, with the hope of observing new coordination modes or reactions involving boron‐ligand cooperation. Herein we report a variety of products of combining boron‐containing species with ligand **1**, their unusual binding modes, as well as a number of problems we have encountered during the journey.

## Results and Discussion

**Scheme 1:**
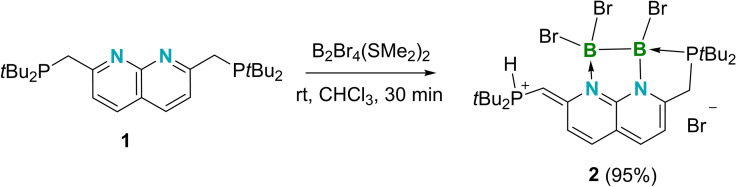
The synthesis of **2**.

Solid **1** and one molar equivalent of diborane B_2_Br_4_(SMe_2_)_2_ were mixed in CHCl_3_, leading to a clear yellow solution. The byproduct SMe_2_ and solvent were then removed, providing a fine, bright‐yellow powder (**2**, Scheme 1) that dissolves poorly in all common deuterated solvents. In order to fully characterize this compound, we prepared the compound via an NMR‐scale reaction in CDCl_3_ and recorded NMR spectra in situ. In the ^1^H NMR spectrum of **2**, a signal at 9.08 ppm (dd, ^1^ *J*_P‐H_=474.4 Hz, ^3^ *J*_H‐H_=13.3 Hz) indicated the presence of a P‐H bond adjacent to a CH unit. A ^1^H NMR signal at 6.18 ppm (dd, ^3^ *J*_H‐H_=13.3 Hz, ^2^ *J*_P‐H_=6.9 Hz) and a ^31^P NMR signal at 19.8 ppm (d, ^1^ *J*_P‐H_=474.4 Hz) reflected the C=CH−PH connectivity of the compound. The chemical shift at 2.09 ppm indicated the liberation of SMe_2_. In the ^31^P{^1^H} NMR spectrum, the signal found at 56.0 ppm is significantly broader (Figure S3) than that at 19.8 ppm, due to the coordination of the former to boron. A single broad resonance at 0.3 ppm was detected in the ^11^B{^1^H} NMR spectrum, likely due to the superposition of two ^11^B signals due to their identical coordination numbers.

**Figure 2:**
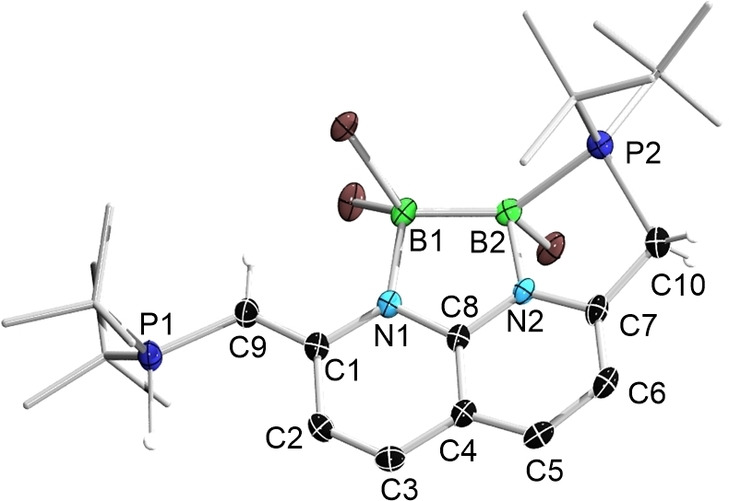
Solid‐state structure of the cation of **2** (hydrogen atoms, except for those on P1, C9, and C10, and ellipsoids of the *t*Bu groups, are omitted for clarity). Thermal ellipsoids are set at the 50 % probability level.

A single‐crystal X‐ray diffraction study revealed that **2** features four fused rings as shown in Figure 2. The dearomatized naphthyridine unit remains nearly flat with a sum of bond angles of 719.92° and 719.76° for the N1‐ and N2‐containing six‐membered rings, respectively. With respect to the plane defined by N1, C8, and N2, the largest deviation for atoms of the naphthyridine unit is that of C6, an atom‐to‐plane distance of 0.20 Å. The two boron atoms lie on opposite sides of the aforementioned plane to give an approximately flat five‐membered ring (sum of bond angles 538.12°). The B−B distance is 1.745(5) Å, suggesting the presence of a B−B single bond enforced by the NDP scaffold (typical B−B single bond distance: ca. 1.72 Å^[14]^ and the bond length is affected by the distance between the coordination sites of the ligands^[15]^). The B1‐N1 (1.582(4) Å) and B2‐N2 (1.548(4) Å) distances are very similar to each other and match those of other boron‐bound napy compounds (1.56–1.60 Å),[^7a^, ^7b^, ^7c^, ^7d^, ^7e^, ^7h^] confirming their single‐bond character.

**Figure 3:**
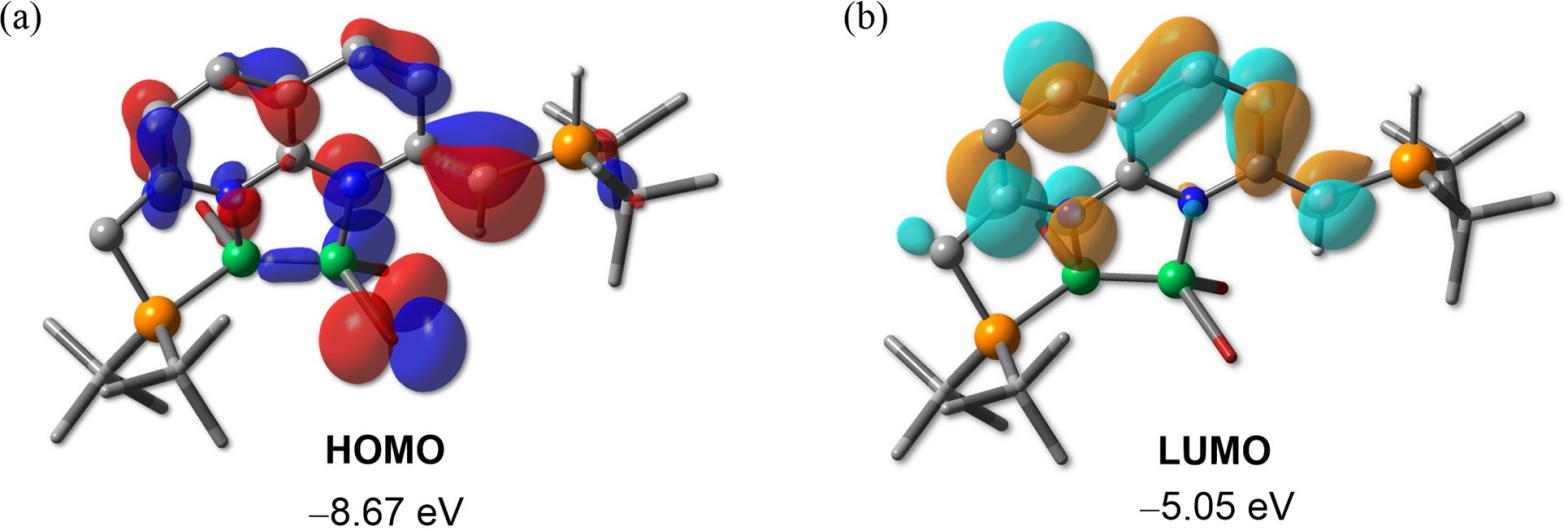
Frontier molecular orbitals of the cation of **2**. Plots of the HOMO (a) and LUMO (b) calculated at the PBE0‐D3(BJ)/6‐31+G**/LanL2DZ(Br) level. Isovalue=0.04. Hydrogen atoms are omitted for clarity.

For a better understanding of the bonding situation and electron distribution of the cation of **2**, we carried out theoretical calculations at the PBE0‐D3(BJ)/6‐31+G**/LanL2DZ(Br) level of theory. As depicted by the frontier canonical Kohn‐Sham molecular orbitals (Figure 3), both the highest occupied molecular orbital (HOMO) and lowest unoccupied molecular orbital (LUMO) reside mainly on the napy unit and the exocyclic C=C bonds, with the B−B bond only slightly contributing to the HOMO. To the best of our knowledge, compound **2** represents the first example of a dinuclear main‐group‐element complex of a napy species with a bridging coordination mode.

**Scheme 2:**
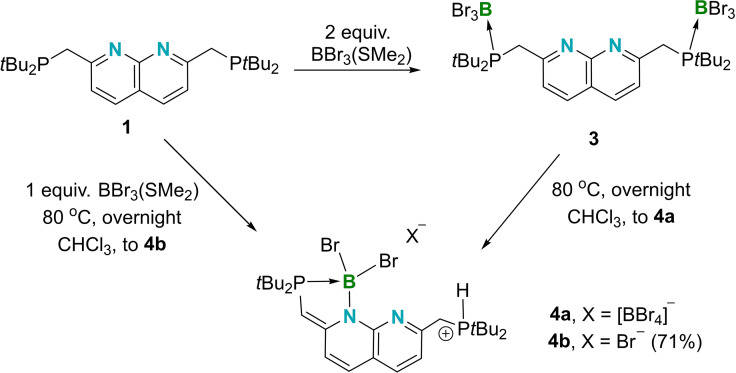
The synthesis of **3**, its isomerization to **4** **a**, and the generation of **4** **b**.

When two equivalents of monoborane BBr_3_⋅SMe_2_ were mixed with **1** in hexane, a light yellow precipitate was obtained, the NMR spectroscopic data for which suggested the generation of a symmetric phosphine‐borane adduct, compound **3** (Scheme 2). The ^1^H NMR spectroscopic resonances of the new species **3** were found to lower field than those of 1, with the signals corresponding to the naphthyridine and PCH_2_ units found at 8.19, 7.80, and 4.28 ppm in CDCl_3_, respectively (compared to 7.99, 7.67, and 3.31 ppm for **1** in CDCl_3_). The *J*_P‐B_ coupling constant of 130.6 Hz, observed in both the ^31^P{^1^H} and ^11^B{^1^H} NMR spectra, is in line with typical P−B dative bonds (^1^ *J*_P‐B_=146 and 142 Hz for [(BBr_3_)_2_{*μ*‐Et_2_P(CH_2_)_2_PEt_2_}]^[16]^ and BBr_3_(PHPh_2_),^[17]^ respectively).

Compound **3** was heated at 80 °C overnight to afford compound **4** **a**, which exhibits a five‐membered ring (Scheme 2). Compound **4** **b**, a variant of **4** **a** merely with a bromide counteranion instead of tetrabromoborate, was prepared by heating a 1 : 1 mixture of **1** and BBr_3_⋅SMe_2_ in CDCl_3_. In the ^1^H NMR spectrum of **4** **b**, the resonance for the phosphonium proton is observed at 7.58 ppm, displaying a *dt* splitting pattern (^1^ *J*_P‐H_=485.8 Hz, ^3^ *J*_H‐H_=5.0 Hz), supporting the assignment of the CH_2_‐PH connectivity. The two peaks at 37.5 (^1^ *J*_P‐H_=485.9 Hz) and 18.4 ppm (m) in the ^31^P NMR spectrum correspond to the phosphonium and phosphine‐borane centers, respectively.

**Figure 4:**
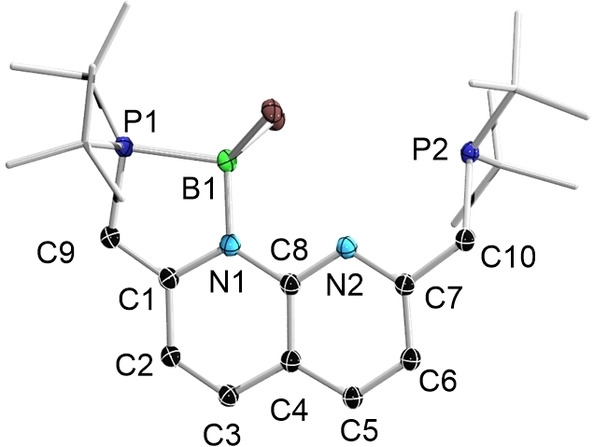
Solid‐state structure of the cation of **4** **b** (hydrogen atoms, and ellipsoids of the *t*Bu groups, are omitted for clarity). Thermal ellipsoids are set at the 50 % probability level.

**Figure 5:**
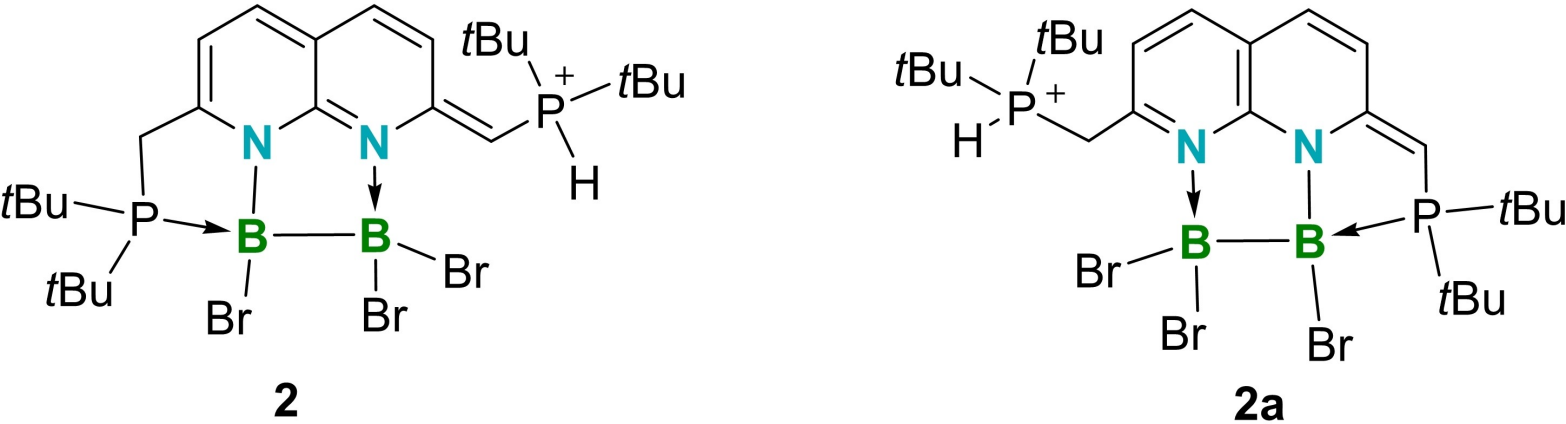
Coordination mode of **2** and its isomer **2** **a**.

The solid‐state structure of **4** **b** is depicted in Figure 4. It should be highlighted that, in compound **4**, the phosphorus atom adjacent to the dearomatized ring functions as a Lewis base while the opposite phosphorus atom functions as a Brønsted base, a bonding mode that is the opposite of that observed in **2**. Triggered by the different coordination modes of the deprotonated napy units in **2** and **4**, we calculated the energy difference between **2** and its isomer **2** **a** (Figure 5) at the PBE0‐D3(BJ)/6‐31+G**/LanL2DZ(Br) level of theory. These calculations showed that **2** is energetically more stable than its isomer **2** **a** by 12.2 kcal/mol in the gas phase. Similar results were found in chloroform medium, where the free energy of **2** is 9.2 kcal/mol more negative than that of **2** **a** at the SMD(CHCl_3_)/PBE0‐D3(BJ)/6‐311++G**/LanL2DZ(Br) level.

**Scheme 3:**
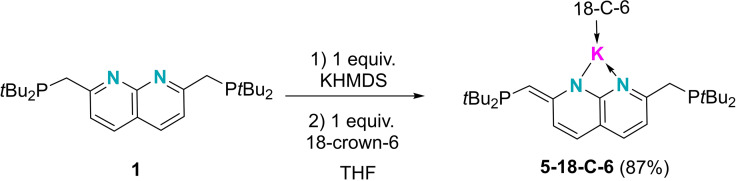
The deprotonation of **1** by KHMDS.

Given the propensity of **1** to undergo deprotonation of a methylene group, we also tested the possibility of deprotonating **1** and subsequently using these pre‐deprotonated species as reagents to bind boron species. At room temperature, an equimolar mixture of **1**, potassium bis(trimethylsilyl)amide (KHMDS) and 18‐crown‐6 in THF generated **5–18‐C‐6** (Scheme 3) as a dark red solution, which displays two signals in ^31^P{^1^H} NMR spectrum at 28.0 (PCH_2_) and 13.4 ppm (PCH=C) accompanied by the disappearance of the signal for free ligand **1** (34.0 ppm). A signal corresponding to the exocyclic C=CH proton was detected at 4.68 ppm in the ^1^H NMR spectrum, slightly downfield of that of the mono‐dearomatized species NDPCu_2_‐H (4.29 ppm)^[12]^ (Figure 1).

**Figure 6:**
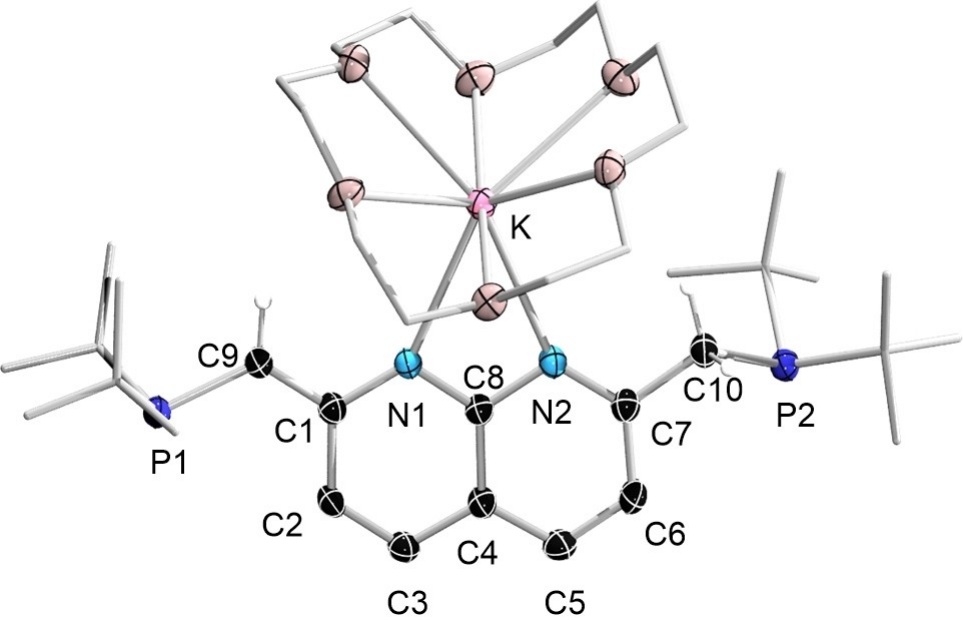
Solid‐state structure of **5‐18‐C‐6** (hydrogen atoms except for those on C9 and C10, and ellipsoids of the *t*Bu groups, are omitted for clarity). Thermal ellipsoids are set at the 50 % probability level.

Single crystals of this compound (**5‐18‐C‐6**) suitable for X‐ray diffraction analysis were obtained by recrystallization from THF/hexane at room temperature. In the solid‐state structure of **5–18‐C‐6** (Figure 6), the two six‐membered rings remain almost planar with sums of bond angles of 719.82° (the ring containing N1) and 719.89° (the ring containing N2). Meanwhile, the bonds within the N1‐containing ring have a higher level of bond length alternation than that within compound **1**, a characteristic sign of dearomatization. The C1‐C9 distance (1.384(4) Å) is significantly shorter than the definitive C7‐C10 single bond (1.514(4) Å). Besides the crown ether, the potassium atom is stabilized only by the two nitrogen atoms, with very similar K–N distances (K1‐N1: 2.848(3); K1‐N2: 2.834(3) Å).

**Scheme 4:**
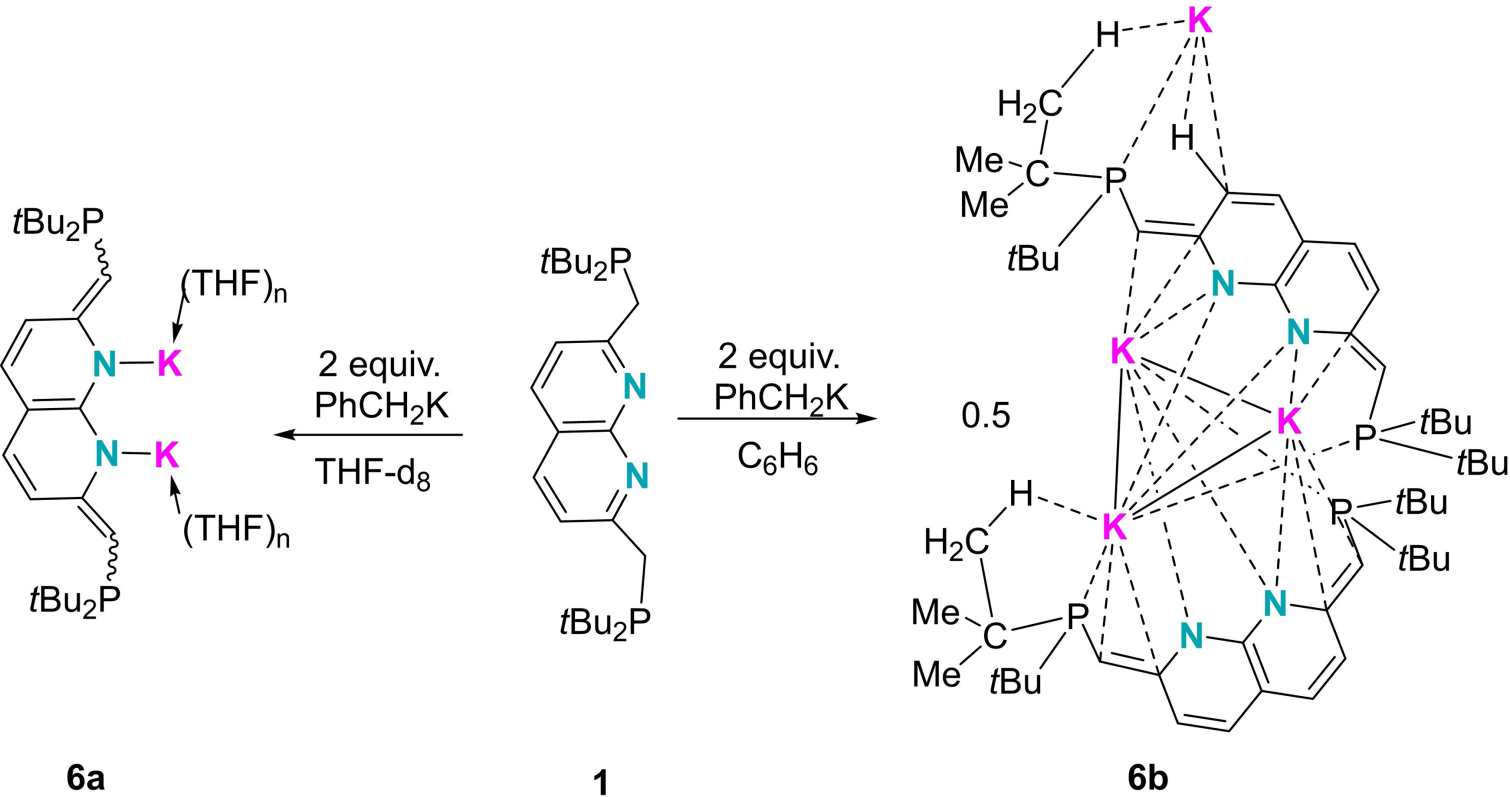
The synthesis of **6** **a** and **6** **b** by two‐fold deprotonation.

The deprotonation of the second CH_2_ group was found to require a Brønsted base stronger than KHMDS, such as benzyl potassium (PhCH_2_K). A mixture of **1** and two molar equivalents of PhCH_2_K in THF gave a light orange solution to furnish the double deprotonated species **6** **a** (Scheme 4). In the ^1^H NMR spectrum of **6** **a**, no signal corresponding to a CH_2_ unit is observed and the PC*H*=C proton signal (3.16 ppm) is found significantly upfield of that of **5** (4.68 ppm). The ^31^P{^1^H} NMR spectrum of **6** **a** exhibits only a broad resonance at 11.8 ppm, suggesting *C_2_* symmetry. Unfortunately, recrystallization of **6** **a** repeatedly generated compound **5** as determined by ^31^P NMR spectroscopy (i. e. 29.0 ppm in C_6_D_6_), precluding our efforts to structurally characterize this species. However, attempts to synthesize dipotassium salts of **1** in non‐coordinating solvents such as benzene led to several samples of dark‐red single crystals suitable for X‐ray study which were assigned to the dipotassium species **6** **b** (Scheme 4).

**Figure 7:**
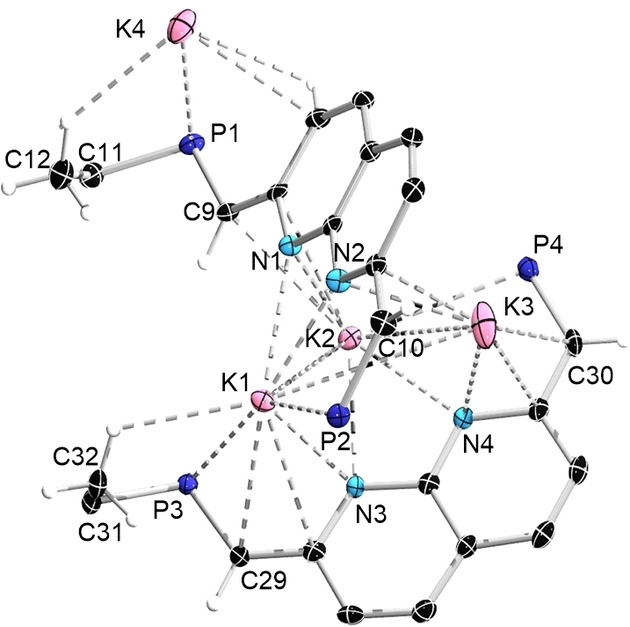
Solid‐state structure of **6** **b** (the carbon atoms of *t*Bu groups except for C11, C12 C31, C32 and hydrogen atoms except for those on C9, C10, C12, C29, C30, C32 are omitted for clarity). Thermal ellipsoids are set at the 50 % probability level.

In the solid state, **6** **b** contains two distinct ligands and four potassium atoms, all of which with a significantly different coordination environment. In one of the ligands, the two phosphine units are oriented in a *trans* fashion with respect to the napy core (P1 and P2, Figure 7), the other adopting a *cis* formation (P3 and P4). Due to the absence of coordinating solvents, the phosphines are found to coordinate to the potassium centers. The four NC=CH bonds range from 1.377 to 1.397 Å, fitting well with those of NDP‐Cu_2_ derivatives with full‐dearomatization (1.380(5)‐1.399(5) Å,[^4f^, ^12^] Scheme 1) and confirming the successful two‐fold deprotonation.

**Scheme 5:**
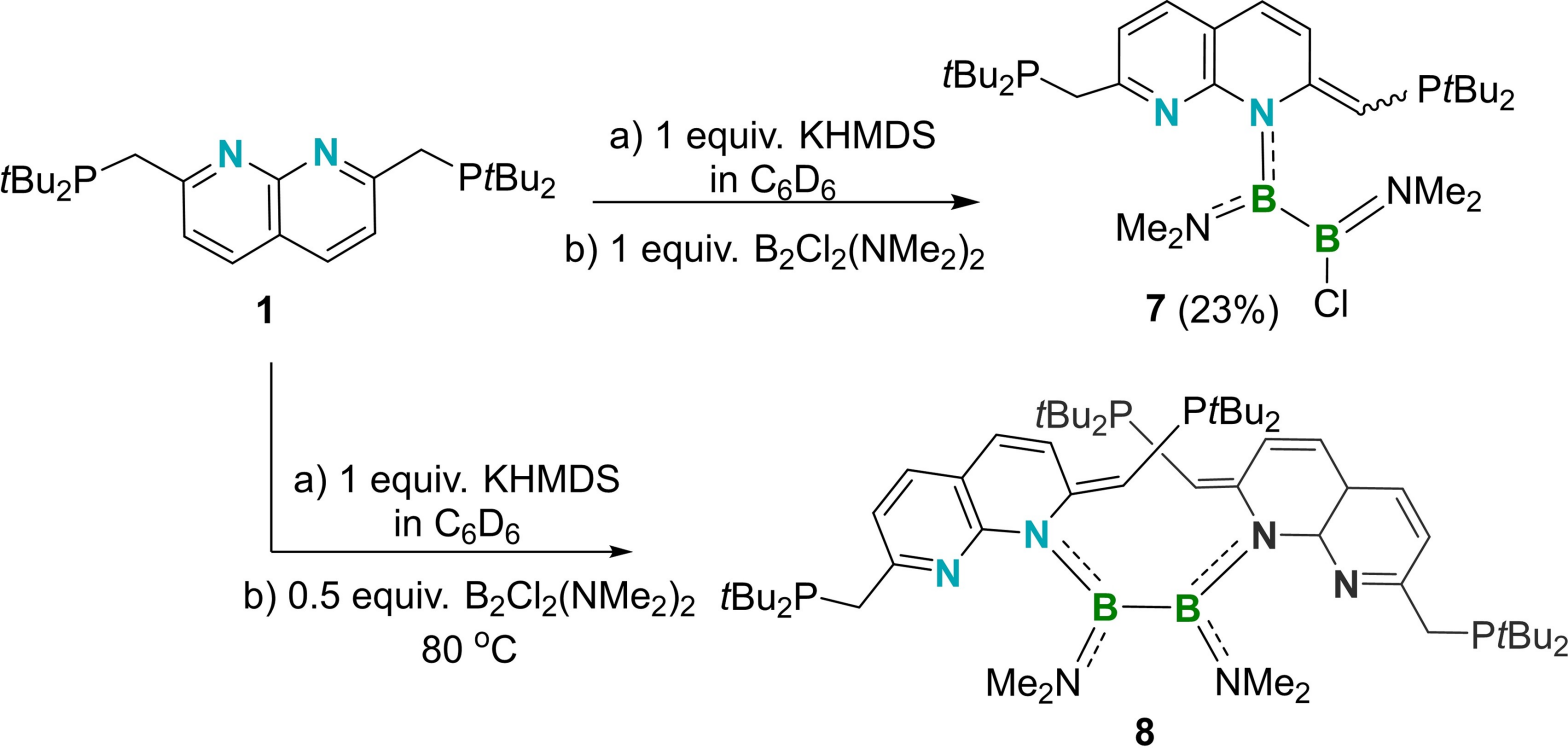
The synthesis of compounds **7** and **8**.

We subsequently set out to introduce boron units by salt elimination using **5** and the 1,2‐diamino(dihalo)diborane(4) 1,2‐B_2_(NMe_2_)_2_Cl_2_. Depending on the amount of B_2_(NMe_2_)_2_Cl_2_ employed, either a 1 : 1 or a 2 : 1 product was obtained. The mono‐potassium reagent **5** was prepared in situ and used without separation of the byproduct bis(trimethylsilyl)amine. Addition of one molar equivalent of B_2_(NMe_2_)_2_Cl_2_ to a benzene solution of **5** at ambient temperature, followed by heating at 80 °C for 2 h, led to the color fading from red to orange, generating the 1 : 1 product **7** (Scheme 5).

In the ^31^P{^1^H} NMR spectrum of **7**, the signals corresponding to the CH_2_ *P* and CH*P* phosphorus nuclei were found at 34.0 and 7.6 ppm, respectively. A single broad resonance at 38.5 ppm was found in the ^11^B{^1^H} NMR spectrum, nearly identical to that of B_2_(NMe_2_)_2_Cl_2_ (38.0 ppm). However, the full consumption of B_2_(NMe_2_)_2_Cl_2_ was supported by the ^1^H NMR spectrum, which suggests the presence of three NMe_2_ environments (signals at 2.88, 2.80, and 2.59 ppm with an intensity ratio of 6 : 3 : 3).

**Figure 8:**
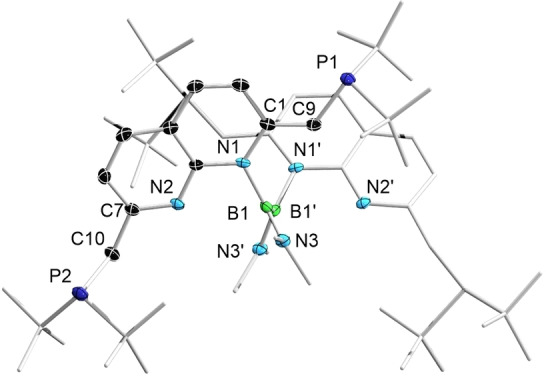
Solid‐state structure of **8** (hydrogen atoms, all of the ellipsoids of the *t*Bu groups, and most of the ellipsoids of the NDP ligand at the rear are omitted for clarity). Thermal ellipsoids are set at the 50 % probability level.

During the recrystallization of **7**, several yellow crystals were obtained corresponding to compound **8**, comprised of two napy ligands and one B_2_N_2_ unit. Heating a mixture of B_2_(NMe_2_)_2_Cl_2_ and two molar equivalents of **5** at 80 °C overnight provided a rational synthesis of **8**. Due to repeated decomposition of compound **8** to **1**, it was characterized as a mixture and only the ^31^P{^1^H} and ^1^H NMR spectra were recorded. The ^31^P{^1^H} NMR spectrum of **8** is almost identical to that of compound **7**, as the signals for the CH_2_ *P* and CH*P* nuclei appear at 32.6 and 6.0 ppm. The signals for the NMe_2_ groups in the ^1^H NMR spectrum allow distinction of **8** from **7** by their chemical shift differences (**8**: 2.78 and 2.58; **7**: 2.88, 2.80 and 2.59) and the NC=C*H*P to N(C*H*_3_)_2_ intensity ratio (2 : 12 for **8** compared with 1 : 12 for **7**). Unfortunately, the X‐ray diffraction data of **8** was poor, but sufficient to determine the connectivity of the compound (Figure 8).

## Conclusion

We have developed two synthetic routes to 1,8‐naphthyridine‐diphosphine‐based boron compounds. The first is the direct coordination of NDP to boron‐centered Lewis acids, accompanied by the simultaneous dearomatization of one of the naphthyridine rings. Following this method, NDP‐boron compounds featuring both binuclear bridging and monodentate coordination modes have been prepared. Notably, the reaction of NDP with B_2_Br_4_(SMe_2_) provided a rare example of a main‐group species with four fused rings, three of which are roughly coplanar. The second pathway involves the preparation of a monometallated NDP species followed by salt elimination with a 1,2‐diaminodihalodiborane(4). Both mono‐ and diligated products can be prepared using differing amounts of the diborane(4).

### X‐ray Crystallography

Deposition Number(s) 2099390 (for **2**), 2099391 (for **4** **b**), 2099392 (for **5–18‐C‐6**), 2099393 (for **6** **b**), 2099394 (for **8**) contain(s) the supplementary crystallographic data for this paper. These data are provided free of charge by the joint Cambridge Crystallographic Data Centre and Fachinformationszentrum Karlsruhe Access Structures service.

## Conflict of interest

The authors declare no conflict of interest.

## Supporting information

As a service to our authors and readers, this journal provides supporting information supplied by the authors. Such materials are peer reviewed and may be re‐organized for online delivery, but are not copy‐edited or typeset. Technical support issues arising from supporting information (other than missing files) should be addressed to the authors.

Supporting Information
