# Supplementary material for: Diphosphino‐Functionalized 1,8‐Naphthyridines: a Multifaceted Ligand Platform for Boranes and Diboranes
Source: Chemistry. 2021 Oct 11;27(63):15751–6. doi: 10.1002/chem.202102721 (PMC9292315; doi:10.1002/chem.202102721)
Supplement: Supplementary file 1 — Supporting Information [file CHEM-27-15751-s001.pdf]

Chemistry–A European Journal

Supporting Information

Diphosphino-Functionalized 1,8-Naphthyridines: a Multifaceted Ligand Platform for Boranes and Diboranes

Jingjing Cui, Maximilian Dietz, Marcel Härterich, Felipe Fantuzzi, Wei Lu, Rian D. Dewhurst, and Holger Braunschweig*

Contents

Methods and materials 2

Synthetic procedures 3

NMR spectra of compounds 9

X-ray crystallographic data 39

HRMS spectra of compounds 45

Computational details 53

Cartesian coordinates 54

References 60

Methods and materials

All manipulations were performed either under an atmosphere of dry argon or *in vacuo* using standard Schlenk line or glovebox techniques. Deuterated solvents were dried over molecular sieves and degassed by three freeze-pump-thaw cycles prior to use. All other solvents were distilled and degassed from appropriate drying agents. Both deuterated and non-deuterated solvents were stored under argon over activated 4 Å molecular sieves. NMR spectra were acquired either on a Bruker Avance 500 or a Bruker Avance 400 NMR spectrometer. Chemical shifts (δ) are yield in ppm and internally referenced to the carbon nuclei ($^{13}C\{^{1}H\}$) or residual protons (^{1}H) of the solvent. Heteronuclei NMR spectra are referenced to external standards (^{11}B: $BF_3{\cdot}OEt_2$; ^{31}P NMR: 85% H_3PO_4). High-resolution mass spectrometry (HRMS) data were obtained from a Thermo Scientific Exactive Plus spectrometer.

Solvents and reagents were purchased from Sigma-Aldrich, Alfa Aesar or Fluorochem. Ligand **1**,[1] $B_2Br_4(SMe_2)_2$,[2] and $PhCH_2K$[3] were synthesized using literature procedures.

Synthetic procedures

(LB$_2$Br$_3$)Br, 2:

Free ligand **1** (44.7 mg, 0.1 mmol) and $B_2Br_4(SMe_2)_2$ (46.6 g, 0.1 mmol) were mixed before adding 2 mL of $CHCl_3$. The reaction was stirred for 30 min and then all volatiles were removed under vacuum. The yellow powder was washed with benzene (three times with 2 mL) and thoroughly dried to give compound **2** as a bright yellow powder (75.1 mg).

Yield: 95%.

^{1}H NMR (400 MHz, $CDCl_3$) δ: 9.08 (dd, $^1J_{P-H}$ = 474.4 Hz, $^3J_{H-H}$ = 13.3 Hz, 1H, P-*H*), 7.83 (d, $^3J_{H-H}$ = 9.6 Hz, 1H, Ar-*H*), 7.72 (d, $^3J_{H-H}$ = 7.6 Hz, 1H, Ar-*H*), 7.32 (d, $^3J_{H-H}$ = 7.6 Hz, 1H, Ar-*H*), 7.04 (d, $^3J_{H-H}$ = 9.6 Hz, 1H, Ar-*H*), 6.18 (dd, $^3J_{H-H}$ = 13.3 Hz, $^2J_{P-H}$ = 6.9 Hz, 1H, C=C*H*), 4.19 (dd, $^2J_{H-H}$ = 17.6 Hz, $^2J_{P-H}$ = 7.1 Hz, 1H, PCH_2), 3.92 (dd, $^2J_{H-H}$ = 17.6 Hz, $^2J_{P-H}$ = 9.9 Hz, 1H, PCH_2), 1.78 (d, $^3J_{P-H}$ = 15.4 Hz, 9H, CMe_3), 1.56 (d, $^3J_{P-H}$ = 4.9 Hz, 9H, CMe_3), 1.52 (d, $^3J_{P-H}$ = 4.9 Hz, 9H, CMe_3), 1.21 - 1.60 (br, 9H, CMe_3).

^{13}C NMR (101 MHz, $CDCl_3$) δ: 152.7 (d, J = 6.4 Hz, Ar-*C*), 151.0 (dd, J = 9.3, 2.3 Hz, Ar-*C*), 149.6 (d, J = 1.3 Hz, Ar-*C*), 139.6 (s, Ar-*C*H), 128.3 (s, Ar-*C*H), 127.8 (d, J = 8.7 Hz, Ar-*C*H), 118.7 (s, Ar-*C*), 115.4 (d, J = 5.3 Hz, Ar-*C*H), 76.1 (s, C=*C*H), 35.7 (d, $^1J_{P-C}$ = 12.6 Hz, *C*Me$_3$), 35.5 (d, $^1J_{P-C}$ = 7.0 Hz, *C*Me$_3$), 34.9 (d, $^1J_{P-C}$ = 5.5 Hz, *C*Me$_3$), 34.5 (d, $^1J_{P-C}$ = 5.2 Hz, *C*Me$_3$), 31.0 (s, CMe_3), 28.1 - 28.9 (br, CMe_3), 27.6 (s, CMe_3).

^{31}P NMR (162 MHz, $CDCl_3$) δ: 56.0 (s), 19.8 (d, $^1J_{P-H}$ = 474.4 Hz).

^{11}B NMR (128 MHz, $CDCl_3$) δ: 0.3 (br).

HRMS: Calcd for $[C_{26}H_{43}B_2Br_4N_2P_2]^+$ 786.9775, found 786.9757, method FTMS + p ESI. We are unsure how the elimination of hydride happened but in the same measurement, we also find three other peaks with reasonable fragmentation patterns, so we believe this HRMS is reliable.

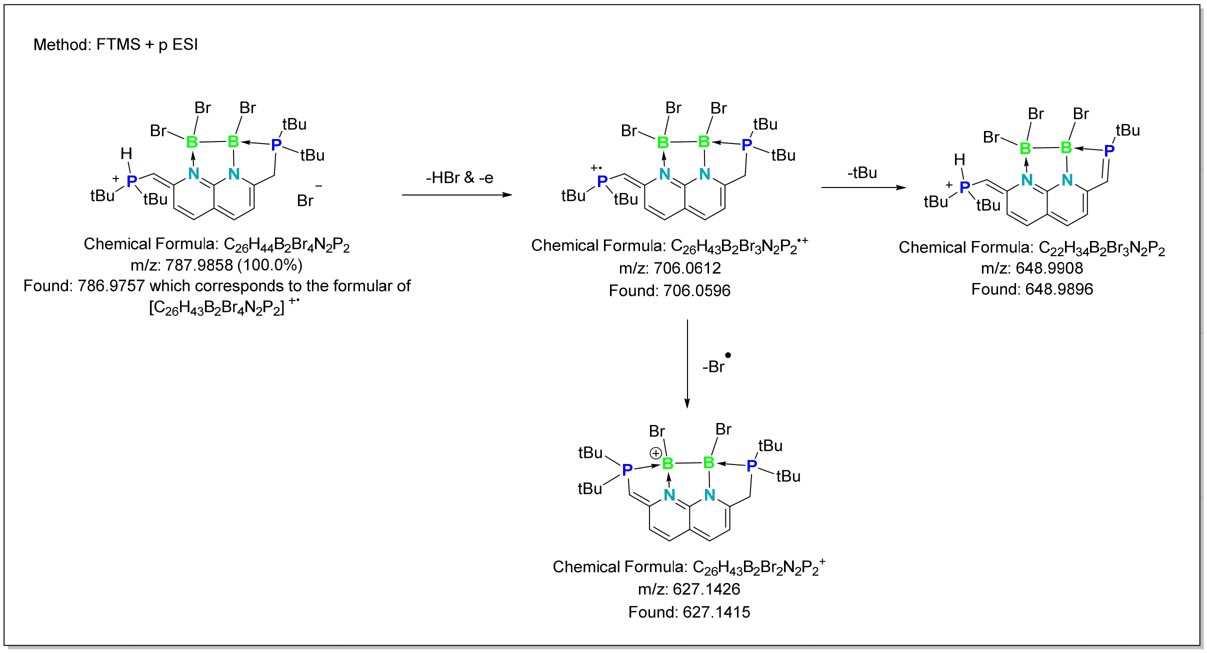

L((BBr_3)$_2$, 3:

This was prepared via an NMR-scale reaction as **3** is only used as the intermediate for the synthesis of **4a**. Free ligand **1** (13.5 mg, 0.03 mmol) and $BBr_3(SMe_2)$ (18.6 mg, 0.06 mmol) were mixed before adding $CDCl_3$, and the NMR spectrum was recorded shortly afterwards.

Yield: not calculated.

^{1}H NMR (400 MHz, $CDCl_3$) δ: 8.19 (d, $^1J_{H\text{-}H}$ = 8.3 Hz, 2H), 7.80 (d, $^1J_{H\text{-}H}$ = 8.3 Hz, 2H), 4.28 (d, $^2J_{P\text{-}H}$ = 12.0 Hz, 4H), 1.76 (d, $^3J_{P\text{-}H}$ = 14.0 Hz, 36H).

^{13}C{^{1}H} NMR (101 MHz, $CDCl_3$) δ: 158.9 (d, J = 8.1 Hz, Ar-*C*), 154.3 (s, Ar-*C*), 137.7 (s, Ar-*C*H), 125.7 (d, J = 5.3 Hz, Ar-*C*H), 120.3 (s, Ar-*C*), 39.0 (d, $^1J_{P\text{-}C}$ = 22.0 Hz, *C*Me3), 31.0 (s, C*Me*3), 30.5 (d, $^1J_{P\text{-}C}$ = 27.0 Hz, P-*C*H2). Note: compound **3** slowly isomerizes to **4a** at room temperature during the recording of the ^{13}C{^{1}H} NMR spectrum, as indicated by the emergence of peaks at 29.0 and 28.0 ppm (Figure S7).

^{31}P{^{1}H} NMR (162 MHz, $CDCl_3$) δ: 9.9 (dd, J = 261.4 Hz, $^1J_{B\text{-}P}$ = 130.6 Hz).

^{11}B{^{1}H} NMR (128 MHz, $CDCl_3$) δ: −15.0 (d, $^1J_{P\text{-}B}$ = 130.6 Hz).

($LBBr_2$)(BBr_4), 4a:

In-situ-generated **3** in $CDCl_3$ was heated at 80 °C overnight and then all volatiles were removed to give 25.4 mg of **4a** as a deep yellow solid. This solid is a mixture of **4a** and the protonated ligand **[LH]$^+$** in a ratio of ca. 5:1 as shown in the ^{1}H and ^{31}P{^{1}H} NMR spectra.

Yield: 89%.

^{1}H NMR of **4a**: (400 MHz, $CDCl_3$) δ: 7.73 (dt, $^{1}J_{P\text{-}H}$ = 490.6 Hz, $^{3}J_{H\text{-}H}$ = 4.9 Hz, 1H, P*H*), 7.49 (d, $^{3}J_{H\text{-}H}$ = 7.6 Hz, 1H, Ar-*H*), 7.45 (d, $^{3}J_{H\text{-}H}$ = 7.6 Hz, 1H, Ar-*H*) 6.90 (dd, $^{3}J_{H\text{-}H}$ = 9.3 Hz, $^{4}J_{P\text{-}H}$ = 1.8 Hz, 1H, Ar-*H*), 6.49 (d, $^{3}J_{H\text{-}H}$ = 9.3 Hz, 1H, Ar-*H*), 4.43 (d, $^{2}J_{P\text{-}H}$ = 7.3 Hz, 1H, C=C-*H*), 4.23 (dd, $^{2}J_{P\text{-}H}$ = 12.2 Hz, $^{3}J_{H\text{-}H}$ = 4.9 Hz, 2H, PCH_2), 1.60 (d, $^{3}J_{P\text{-}H}$ = 10.8 Hz, 18H, CMe_3), 1.56 (d, $^{3}J_{P\text{-}H}$ = 12.6 Hz, 18H, CMe_3).

^{13}C{^{1}H} NMR of **4a**: (101 MHz, $CDCl_3$) δ: 161.59 (d, *J* = 10.1 Hz), 151.67 (d, *J* = 9.6 Hz), 149.61 (d, *J* = 9.1 Hz), 136.89 (s), 132.89 (s), 122.69 (d, *J* = 14.5 Hz), 118.42 (d, *J* = 7.3 Hz), 117.68 (s), 70.46 (d, *J* = 61.4 Hz), 37.30 (d, *J* = 30.8 Hz), 33.26 (d, *J* = 35.5 Hz), 28.51 (d, *J* = 96.3 Hz), 24.86 (d, *J* = 46.4 Hz).

^{31}P NMR of **4a**: (162 MHz, $CDCl_3$) δ: 36.3 (dm, $^{1}J_{H\text{-}P}$ = 490.6Hz), 18.5 (br).

^{11}B{^{1}H} NMR of **4a**: (128 MHz, $CDCl_3$) δ: −5.5 (d, $^{1}J_{P\text{-}B}$ = 127.4 Hz), −24.1 (s).

($LBBr_2$)Br, 4b:

Free ligand **1** (26.8 mg, 0.06 mmol) and $BBr_3(SMe_2)$ (18.8 mg, 0.06 mmol) were mixed before adding 5 mL $CHCl_3$, and the mixture was heated at 80 °C overnight. All volatiles were removed and the yellow solid received was washed with 5 mL benzene to give 29.5 mg of **4b**.

Yield: 71%.

^{1}H NMR: (400 MHz, $CDCl_3$) δ: 7.68 (d, $^{3}J_{H\text{-}H}$ = 7.6 Hz, 1H, Ar-*H*), 7.58 (dt, $^{1}J_{P\text{-}H}$ = 485.8, $^{3}J_{H\text{-}H}$ = 5.0 Hz, 1H, P-*H*). 7.39 (d, $^{3}J_{H\text{-}H}$ = 7.6 Hz, 1H, Ar-*H*), 6.86 (dd, $^{3}J_{H\text{-}H}$ = 9.4 Hz, $^{4}J_{P\text{-}H}$ = 1.8 Hz, 1H, Ar-*H*), 6.45 (d, $^{3}J_{H\text{-}H}$ = 9.4 Hz, 1H, Ar-*H*), 4.63 (dd, $^{2}J_{P\text{-}H}$ = 12.5 Hz, $^{3}J_{H\text{-}H}$ = 5.0 Hz, 2H, PCH_2), 4.39 (d, $^{2}J_{P\text{-}H}$ = 7.3 Hz, 1H, C=C*H*), 1.56 (d, $^{3}J_{P\text{-}H}$ = 14.6 Hz, 18H), 1.54 (d, $^{3}J_{P\text{-}H}$ = 16.4 Hz, 18H).

^{13}C{^{1}H} NMR: (101 MHz, $CDCl_3$) δ: 161.6 (d, $J_{P\text{-}C}$ = 10.2 Hz, Ar-C), 151.5 (d, $J_{P\text{-}C}$ = 9.6 Hz, Ar-C), 150.5 (d, $J_{P\text{-}C}$ = 9.3 Hz, Ar-C), 136.6 (s, Ar-CH), 132.9 (s, Ar-CH), 122.4 (d, $J_{P\text{-}C}$ = 14.2 Hz, Ar-CH), 118.8 (d, $J_{P\text{-}C}$ = 7.3 Hz, Ar-CH), 117.4 (d, $J_{P\text{-}C}$ = 1.3 Hz, Ar-C), 70.0 (d, $J_{P\text{-}C}$ = 61.7 Hz, C=*C*H), 37.2 (d, $J_{P\text{-}C}$ = 30.8 Hz, *C*Me_3), 33.1 (d, $J_{P\text{-}C}$ = 36.0 Hz, *C*Me_3), 28.9 (s, CMe_3), 28.0 (s, CMe_3), 24.9 (d, $J_{P\text{-}C}$ = 45.4 Hz, PCH_2).

^{31}P NMR: (162 MHz, $CDCl_3$) δ: 37.5 (d, $^{1}J_{P\text{-}H}$ = 485.9 Hz, *P*-H), 18.4 (m).

^{11}B{^{1}H} NMR: (128 MHz, $CDCl_3$) δ: −5.6 (d, $^{1}J_{B\text{-}P}$ = 137.3 Hz).

HRMS: Calcd for $[C_{26}H_{44}N_2P_2BBr_2]^+$ 617.1414, found 617.1405, method FTMS + p ESI.

LK-18C6, 5-18-C-6:

Free ligand **1** (0.223 g, 0.5 mmol) and KHMDS (0.100 g, 0.5 mmol) were mixed in THF at room temperature before adding 18-crown-6 (0.132 g, 0.5 mmol). Reaction was stirred for 30

min and then all volatiles were removed. The dark red solid received was washed with 15 mL cold pentane to give 0.326 g of **5-18-C-6** as a fine powder. The sample received contains a small amount of **1** and the amount of **1** varies between batches. The NMR data following are recorded for the in-situ-generated **5-18-C-6**.

Yield: 87%.

^{1}H NMR: (400 MHz, C_6D_6) δ: 8.07 (dd, $^{1}J_{H\text{-}H}$ = 9.3 Hz, $^{4}J_{P\text{-}H}$ = 5.0 Hz, 1H, Ar-*H*), 6.98 – 6.83 (m, 2H, Ar-*H*), 6.60 (d, $^{1}J_{H\text{-}H}$ = 9.3 Hz, 1H, Ar-*H*), 4.68 (d, $^{2}J_{P\text{-}H}$ = 8.1 Hz, 1H, PC*H*=C), 2.92 (d, $^{2}J_{P\text{-}H}$ = 2.9 Hz, 2H, PCH_2), 1.47 (d, $^{2}J_{P\text{-}H}$ = 10.5 Hz, 18H), 1.22 (d, $^{2}J_{P\text{-}H}$ = 10.4 Hz, 18H).

^{13}C{^{1}H} NMR: (101 MHz, C_6D_6) δ: 166.6 (d, $J_{P\text{-}C}$ = 33.2 Hz, Ar-*C*), 163.9 (dd, $J_{P\text{-}C}$ = = 3.5, 1.4 Hz, Ar-*C*), 159.4 (d, $J_{P\text{-}C}$ = = 15.1 Hz, Ar-*C*), 132.4 (s, Ar-*C*H), 128.6 (s, Ar-*C*H), 123.9 (d, $J_{P\text{-}C}$ = 30.7 Hz, Ar-*C*H), 116.6 – 116.5 (m, Ar-*C*), 108.4 (d, $J_{P\text{-}C}$ = 16.4 Hz, Ar-*C*H), 80.7 (d, $J_{P\text{-}C}$ = 3.8 Hz, P*C*H=C), 32.6 (d, $^{1}J_{P\text{-}C}$ = 18.4 Hz, *C*Me3), 31.7 (d, $^{1}J_{P\text{-}C}$ = 24.5 Hz, *C*Me3), 30.9 (d, $^{2}J_{P\text{-}C}$ = 14.6 Hz, C*Me*3), 30.3 (d, $^{2}J_{P\text{-}C}$ = 13.7 Hz, C*Me*3).

^{31}P{^{1}H} NMR: (162 MHz, C_6D_6) δ: 28.0 (s), 13.4 (s).

HRMS: Calcd for $[C_{26}H_{43}N_2P_2K+H]^+$ 485.2611, found 485.2602, method FTMS + p ESI.

LK_2, 6:

Free ligand **1** (8.9 mg, 0.02 mmol) and $PhCH_2K$ (5.2 mg, 0.02 mmol) were mixed in THF-d_8 at room temperature to give a pale orange solution. NMR data were recorded using this in-situ-generated sample. All attempts to purify **LK_2** provided only the monodeprotonated product.

Yield: not calculated.

^{1}H NMR: (500 MHz, THF-d_8) δ: 6.02 (d, $^{3}J_{H\text{-}H}$ = 8.4 Hz, 2H, Ar-*H*), 5.42 (d, $^{3}J_{H\text{-}H}$ = 8.4 Hz, 2H, Ar-*H*), 3.16 (d, $^{2}J_{P\text{-}H}$ = 8.1 Hz, 2H, C=C*H*P), 1.10 (d, *J* = 10.3 Hz, 36H, *t*Bu).

^{13}C{^{1}H} NMR: (126 MHz, THF-d_8) δ: 168.2 (d, $J_{P\text{-}C}$ = 21.1 Hz, Ar-*C*), 163.0 (s, Ar-*C*), 129.9 (s, Ar-*C*H), 110.2 (d, $J_{P\text{-}C}$ = 7.5 Hz, Ar-*C*H), 102.5 (s, Ar-*C*), 65.0 (d, $J_{P\text{-}C}$ = 5.4 Hz, C=*C*H-P), 33.1 (d, $J_{P\text{-}C}$ = 16.8 Hz, *C*Me3), 30.7 (d, $J_{P\text{-}C}$ = 13.7 Hz, C*Me*3).

^{31}P{^{1}H} NMR: (202 MHz, THF) δ: 11.8 (s).

Note: Compound **6** is too sensitive for both elemental analysis and HRMS.

LN_2B_2Cl, 7:

Free ligand **1** (44.7 mg, 0.1 mmol) and KHMDS (20.0 mg, 0.1 mmol) were mixed in 1 mL benzene at room temperature and the full consumption of **1** was confirmed by ^{31}P NMR spectroscopy. At room temperature, the solution of **LK** was added to a solution of $B_2(NMe_2)_2Cl_2$ (18.0 mg, 0.1 mmol, 0.5 mL benzene). The mixture was heated at 80 °C and

monitored by ^{31}P NMR spectroscopy until all **LK** was consumed (normally 2 h). The yellow solution received was filtered and recrystallized to give 13.4 mg of compound **7**. The sample received always contains small amount of **1** even when recrystallized in a glovebox.

Yield: 23%.

^{1}H NMR: (500 MHz, C_6D_6) δ: 7.88 (dd, $^3J_{H-H}$ = 9.7 Hz, $^4J_{P-H}$ = 5.5 Hz, 1H, Ar-*H*), 6.78 (dd, $^3J_{H-H}$ = 7.6 Hz, $^4J_{P-H}$ = 1.3 Hz, 1H, Ar-*H*), 6.68 (d, $^3J_{H-H}$ = 7.6 Hz, 1H, Ar-*H*), 6.18 (dd, $^3J_{H-H}$ = 9.7 Hz, $^5J_{H-H}$ = 1.4 Hz, 1H, Ar-*H*), 4.85 (dd, $^2J_{P-H}$ = 6.9 Hz, $^5J_{H-H}$ = 1.4 Hz, 1H, PC=C*H*), 2.88 (br, 7H, *6H from* N*Me*$_2$ *and one H of* PC*H*$_2$), 2.80–2.78 (m, 4H, N*Me*$_2$ and the *other H of* PC*H*$_2$) 2.59 (s, 3H, N*Me*), 1.37 (d, $^3J_{P-H}$ = 11.3 Hz, 9H, C*Me*$_3$), 1.31 (d, J = 11.1 Hz, 9H, C*Me*$_3$), 1.10 (dd, J = 10.6, 1.4 Hz, 18H, C*Me*$_3$).

^{13}C{^{1}H} NMR: (126 MHz, C_6D_6) δ: 160.3 (d, J_{P-C} = 14.1 Hz, *Ar-C*), 154.4 (d, J_{P-C} = 1.5 Hz, *Ar-C*), 151.8 (d, J_{P-C} = 30.5 Hz, *Ar-C*), 133.5 (s, *Ar-CH*), 126.6 (d, J_{P-C} = 2.1 Hz, *Ar-CH*), 124.7 (d, J_{P-C} = 32.1 Hz, *Ar-CH*), 115.6 (d, J_{P-C} = 10.2 Hz, *Ar-CH*), 114.5 (t, J_{P-C} = 1.8 Hz), 90.9 (d, J_{P-C} = 12.8 Hz, PC=*C*H), 41.2 (s, N*Me*$_2$), 40.8 (br, N*Me*$_2$), 38.7 (s, N*Me*$_2$), 32.6 (d, J_{P-C} = 17.3 Hz, *C*Me$_3$), 32.1 (d, J_{P-C} = 4.1 Hz, *C*Me$_3$), 31.9 (d, J_{P-C} = 3.9 Hz, *C*Me$_3$), 31.8 (d, J_{P-C} = 24.6 Hz, *C*Me$_3$), 31.6 (d, J_{P-C} = 26.1 Hz), 30.2 (d, J_{P-C} = 12.1 Hz, C*Me*$_3$), 30.1 (d, J_{P-C} = 3.9 Hz, C*Me*$_3$), 29.9 (d, J_{P-C} = 13.8 Hz, C*Me*$_3$).

^{31}P{^{1}H} NMR: (202 MHz, C_6D_6) δ: 34.0 (s, *P*CH$_2$), 7.6 (s, *P*C=CH).

^{11}B{^{1}H} NMR: (160 MHz, C_6D_6) δ: 38.5 (br).

HRMS: Calcd for $[C_{30}H_{55}B_2ClN_4P_2]^{+\cdot}$, 590.3771, found 590.3758, method FTMS + p ESI.

$L_2N_2B_2$, 8:

Free ligand **1** (8.9 mg, 0.02 mmol) and KHMDS (4.0 mg, 0.02 mmol) were mixed in 0.3 mL C_6D_6 at room temperature and the full consumption of **1** was confirmed by ^{31}P NMR spectroscopy. At room temperature, the solution of **LK** was added to the solution of $B_2(NMe_2)_2Cl_2$ (1.8 mg, 0.01 mmol, 0.2 mL of C_6D_6). The mixture was heated at 80 °C overnight and monitored by ^{31}P NMR spectroscopy until all **LK** was consumed. The yellow solution received was a mixture of **8** and **1** and directly used for NMR spectroscopy. All purification operations provided only **1** as the major product.

Yield: not calculated.

^{1}H NMR: (400 MHz, C_6D_6) δ 7.25 (dd, $^3J_{H-H}$ = 9.6 Hz, $^4J_{P-H}$ = 4.2 Hz, 2H, Ar-*H*), 6.24 (dd, $^3J_{H-H}$ = 8.9 Hz, J = 1.7 Hz, 2H, Ar-*H*), 6.12 (dd, $^3J_{H-H}$ = 9.6 Hz, J = 1.3 Hz, 2H, Ar-*H*), 5.75 (dd, $^3J_{H-H}$ = 8.9 Hz, $^4J_{P-H}$ = 0.7 Hz, 2H, Ar-*H*), 4.82 (d, $^2J_{P-H}$ = 6.9 Hz, 2H, PC=C*H*), 3.60 (d, $^2J_{P-H}$ = 3.6 Hz, 2H, one type of H of PCH$_2$), 2.78 (s, 6H, N*Me*$_2$), 2.58 (s, 6H, N*Me*$_2$), 1.32 (d, J = 11.1

Hz, 18H, C*Me*$_3$), 1.28 (d, *J* = 5.5 Hz, 18H, C*Me*$_3$), 1.25 (d, *J* = 3.9 Hz, 18H, C*Me*$_3$), 1.02 (d, *J* = 12.1 Hz, 18H, C*Me*$_3$). Note: the signal for the second H nucleus of PCH_2 is suspected to be overlapped with the broad peak at ca. 2.99−2.80 ppm.

$^{31}P\{^1H\}$ NMR: (202 MHz, C_6D_6) δ: 32.6 (br, *P*CH_2), 6.0 (s, *P*C=CH)

^{11}B NMR: too broad to be detected.

HRMS: Theory data for $[C_{56}H_{98}B_2N_6P_4]^{+\cdot}$ 1000.6984, found 1000.6978, method FTMS + p ESI.

NMR spectra of compounds

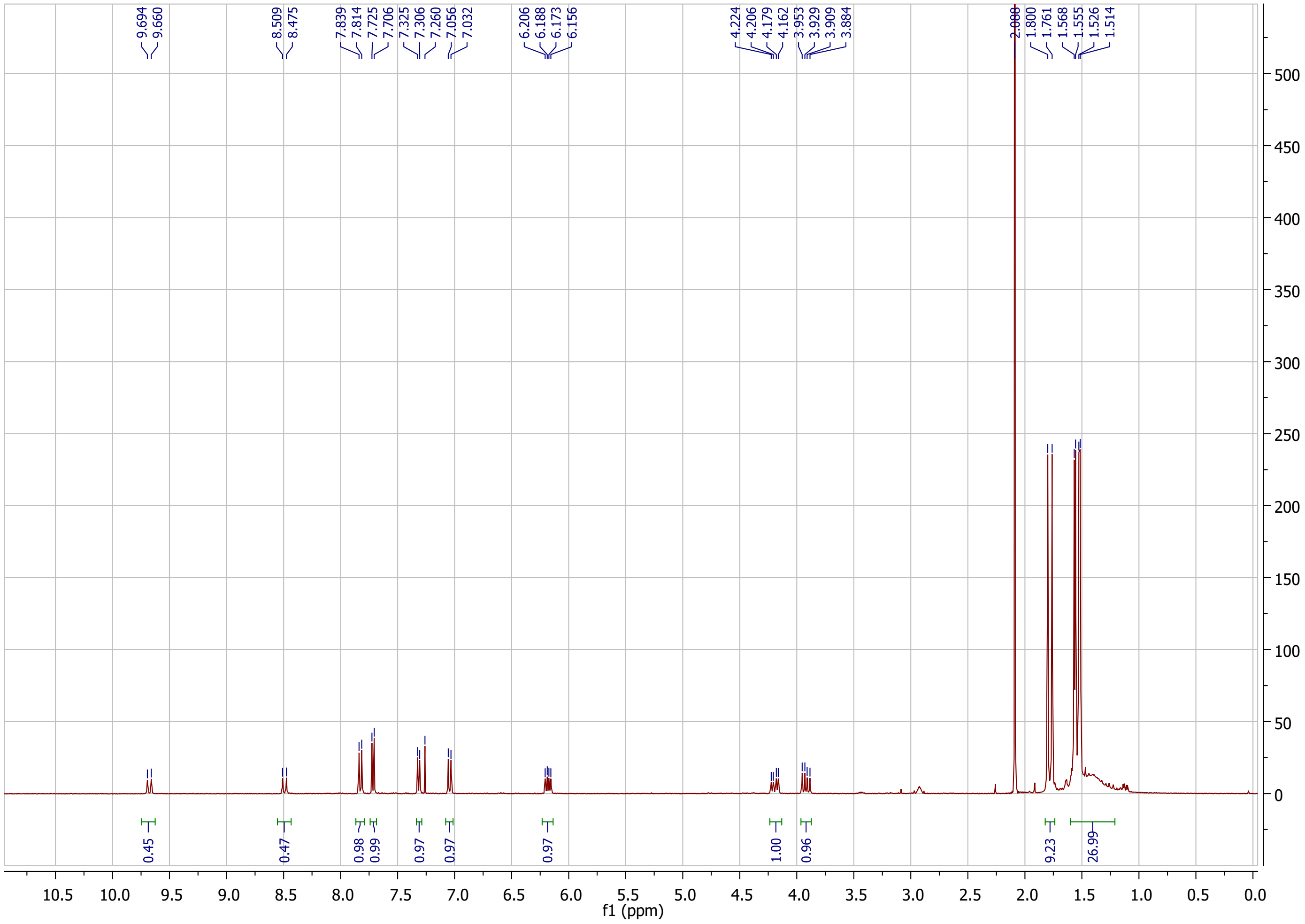

Figure S1. ^{1}H NMR spectrum of **2** in $CDCl_3$.

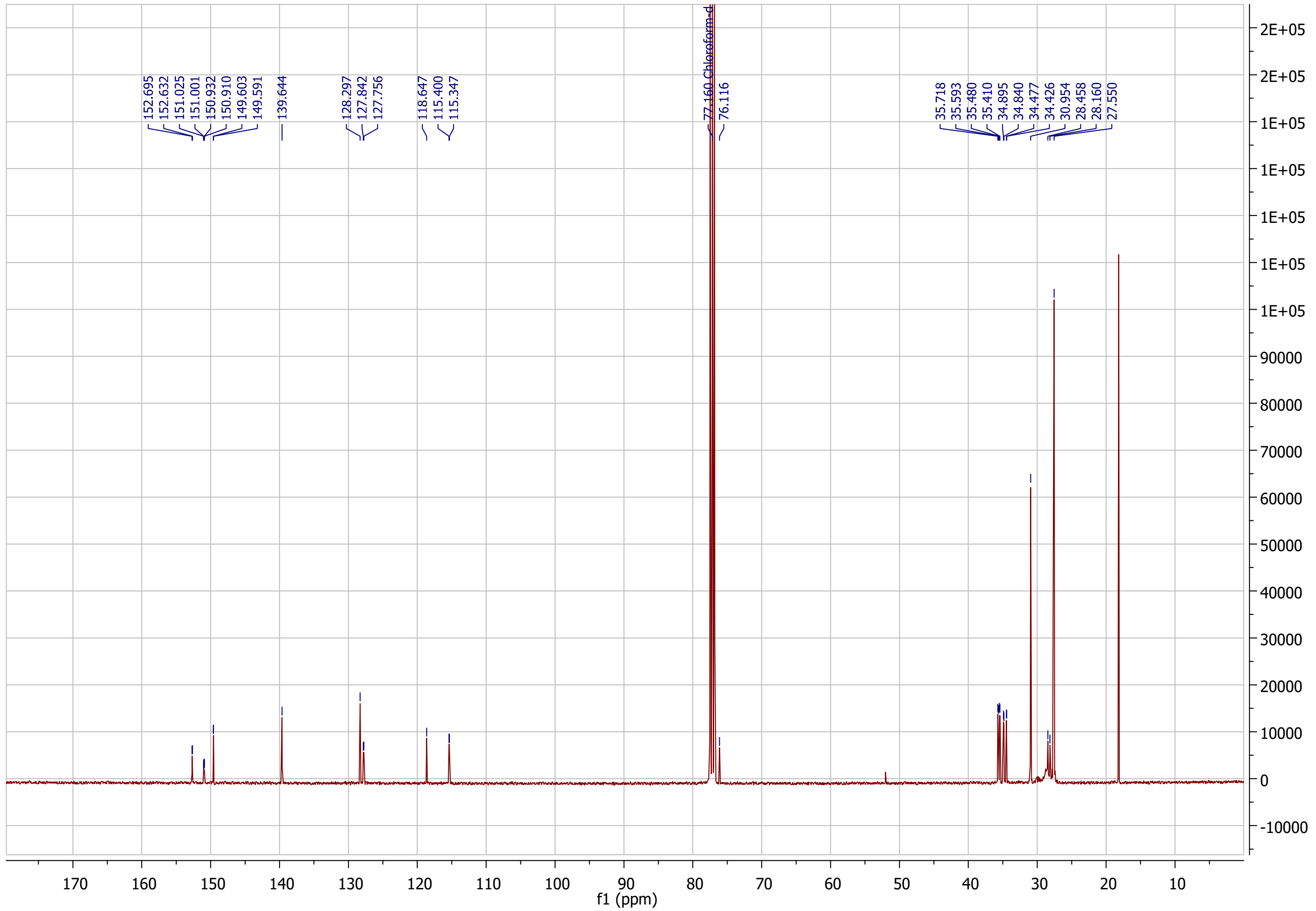

Figure S2. $^{13}C\{^{1}H\}$ NMR spectrum of **2** in $CDCl_3$.

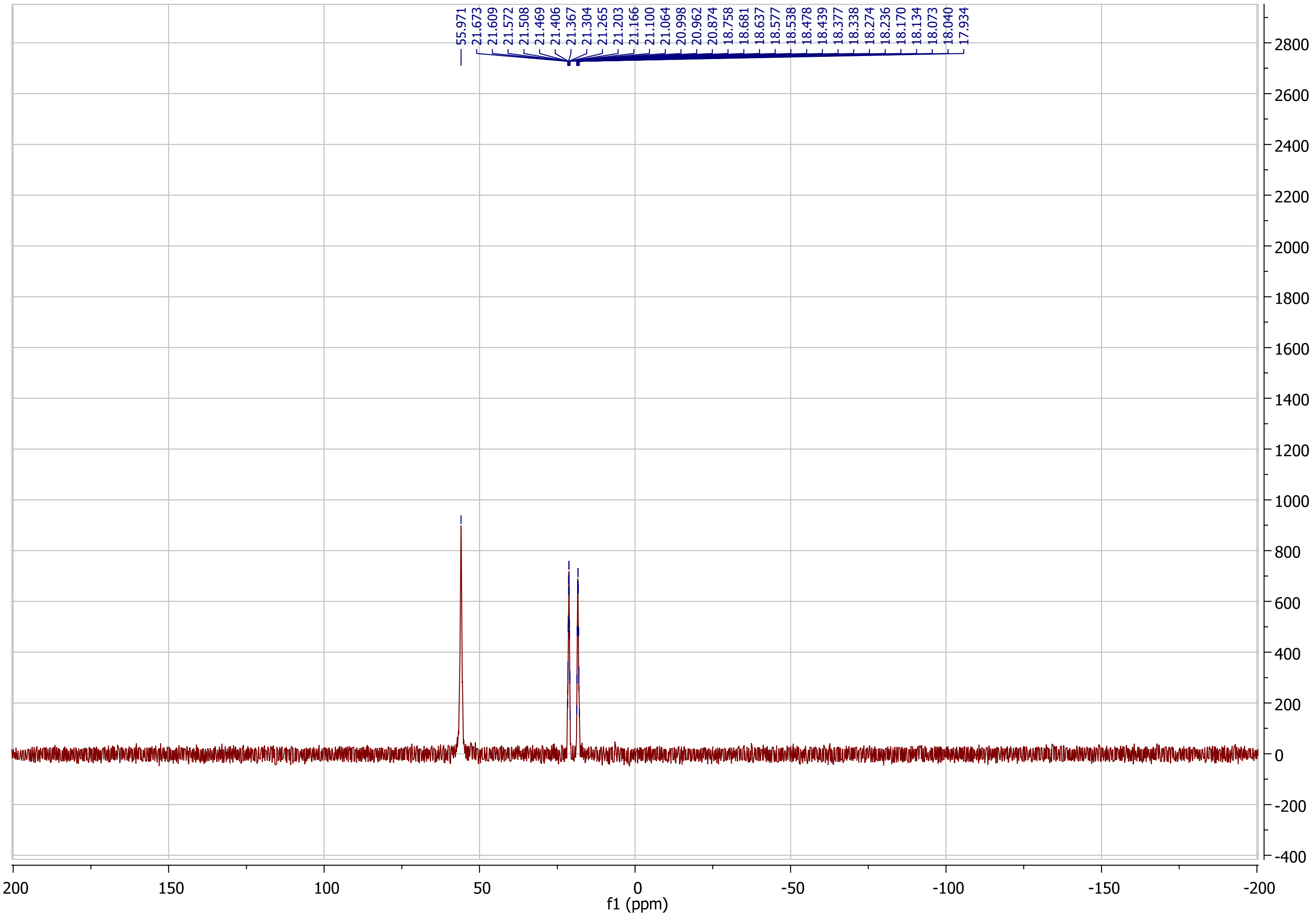

Figure S3. ^{31}P NMR spectrum of **2** in $CDCl_3$.

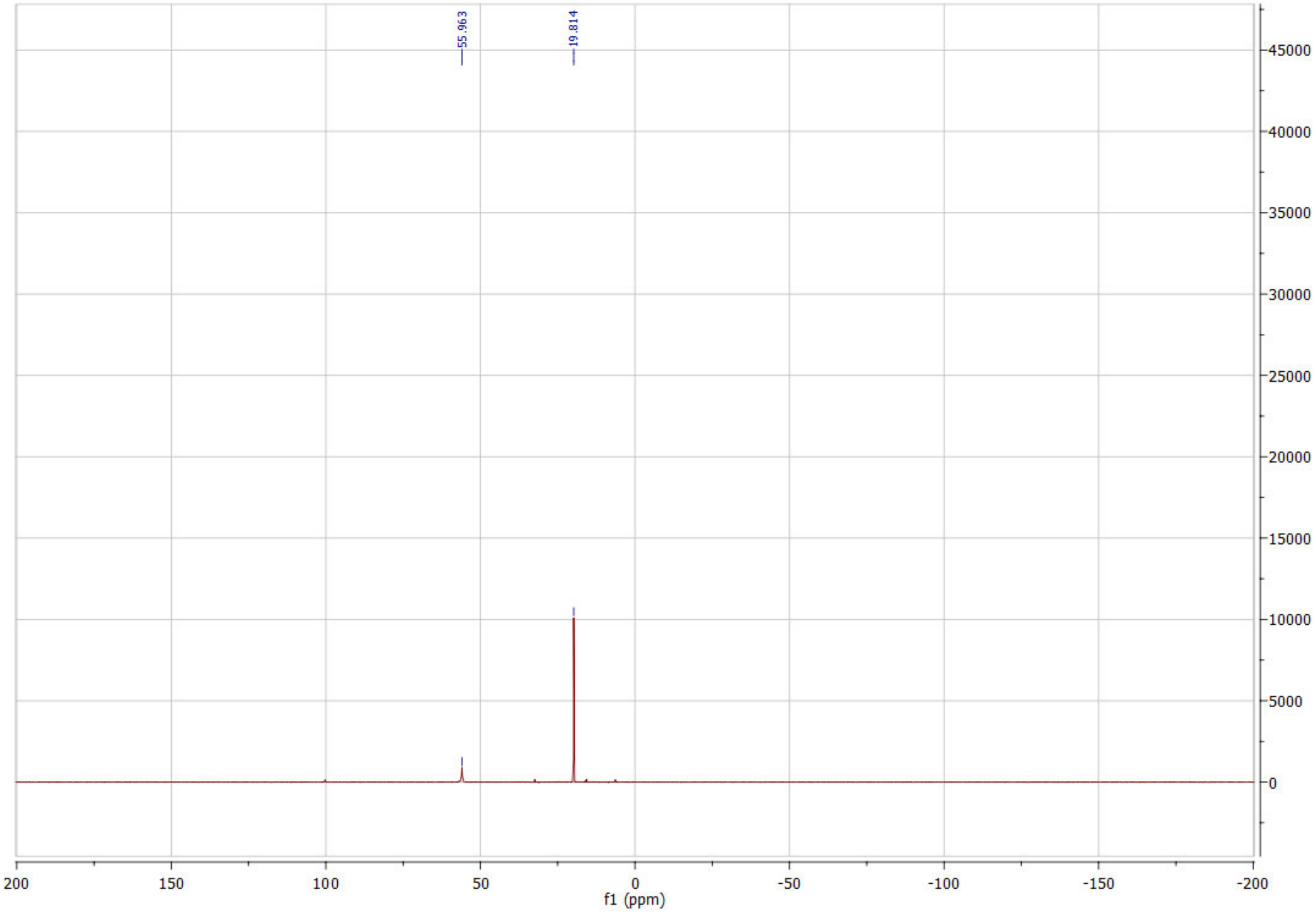

Figure S4. $^{31}P\{^{1}H\}$ NMR spectrum of **2** in $CDCl_3$.

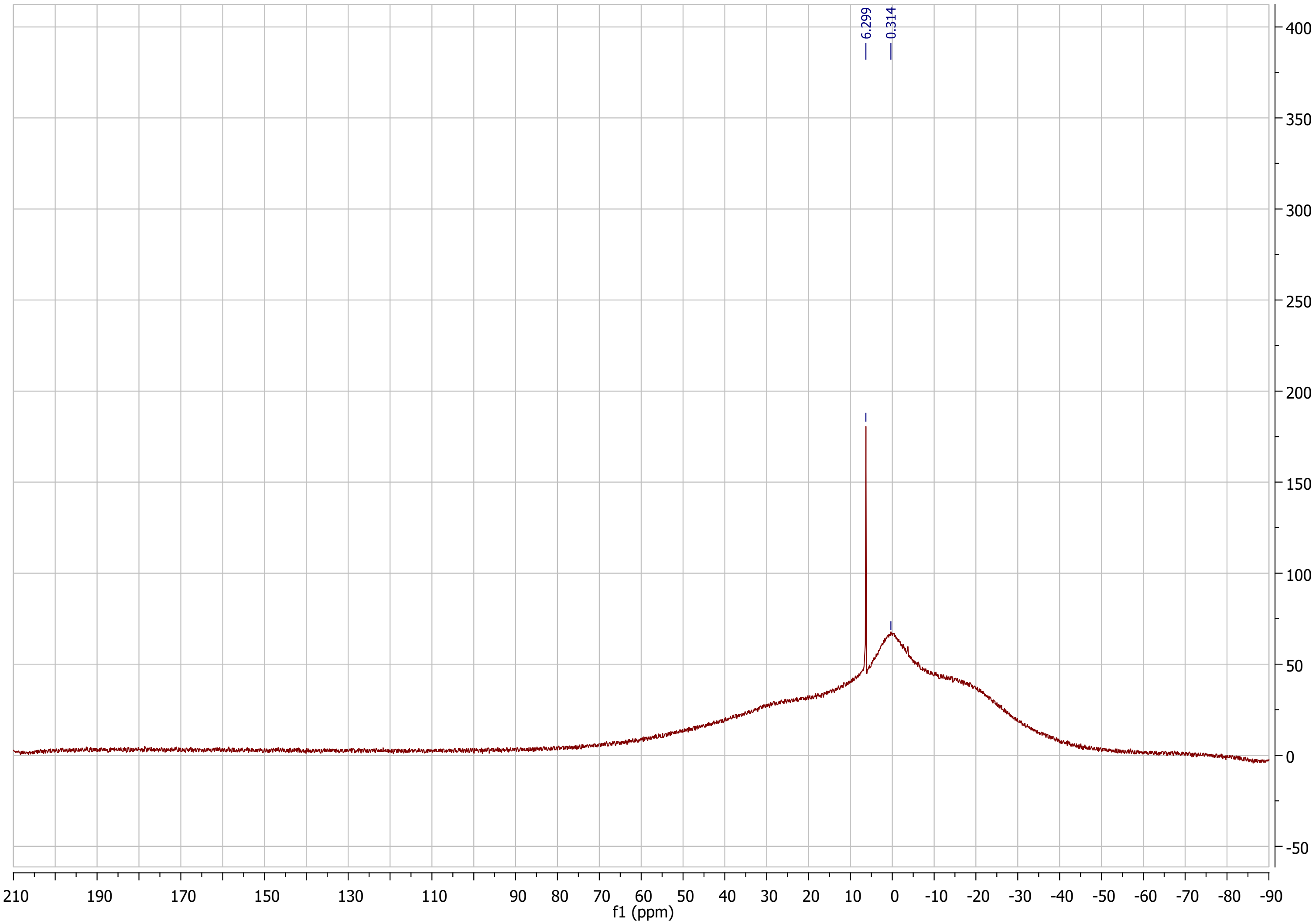

Figure S5. $^{11}B\{^{1}H\}$ NMR spectrum of **2** in $CDCl_3$.

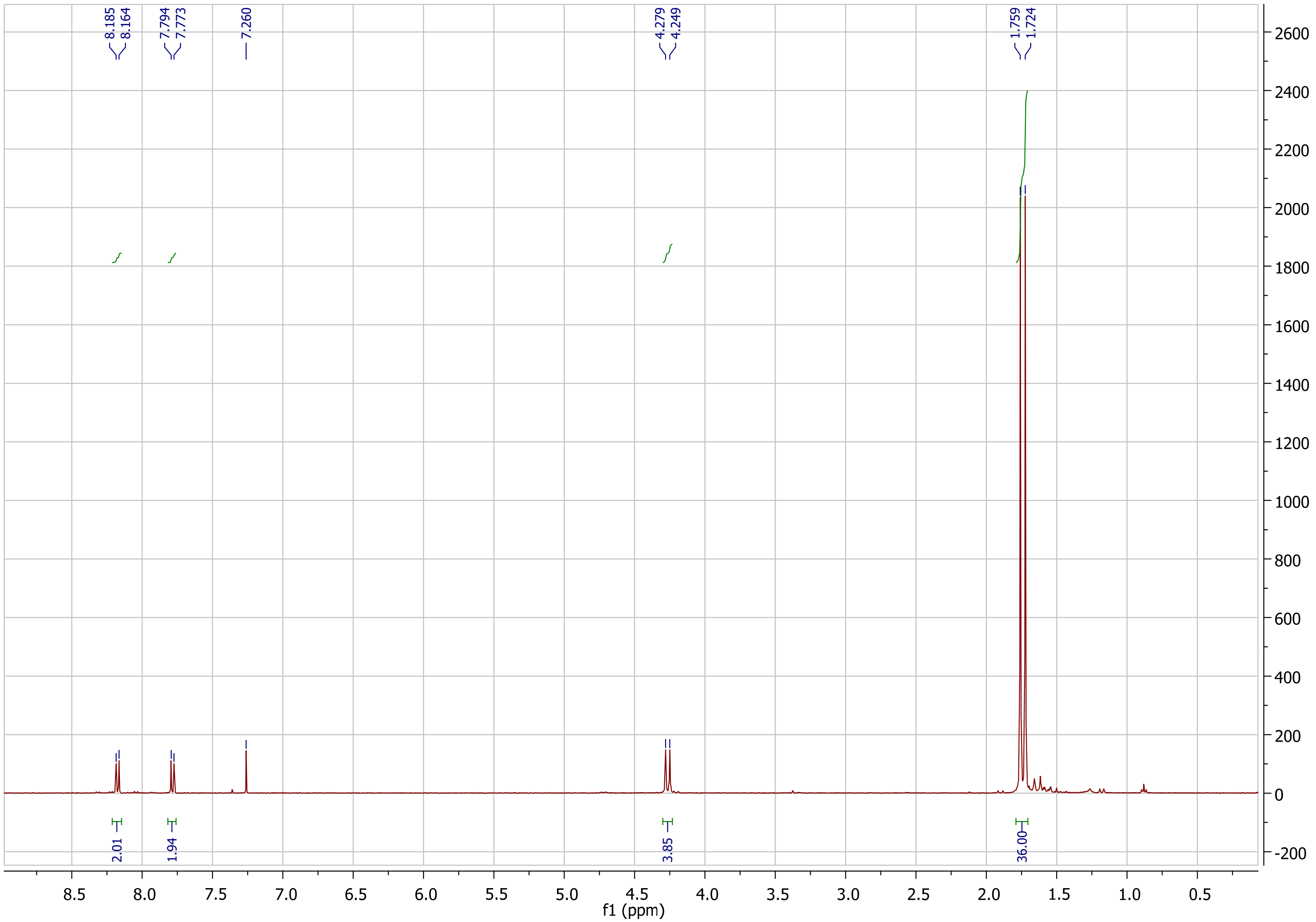

Figure S6. ^{1}H NMR spectrum of **3** in $CDCl_3$.

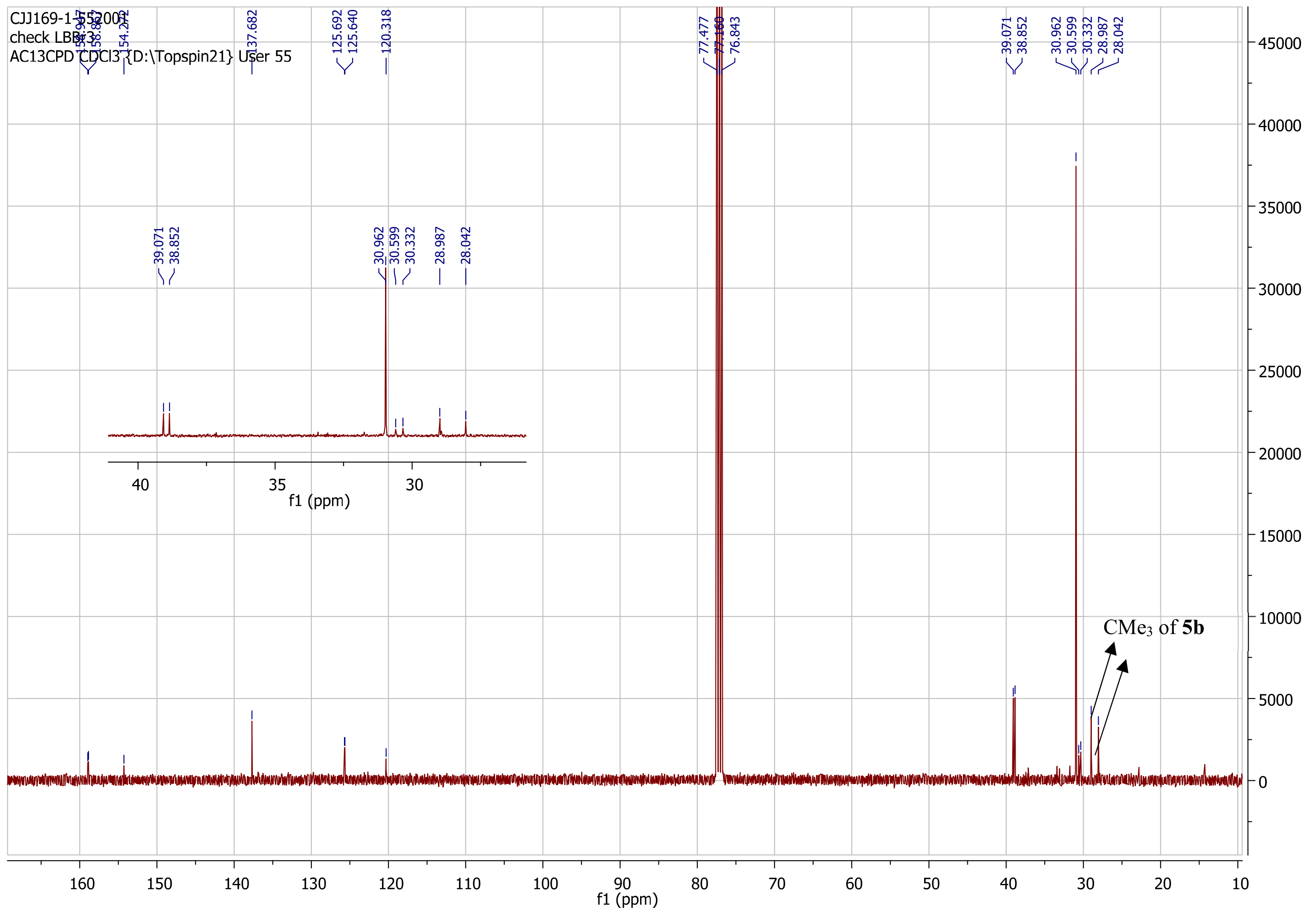

Figure S7. $^{13}C\{^{1}H\}$ NMR spectrum of **3** in $CDCl_3$.

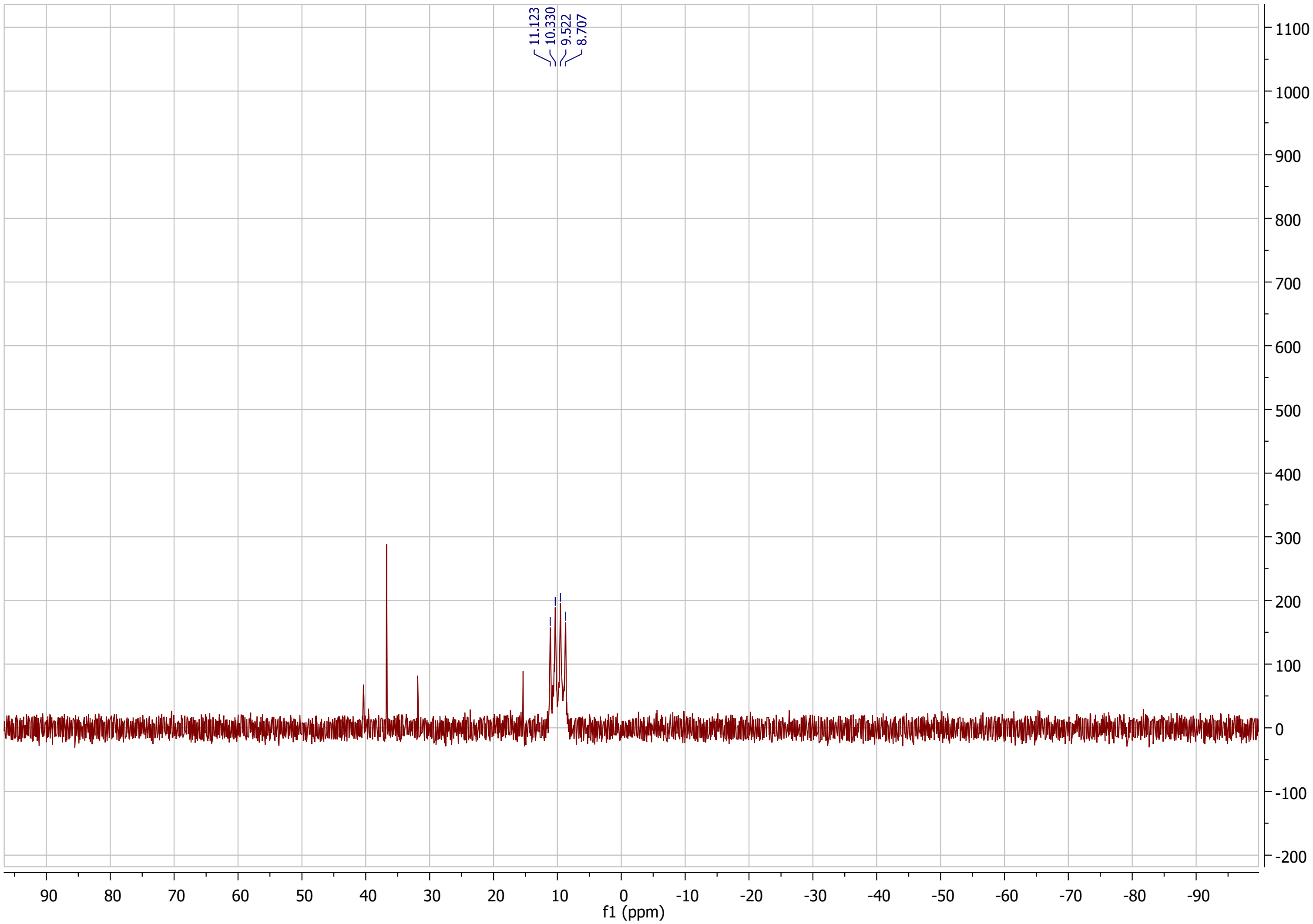

Figure S8. $^{31}P\{^{1}H\}$ NMR spectrum of **3** in $CDCl_3$.

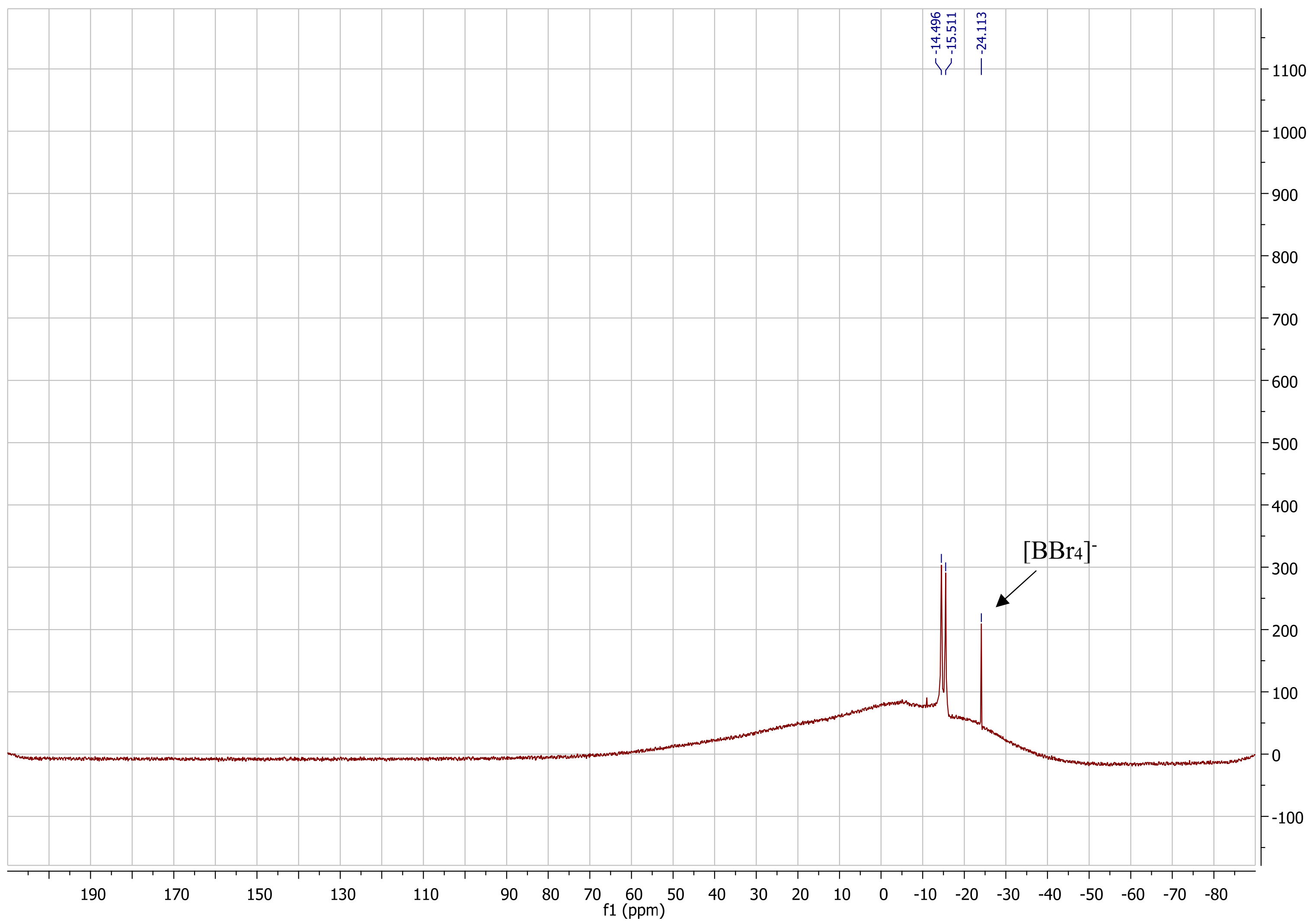

Figure S9. $^{11}B\{^{1}H\}$ NMR spectrum of **3** in $CDCl_3$.

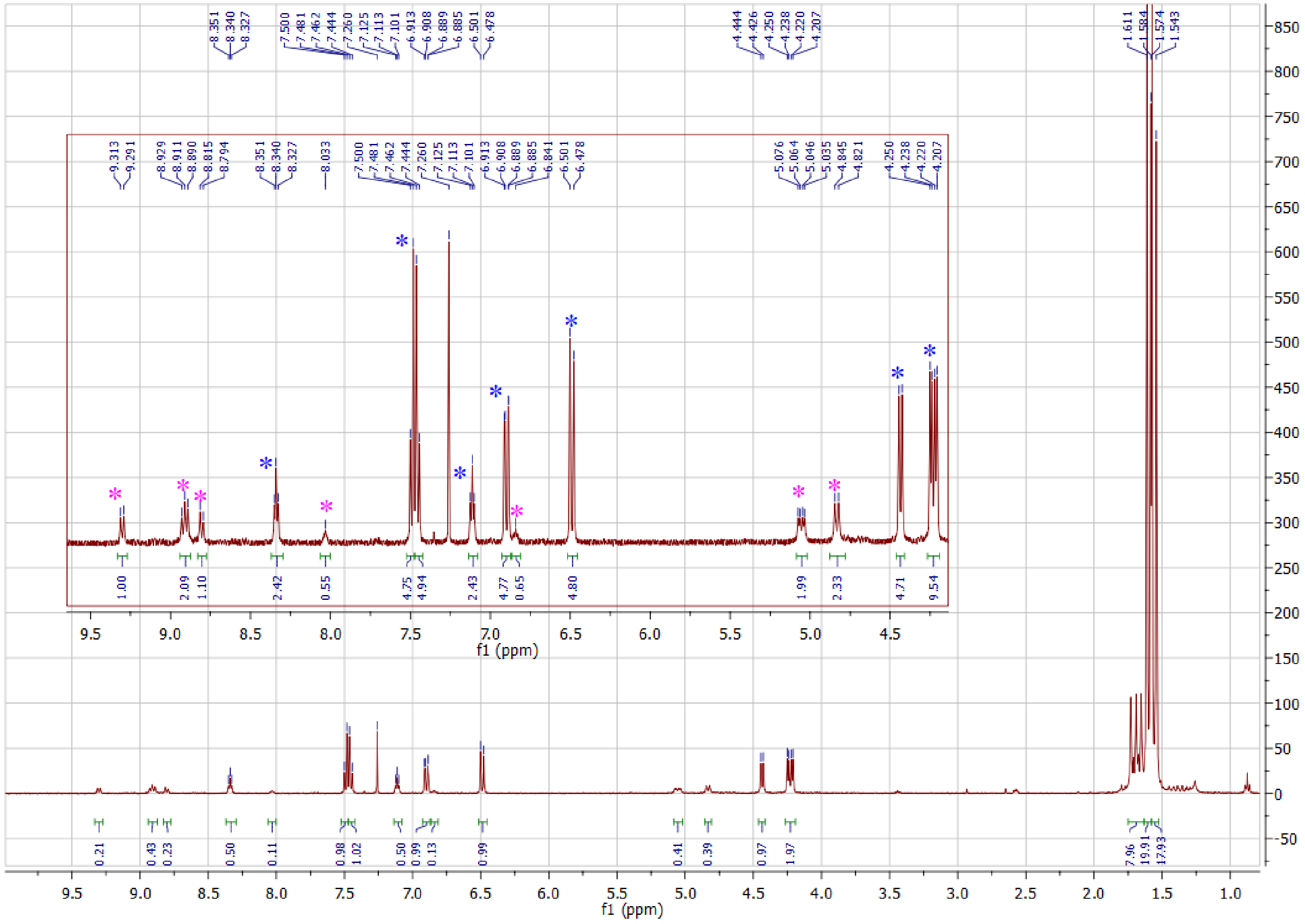

Figure S10. ^1H NMR spectrum of **4a** in $CDCl_3$ (The blue and pink stars represent **4a** and **[LH]$^+$**, respectively).

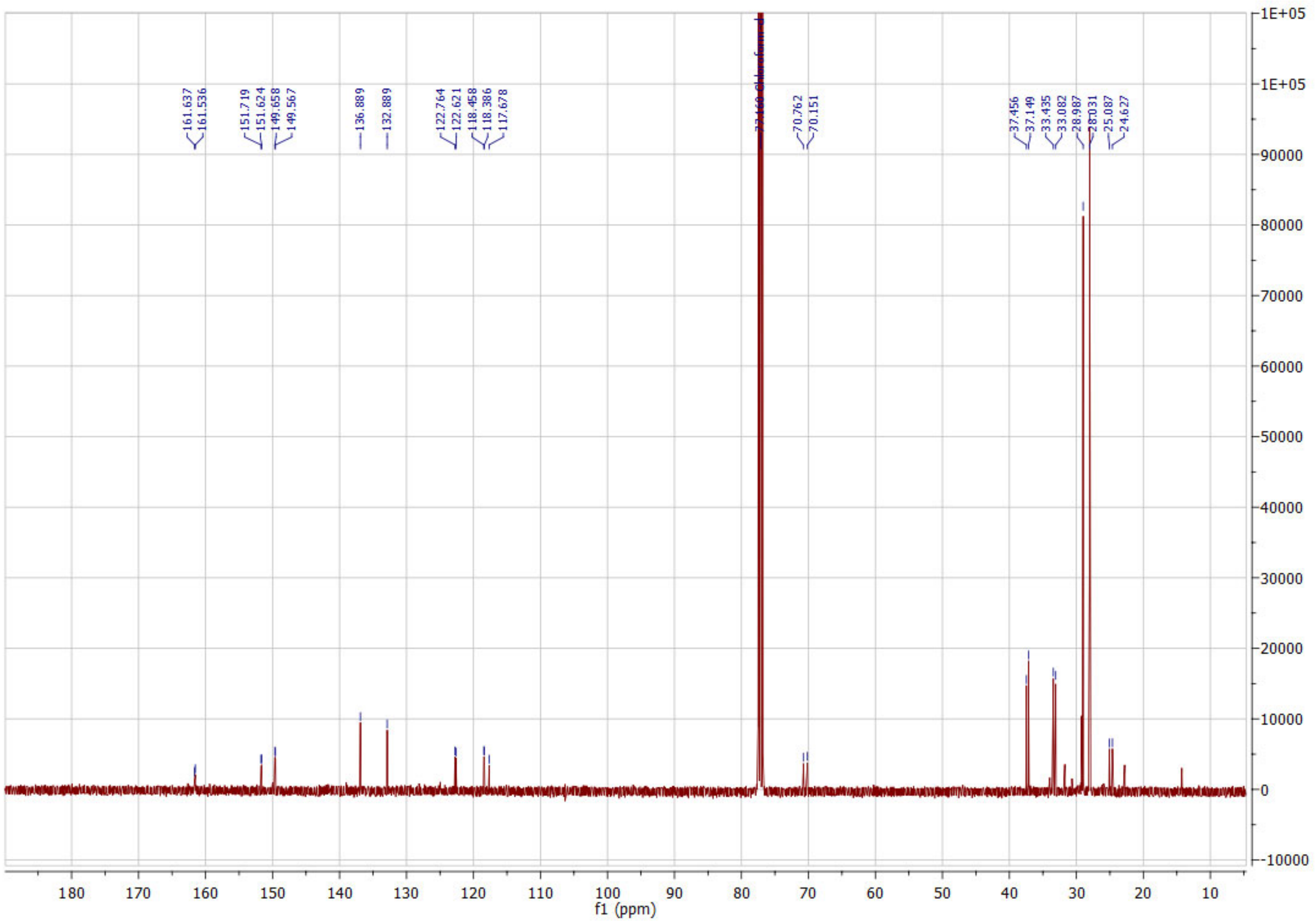

Figure S11. $^{13}C\{^{1}H\}$ NMR spectrum of **4a** in $CDCl_3$.

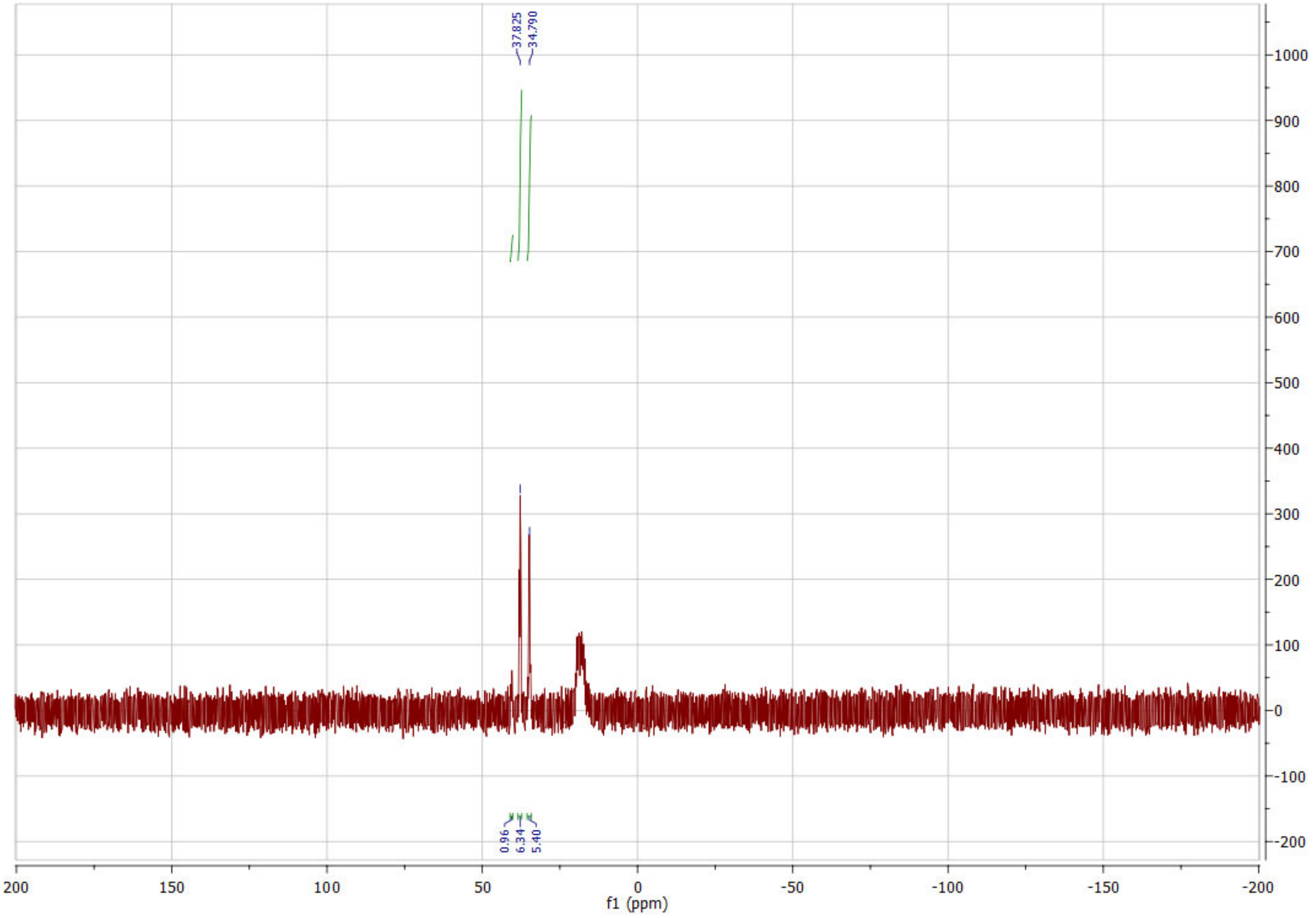

Figure S12. ^{31}P NMR spectrum of **4a** in $CDCl_3$.

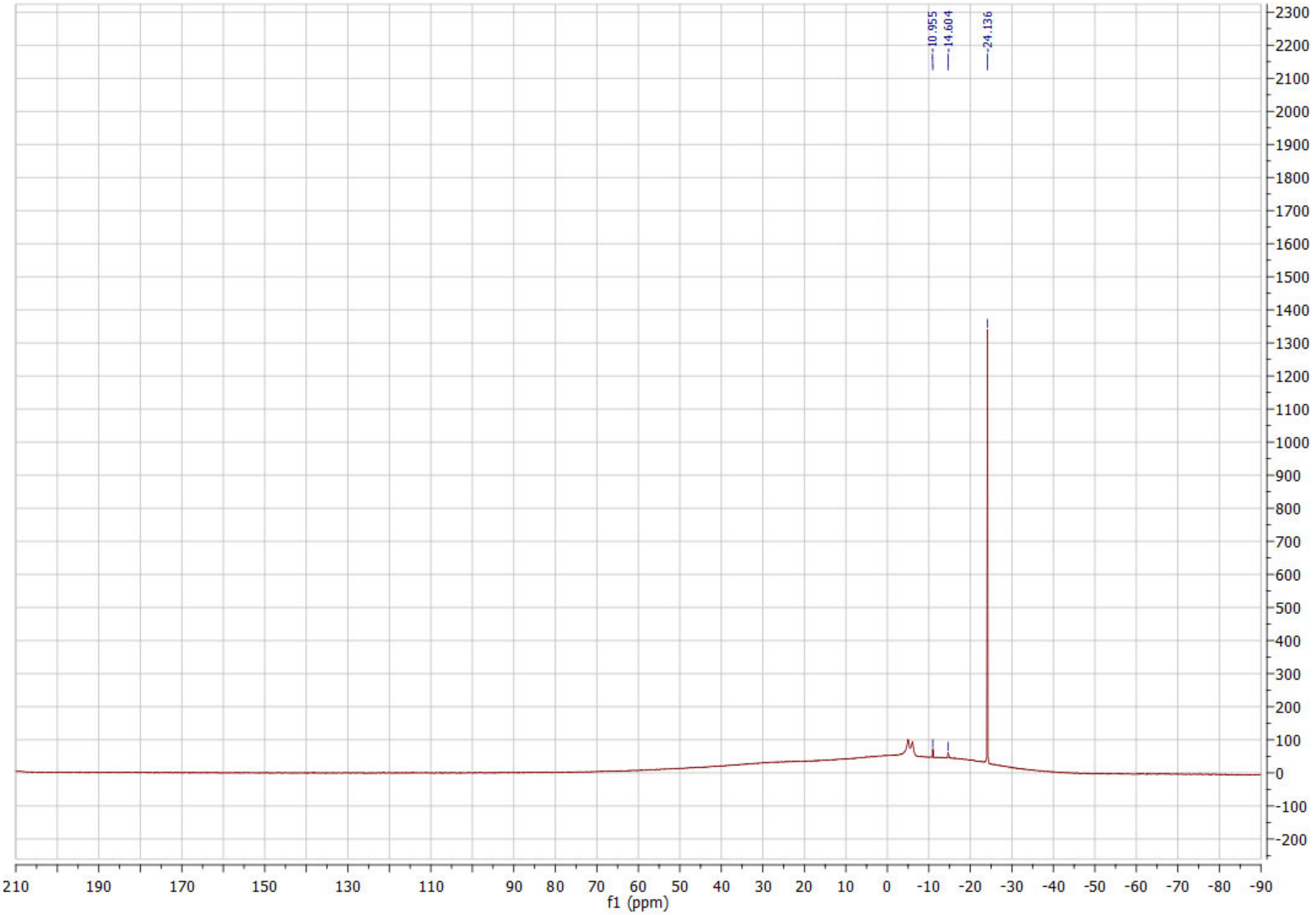

Figure S13. $^{11}B\{^{1}H\}$ NMR spectrum of **4a** in $CDCl_3$.

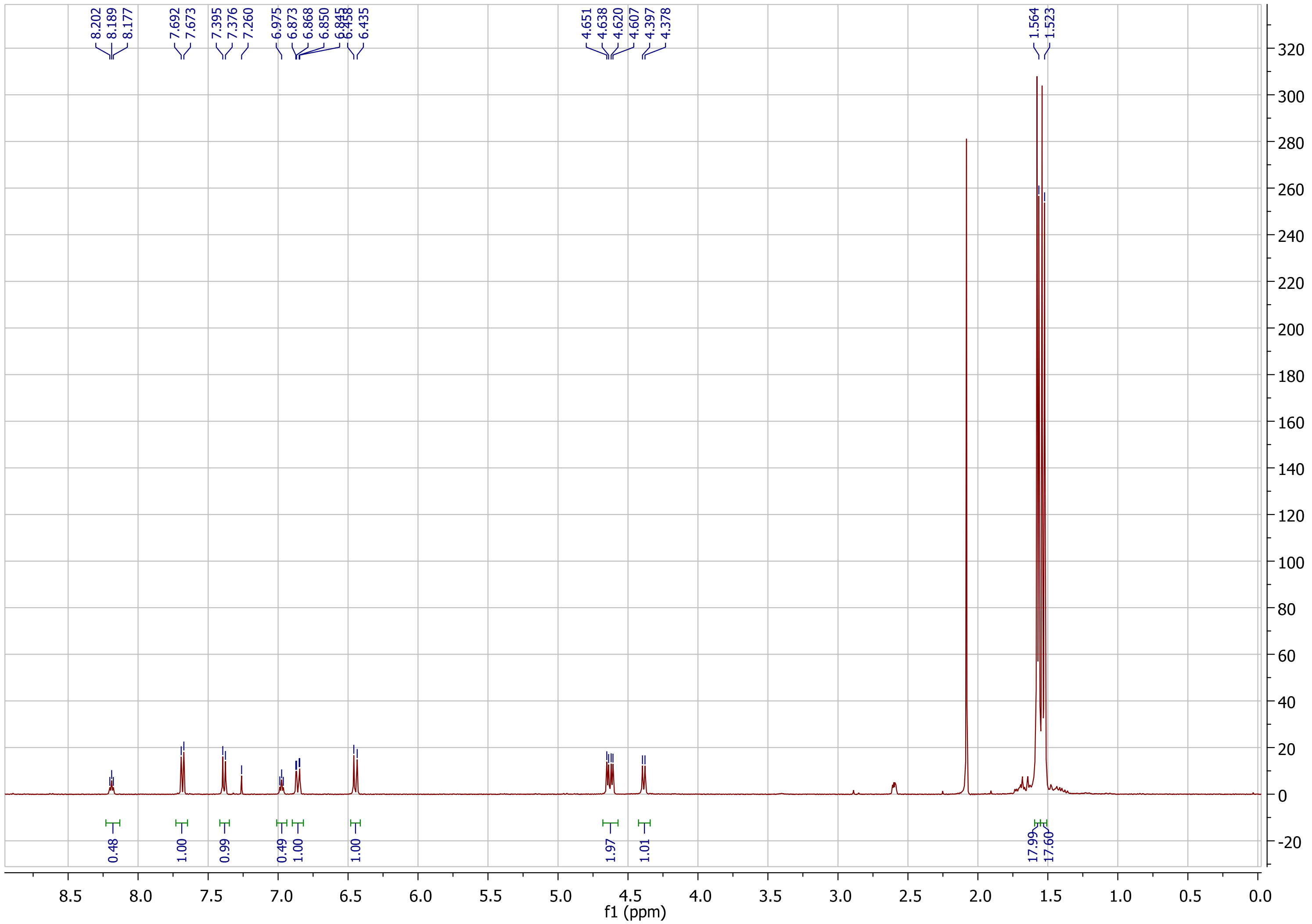

Figure S14. ¹H NMR spectrum of **4b** in $CDCl_3$.

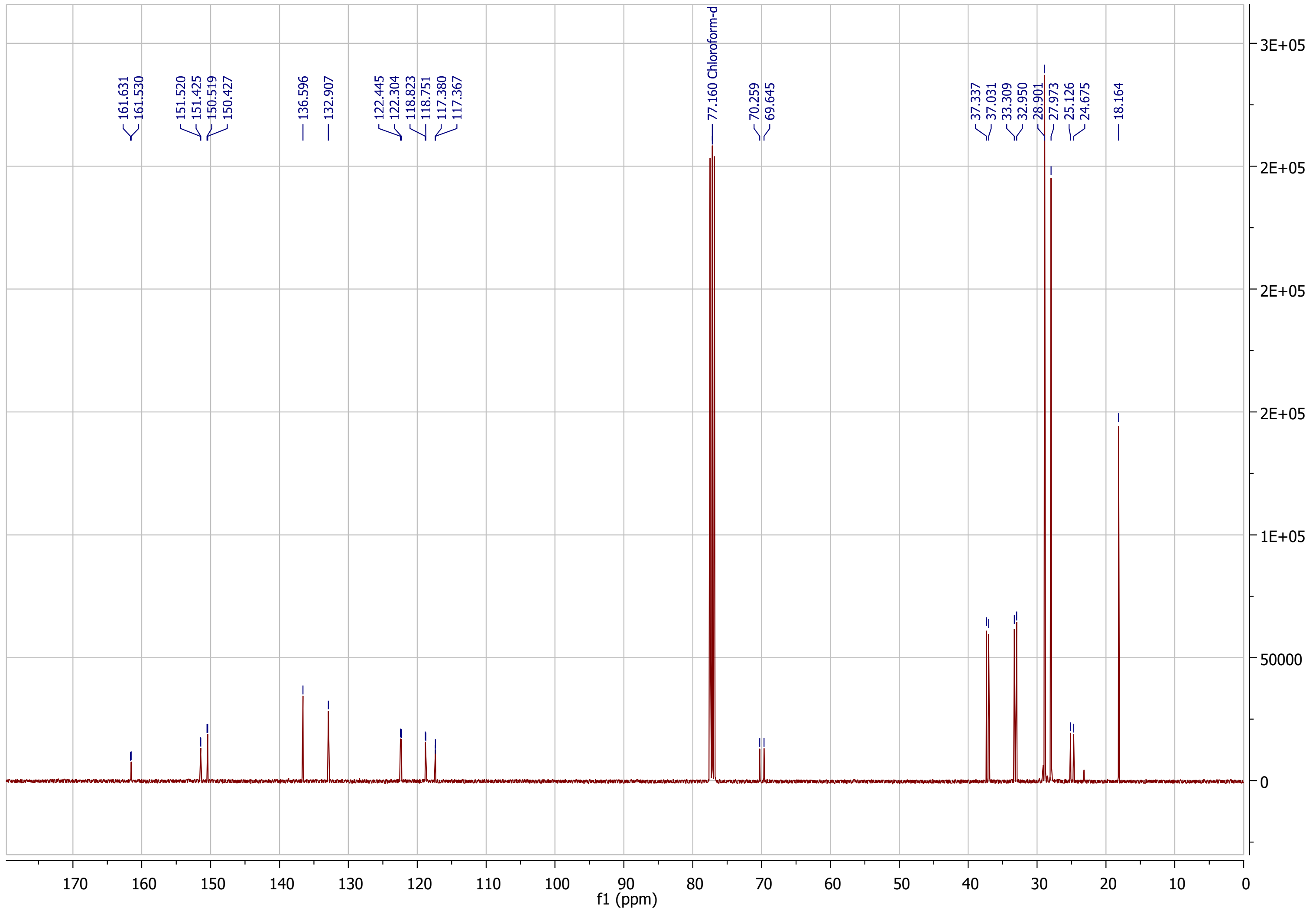

Figure S15. $^{13}C\{^{1}H\}$ NMR spectrum of **4b** in $CDCl_3$.

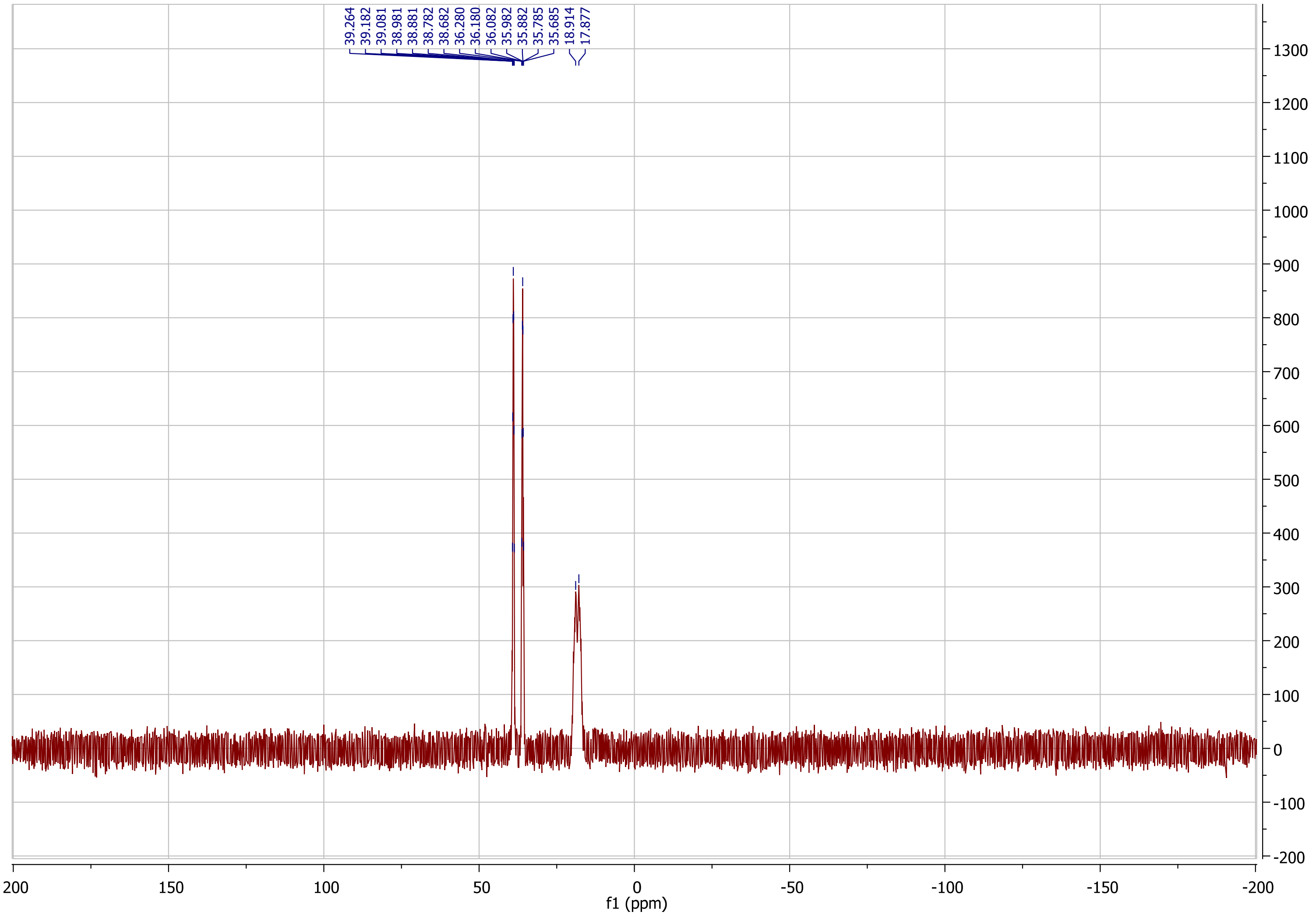

Figure S16. $^{31}P\{^{1}H\}$ NMR spectrum of **4b** in $CDCl_3$.

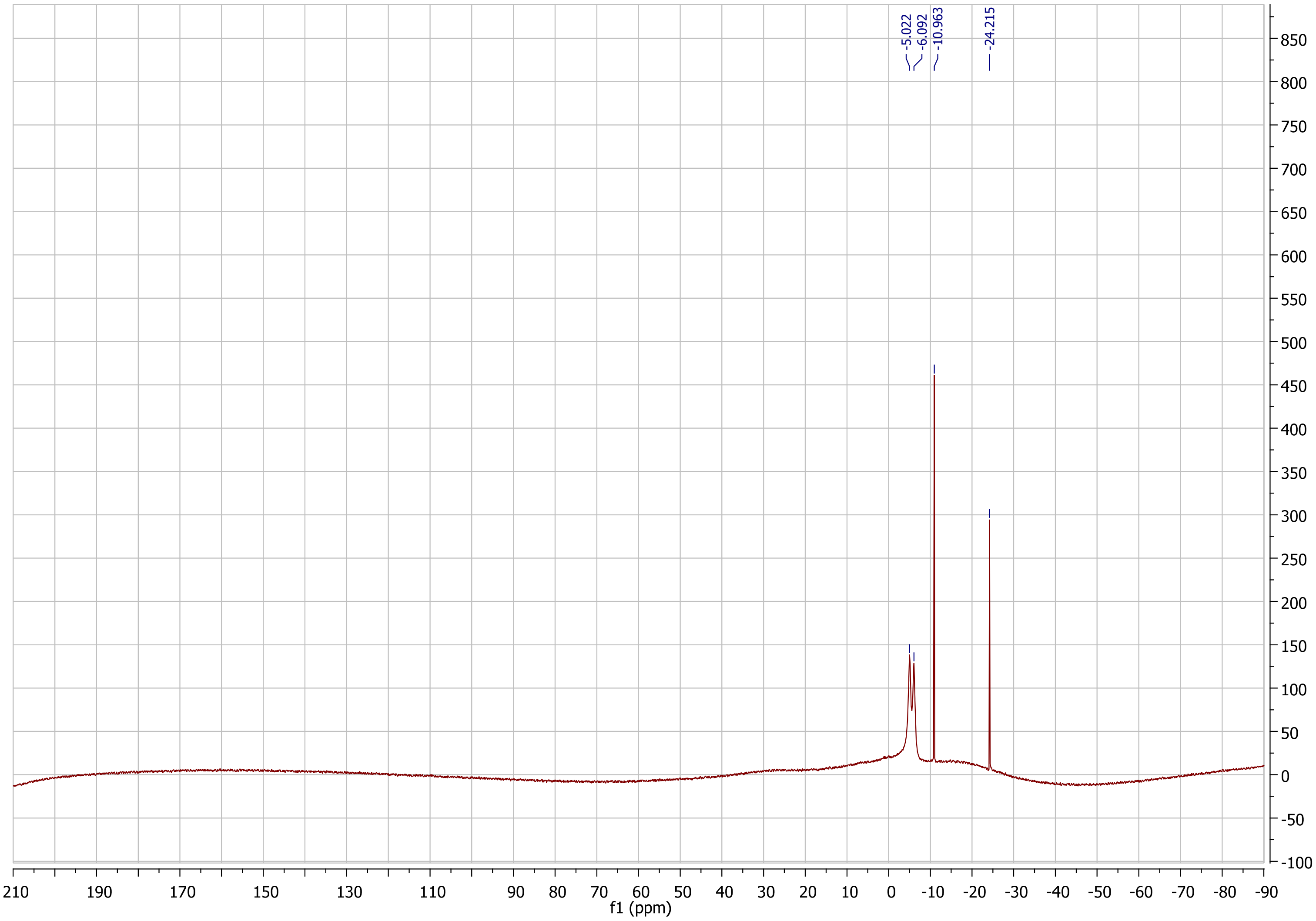

Figure S17. $^{11}B\{^{1}H\}$ NMR spectrum of **4b** in $CDCl_3$.

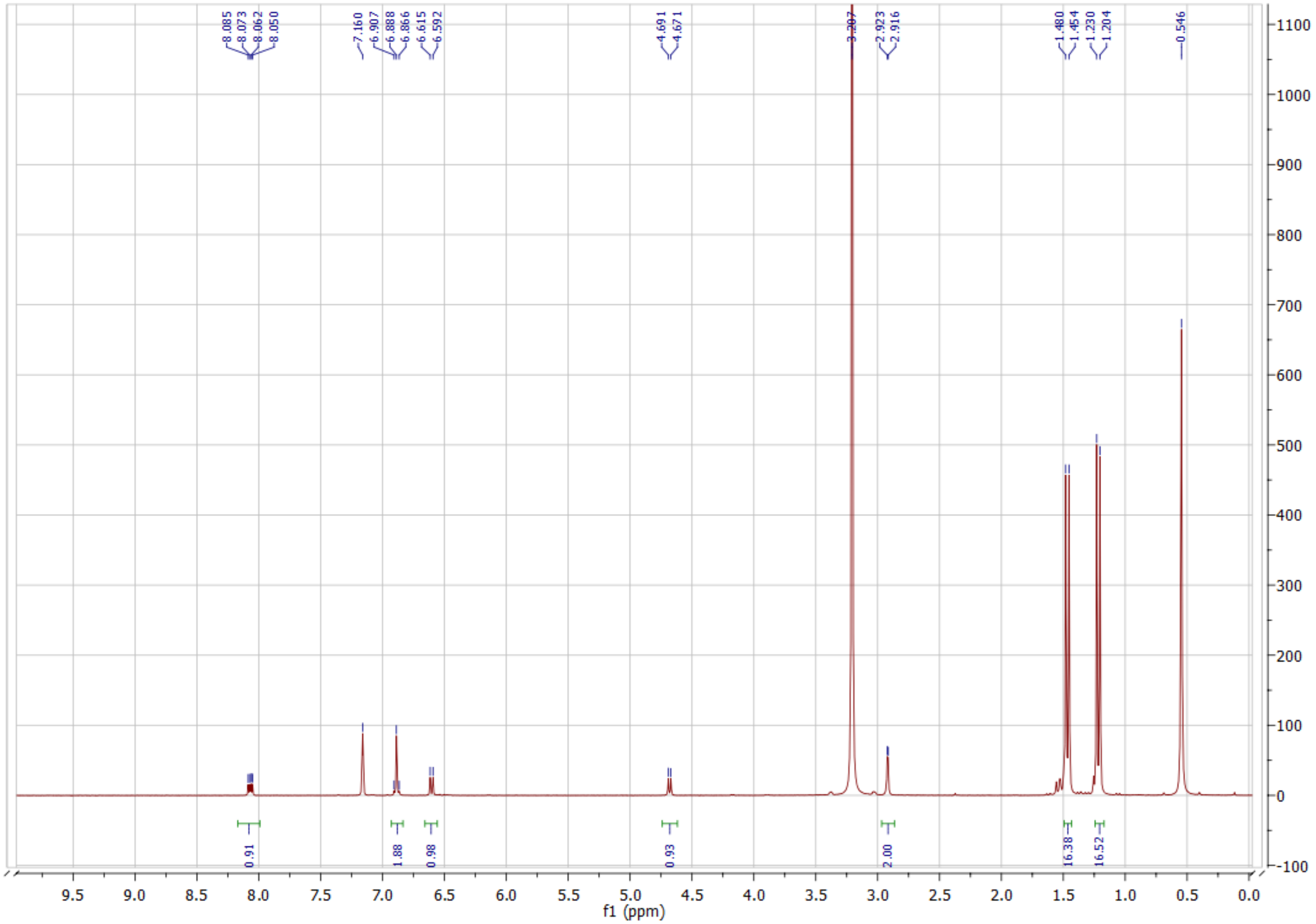

Figure S18. ^{1}H NMR spectrum of **5-18C6** in C_6D_6.

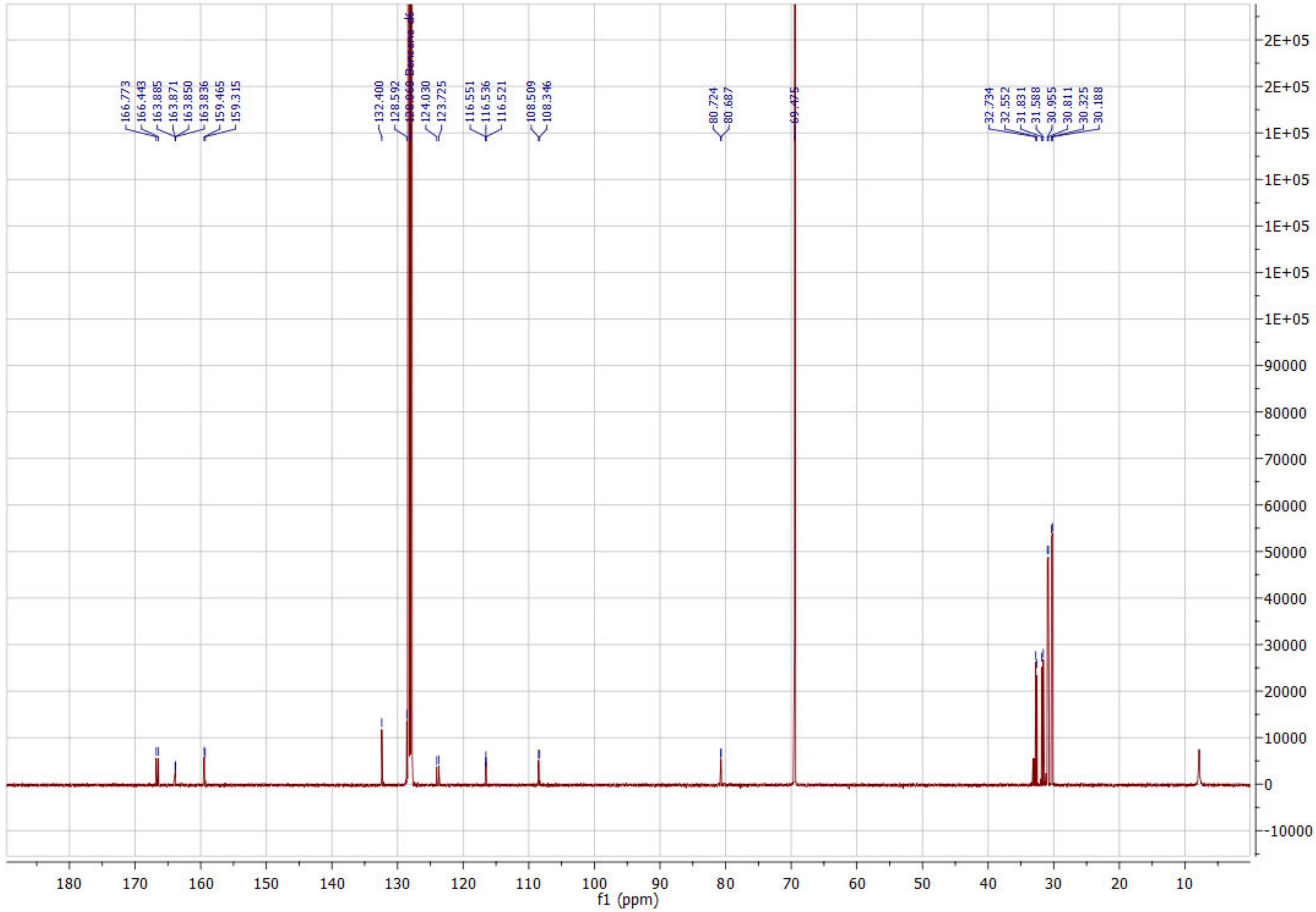

Figure S19. $^{13}C\{^{1}H\}$ NMR spectrum of **5-18C6** in C_6D_6.

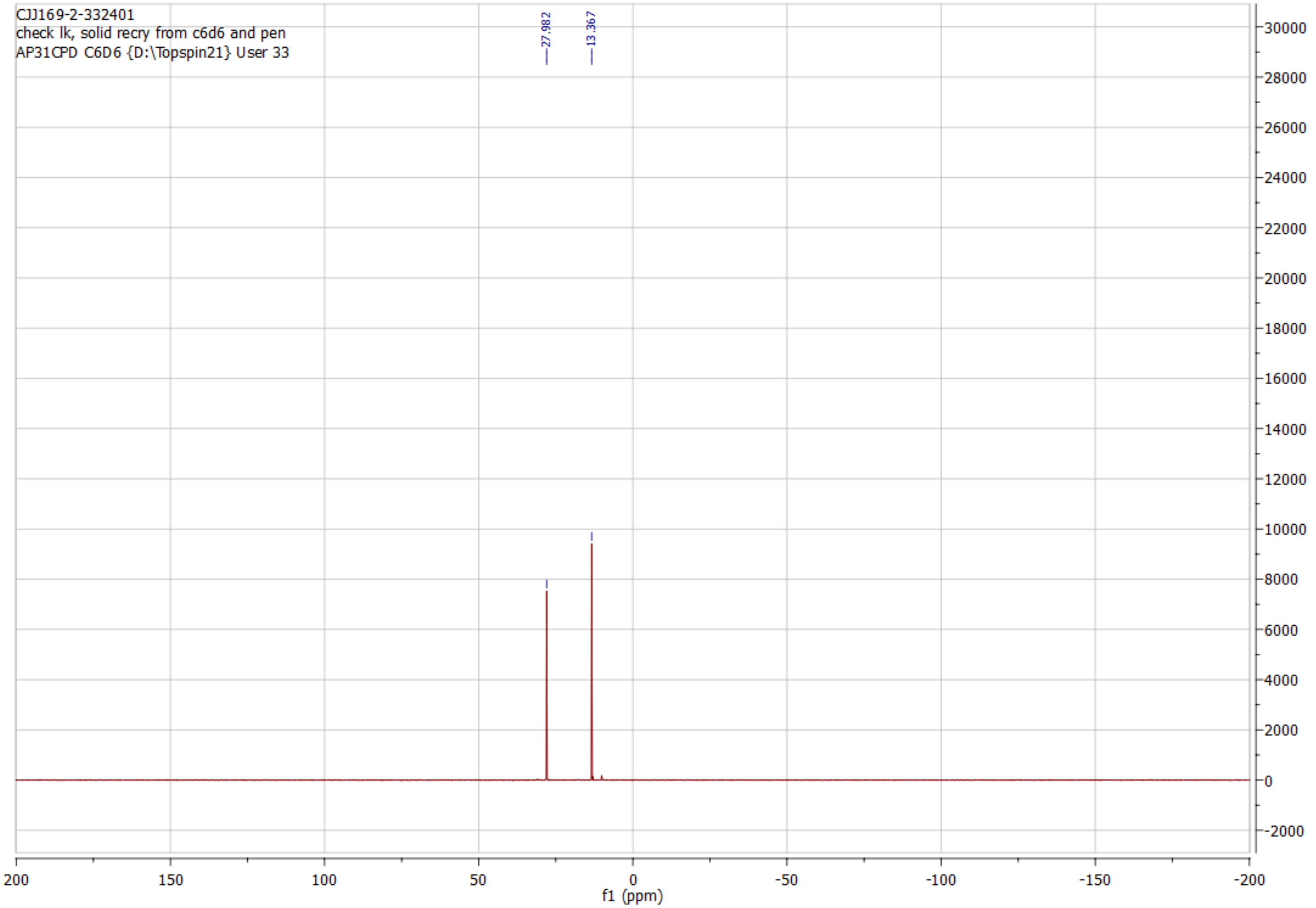

Figure S20. $^{31}P\{^{1}H\}$ NMR spectrum of **5-18C6** in C_6D_6.

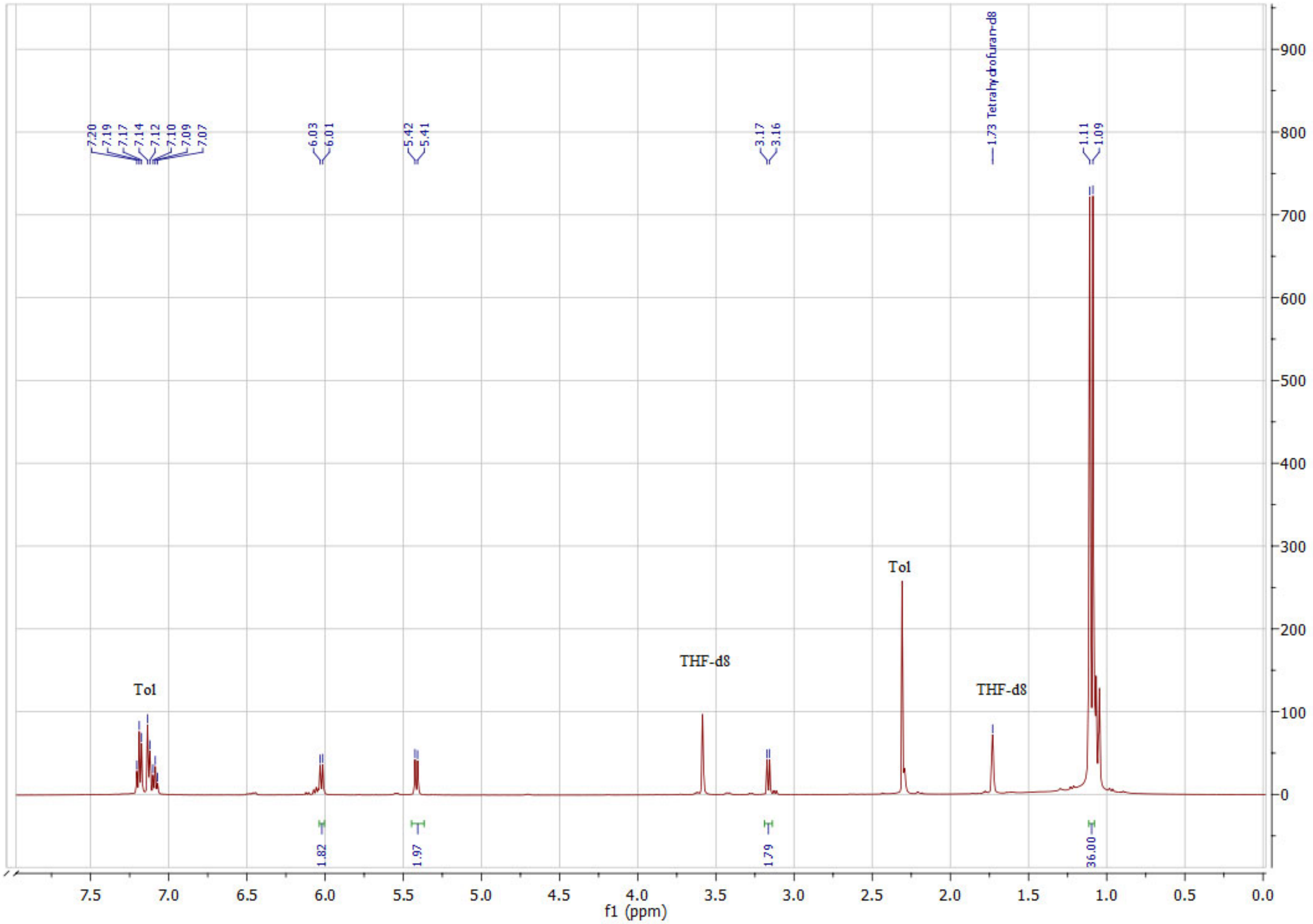

Figure S21. ^{1}H NMR spectrum of **6** in THF-d_8.

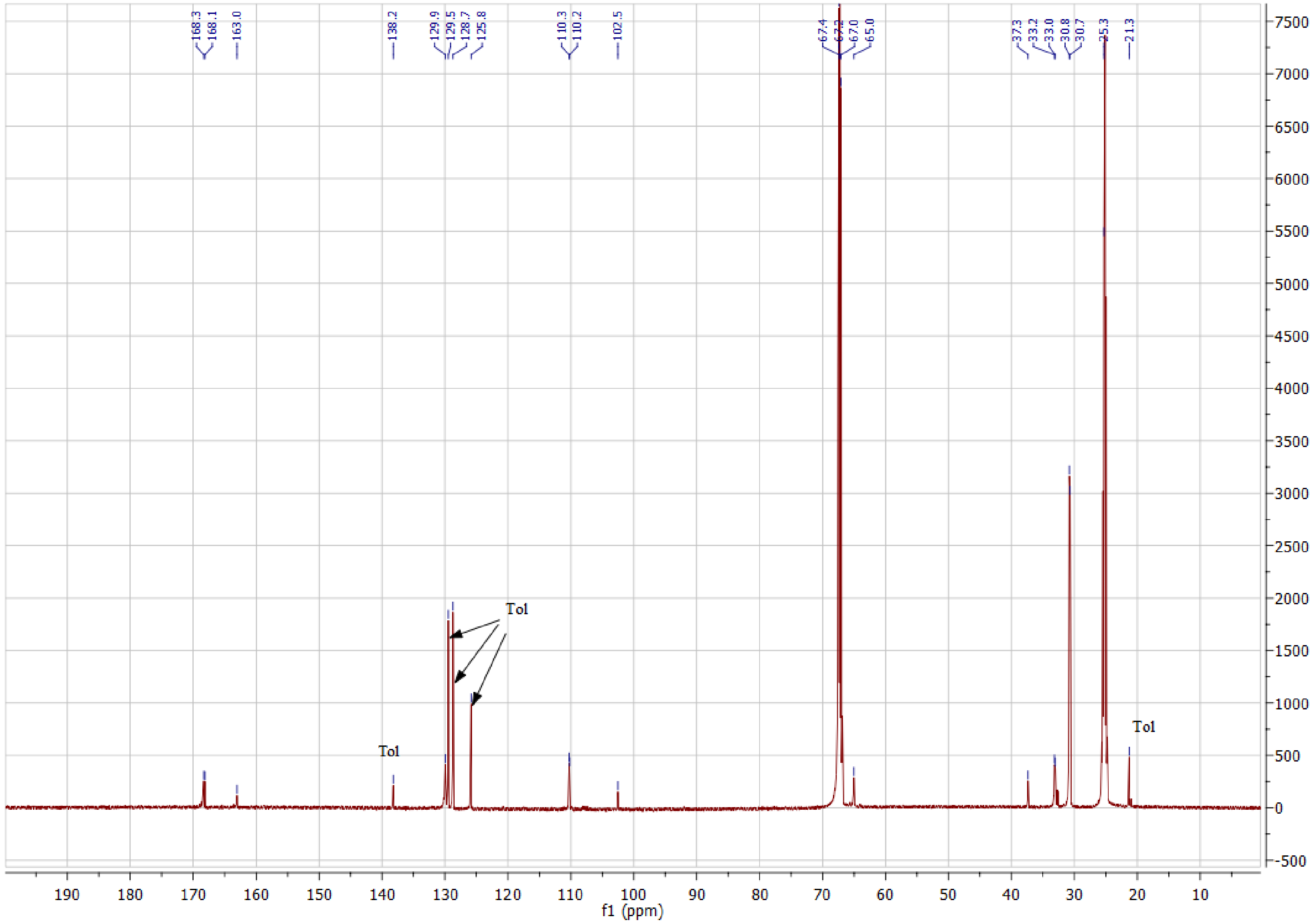

Figure S22. $^{13}C\{^{1}H\}$ NMR spectrum of **6** in THF-d_8.

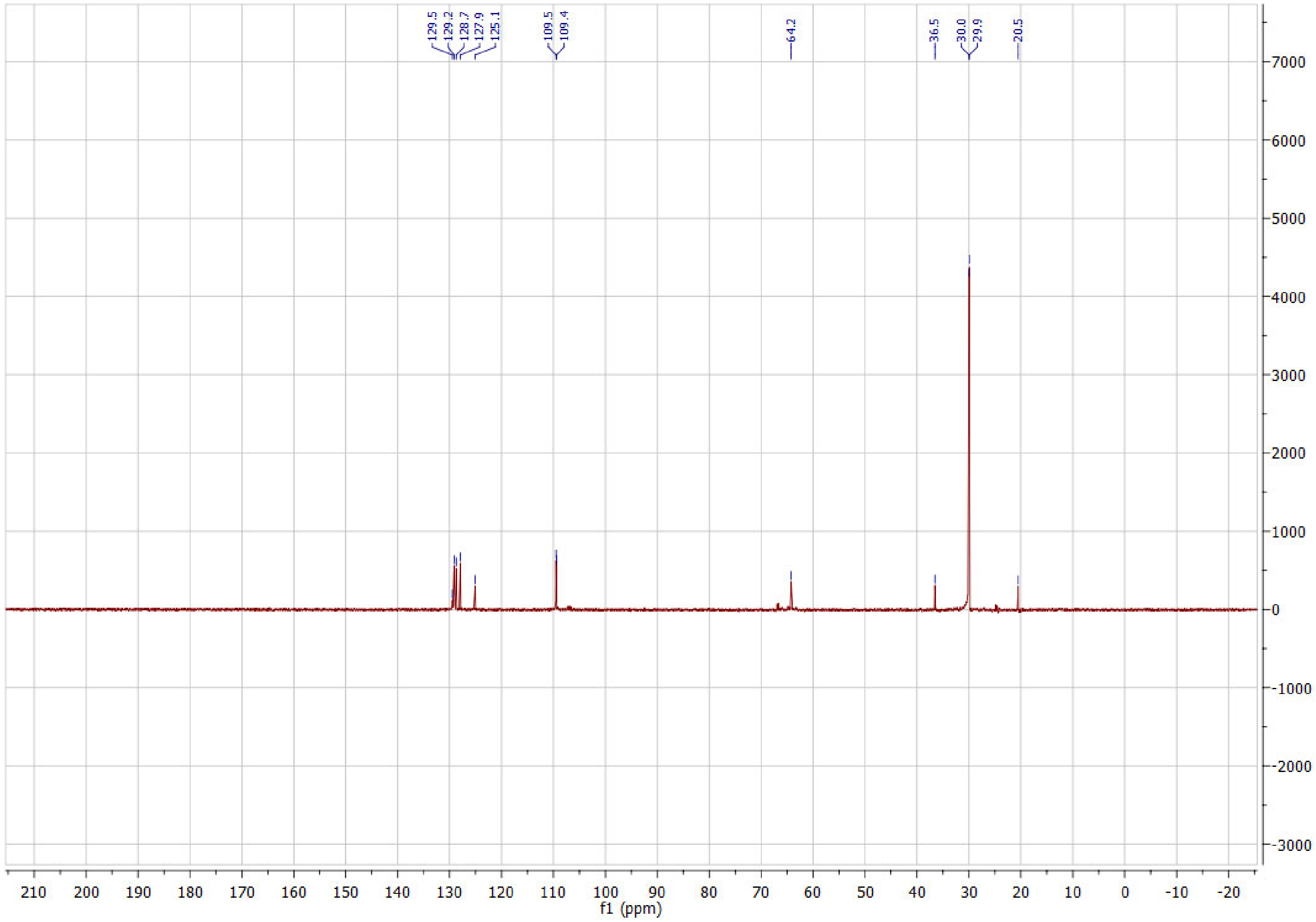

Figure S23. DEPT135 NMR spectrum of **6** in THF-d_8.

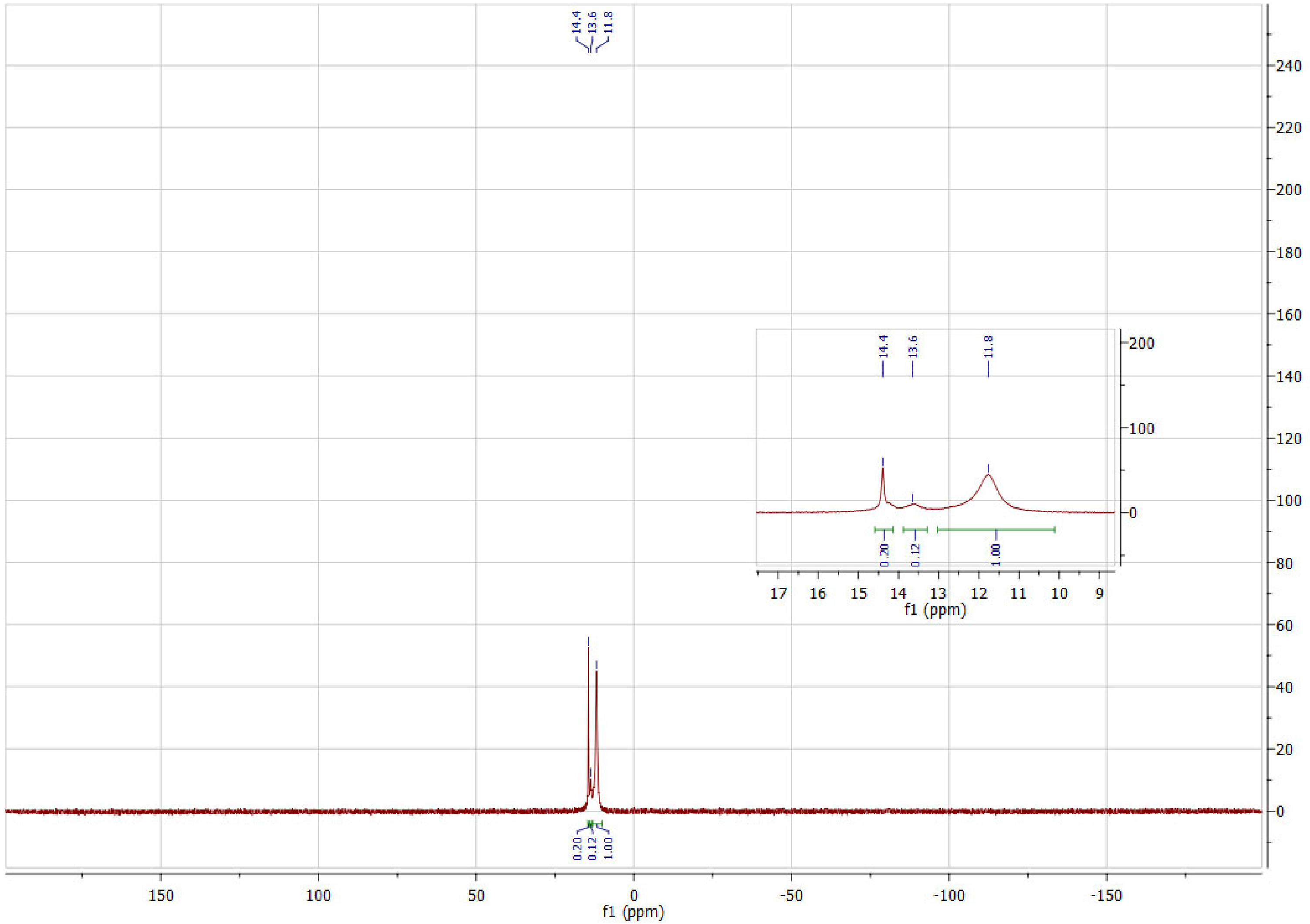

Figure S24. $^{31}P\{^{1}H\}$ NMR spectrum of **6** in THF-d_8.

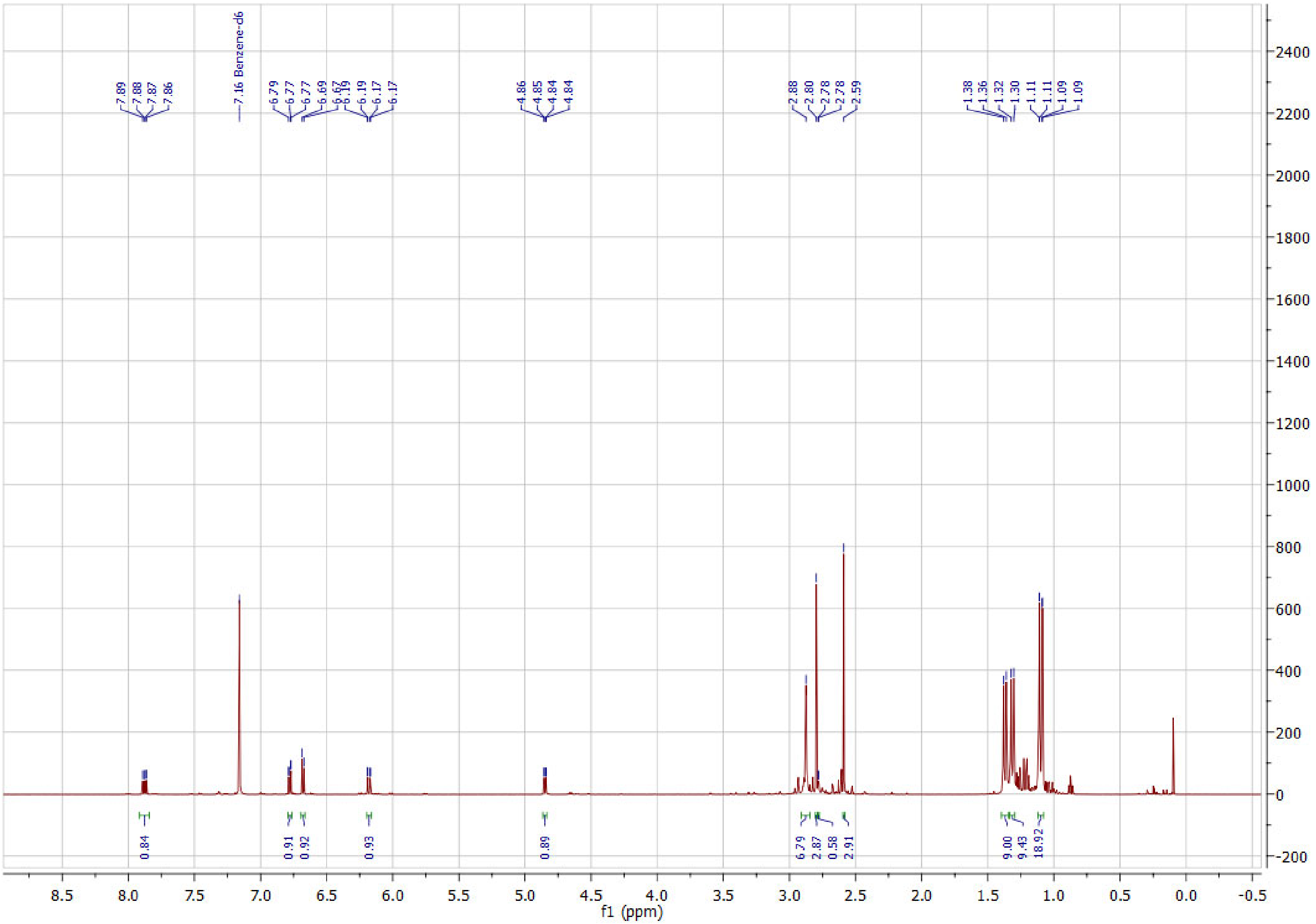

Figure S25. ^{1}H NMR spectrum of **7** in C_6D_6.

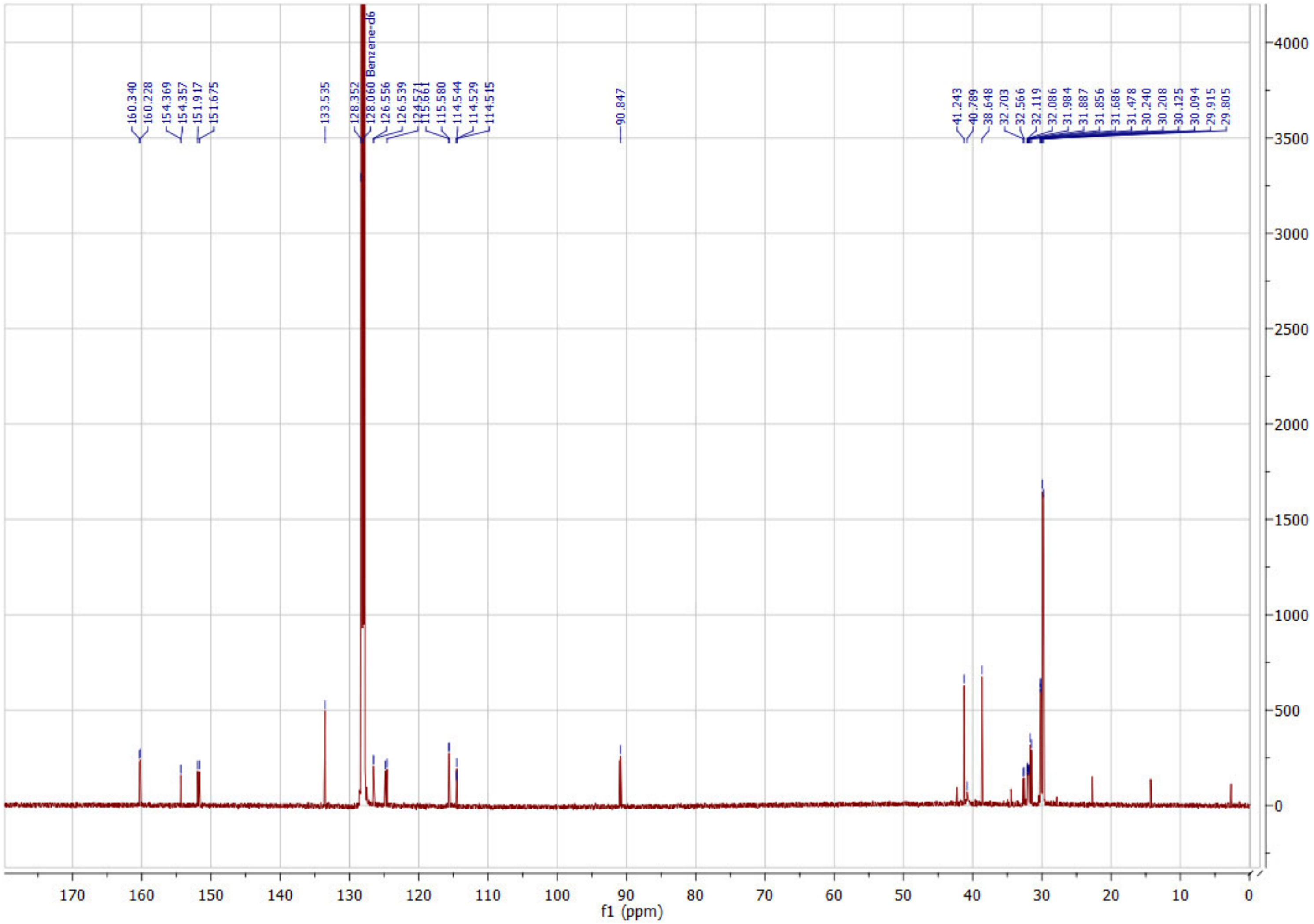

Figure S26. $^{13}C\{^1H\}$ NMR spectrum of **7** in C_6D_6.

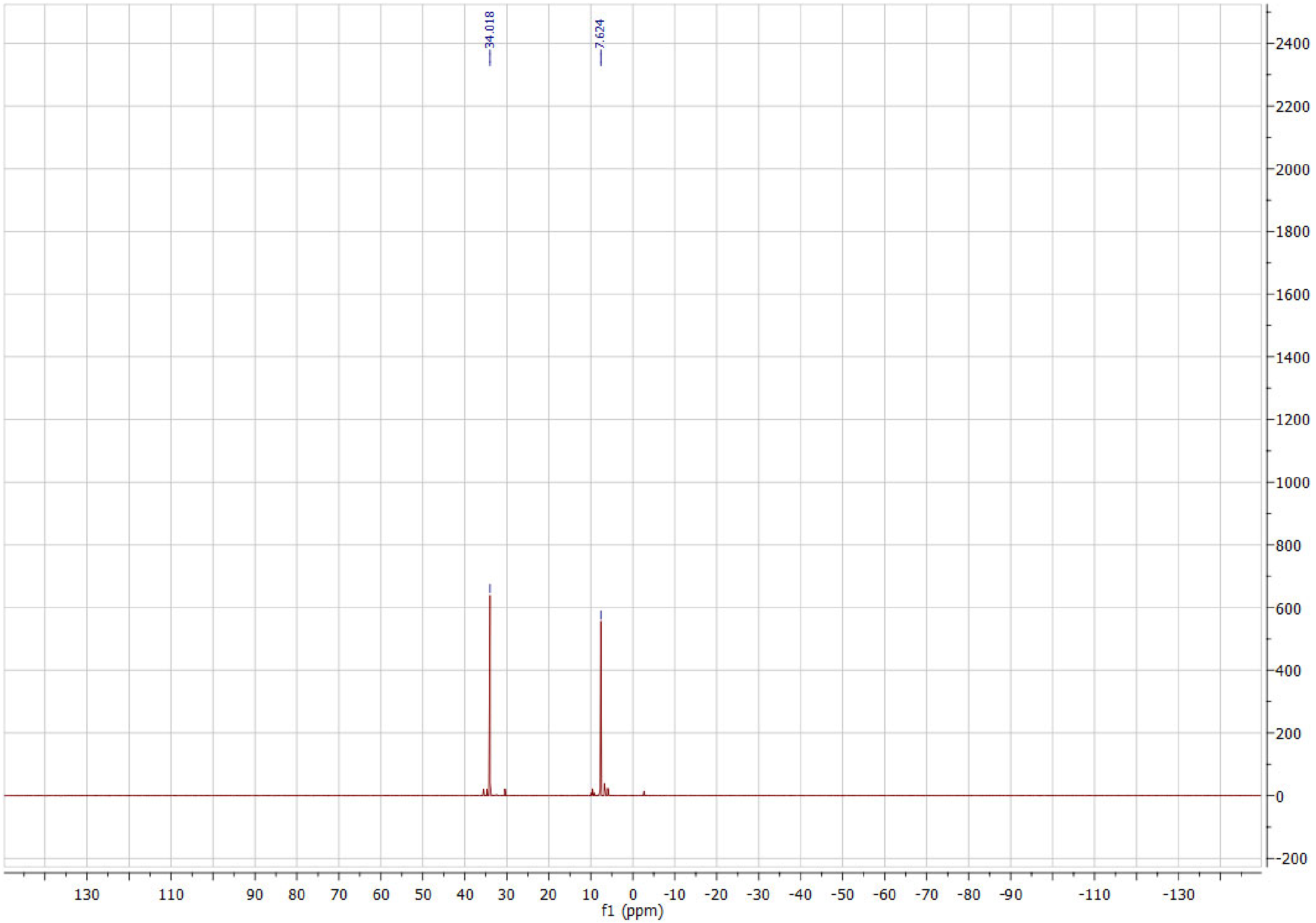

Figure S27. $^{31}P\{^{1}H\}$ NMR spectrum of **7** in C_6D_6.

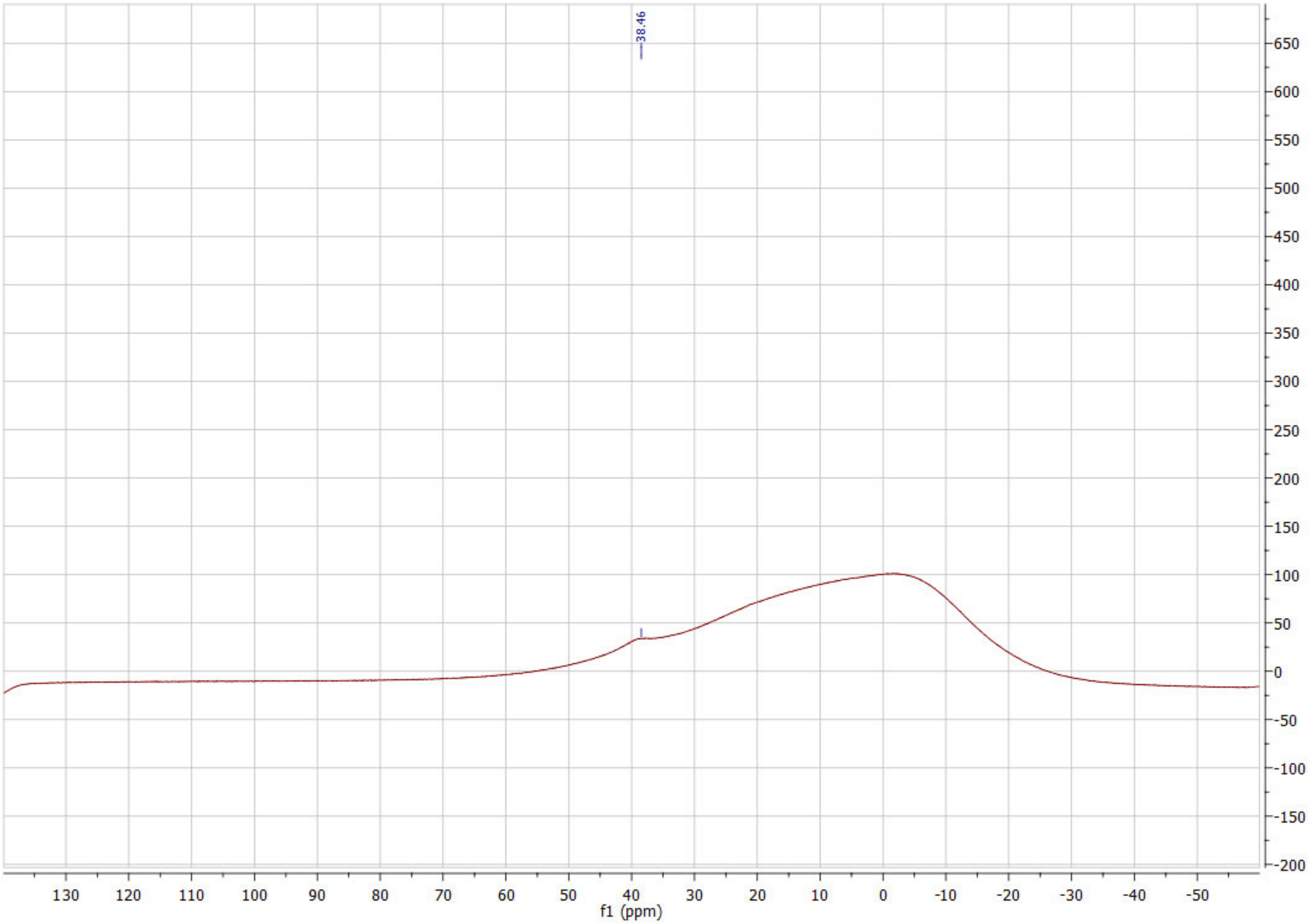

Figure S28. $^{11}B\{^{1}H\}$ NMR spectrum of **7** in C_6D_6.

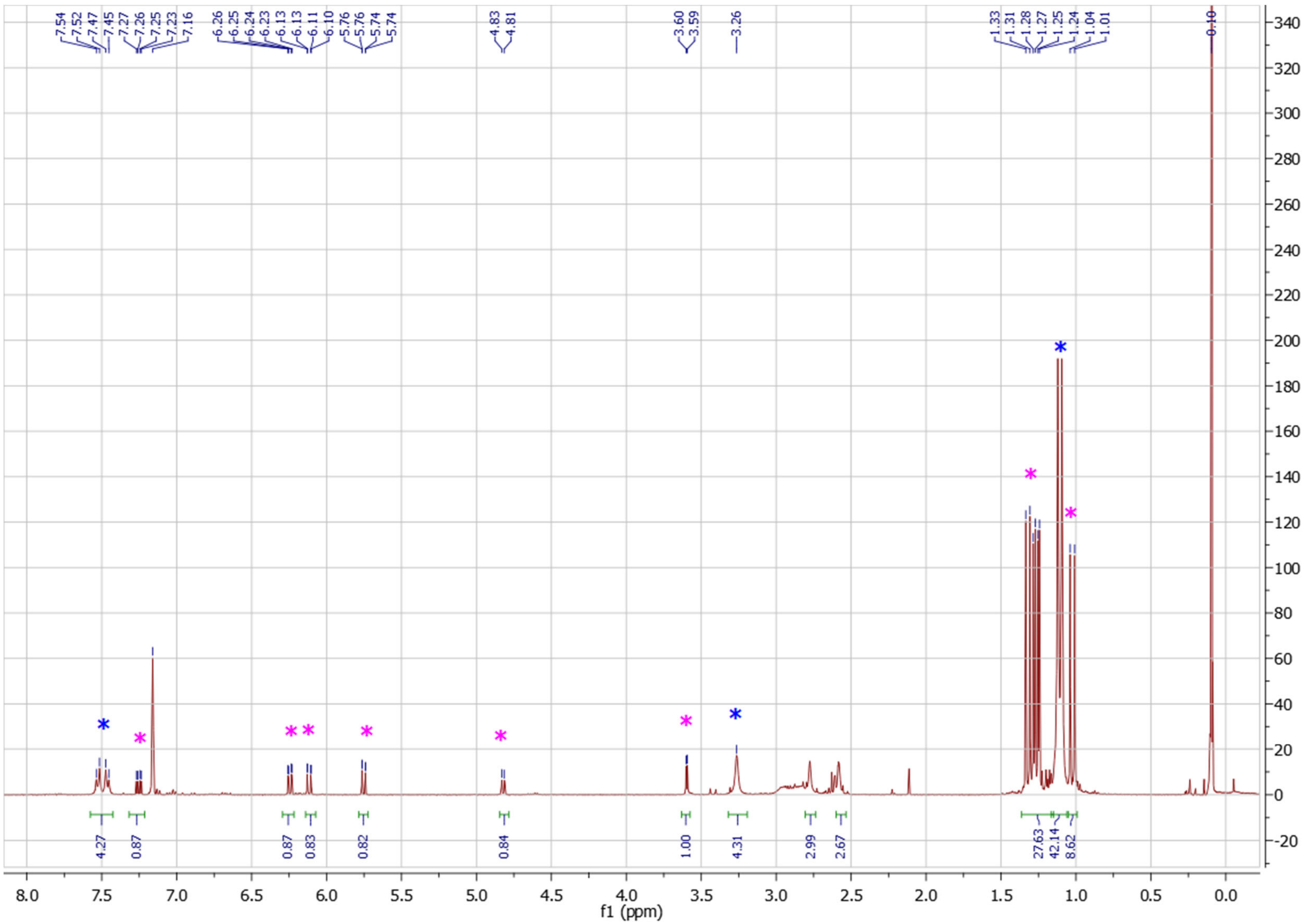

Figure S29. ^{1}H NMR spectrum of **8** in C_6D_6 (Note: the blue stars correspond to free ligand **1**, the pink stars to $L_2B_2N_2$ **8**).

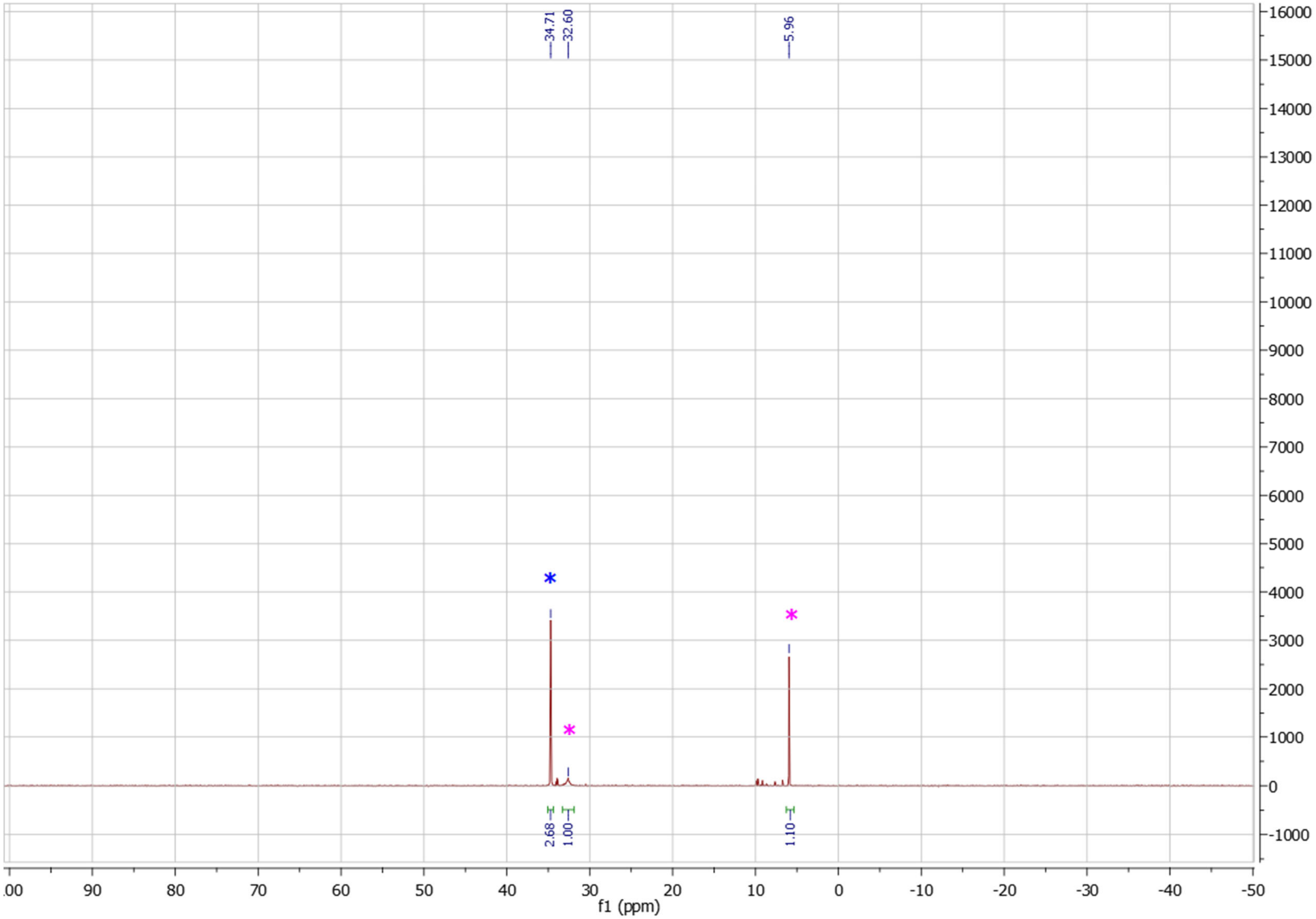

Figure S30. ^{31}P NMR spectrum of **8** in C_6D_6. (Note: the blue stars correspond to free ligand **1** while the pink ones to $L_2B_2N_2$ **8**.)

X-ray crystallographic data

The crystal data of **4b** were collected on a Bruker X8-APEX II diffractometer with a CCD area detector and multi-layer mirror monochromated $Mo_{K\alpha}$ radiation. The crystal data of **2**, **5-18C6** were collected on a Bruker D8 Quest diffractometer with a CMOS area detector and multi-layer mirror monochromated $Mo_{K\alpha}$ radiation. The crystal data of **6**, **8** were collected on a κ-Helios diffractometer with a APEX II CCD detector and Helios multilayeroptics (mirrors) $Mo_{K\alpha}$ radiation. The structures were solved using the intrinsic phasing method,[4] refined with the ShelXL program[5] and expanded using Fourier techniques. All non-hydrogen atoms were refined anisotropically. Hydrogen atoms were included in structure factor calculations.

Deposition Numbers 2099390 (for **2**), 2099391 (for **4b**), 2099392 (for **5-18-C-6**), 2099393 (for **6b**), 2099394 (for **8**) contain the supplementary crystallographic data for this paper. These data are provided free of charge by the joint Cambridge Crystallographic Data Centre and Fachinformationszentrum Karlsruhe.

Crystal data for **2**: $C_{29}H_{47}B_2Br_4Cl_9N_2P_2$, M_r = 1145.93, yellow block, 0.352×0.157×0.11 mm^3, monoclinic space group *C*2/*c*, *a* = 38.284(19) Å, *b* = 8.394(3) Å, *c* = 31.084(10) Å, β = 112.915(13)°, *V* = 9200(6) Å^3, *Z* = 8, ρ_{calcd} = 1.655 g·cm^{-3}, μ = 4.116 mm^{-1}, *F*(000) = 4544, *T* = 100(2) K, R_1 = 0.0528, wR^2 = 0.0752, 9084 independent reflections [2θ ≤ 52.044°] and 522 parameters.

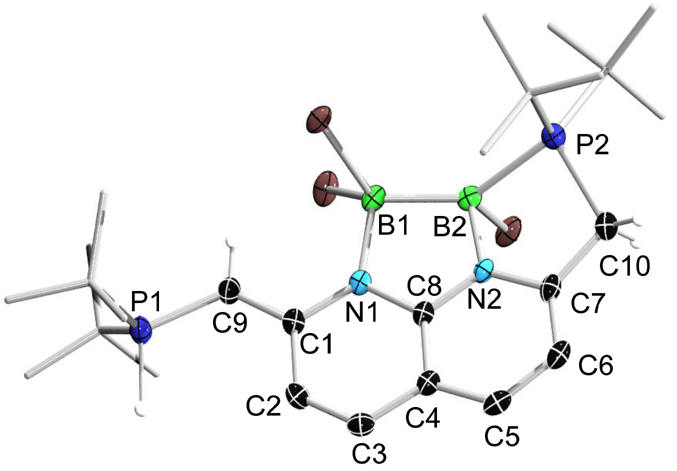

Figure S31. Crystallographically-derived solid-state structure of the cation of **2**. Atomic displacement ellipsoids drawn at 50% probability. Ellipsoids on ligand periphery and hydrogen atoms (except H bound to C9, C10) are omitted for clarity.

Note: The coordinates of H15_1 were refined freely. The chloroform molecules showed disorder. The atomic displacement parameters (ADPs) of overlapping atoms from different PARTs (C1, Cl2, Cl3, Cl4 of RESIs 8/18 and 9/19) were restrained using similarity restraint (SIMU) and rigid body restraint (RIGU). The C1-Cl2, C1-Cl3, C1-Cl4 distances as well as the Cl2-Cl3, Cl3-Cl4, Cl4-Cl1 distances of RESIs 8/18 and 9/19 were restrained using same distance restraint (SADI).

Crystal data for **4b**: $C_{32}H_{48}BBr_3F_2N_2P_2$, M_r = 811.20, yellow plate, 0.474×0.157×0.086 mm^3, triclinic space group *P-1*, *a* = 8.2639(11) Å, *b* = 14.807(7) Å, *c* = 15.3084(16) Å, α = 101.303(17) °, β = 95.287(7)°, γ = 101.638(19) °, *V* = 1782.1(9) Å^3, *Z* = 2, ρ_{calcd} = 1.512 g·cm^{-3}, μ = 3.520 mm^{-1}, *F*(000) = 824, *T* = 99(2) K, R_1 = 0.0616, wR^2 = 0.1143, 7331 independent reflections [2θ ≤ 52.880°] and 695 parameters.

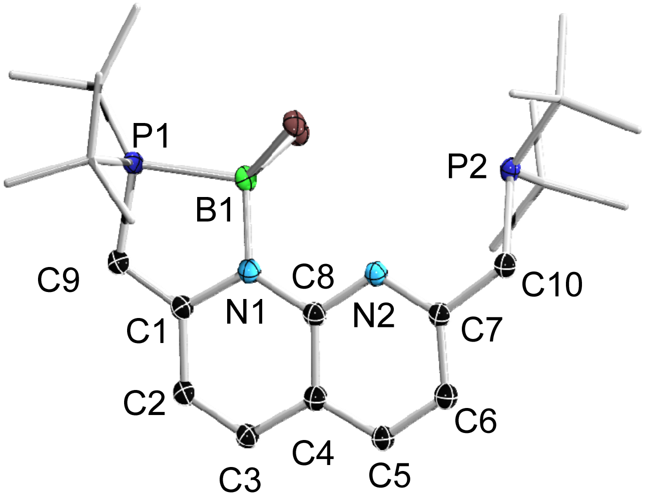

Figure S32. Crystallographically-derived solid-state structure of the cation of **4b**. Atomic displacement ellipsoids drawn at 50% probability. Ellipsoids on ligand periphery and hydrogen atoms are omitted for clarity.

Note: The whole molecule showed severe rotational disorder. The atomic displacement parameters (ADPs) of overlapping atoms from different PARTs were restrained using similarity restraint (SIMU) and rigid body restraint (RIGU). All corresponding bonds of the two PARTs were restrained using same distance restraint (SADI). All hydrogen atoms except H1_1 and H1_11 were assigned to idealized positions. The coordinates of H1_1 and H1_11 were refined freely, but restrained to a P-H bond length of 1.30 Å using the DFIX command.

Crystal data for **5-18C6**: $C_{48}H_{87}KN_2O_{10}P_2$, M_r = 953.23, red plate, 0.470×0.344×0.214 mm^3, monoclinic space group $P2_1/c$, a = 17.786(11) Å, b = 26.688(14) Å, c = 11.340(7) Å, β = 95.825(16)°, V = 5355(5) Å^3, Z = 4, ρ_{calcd} = 1.182 g·cm^{-3}, μ = 0.212 mm^{-1}, F(000) = 2072, T = 100(2) K, R_1 = 0.0980, wR^2 = 0.1742, 10533 independent reflections [2θ≤52.044°] and 690 parameters.

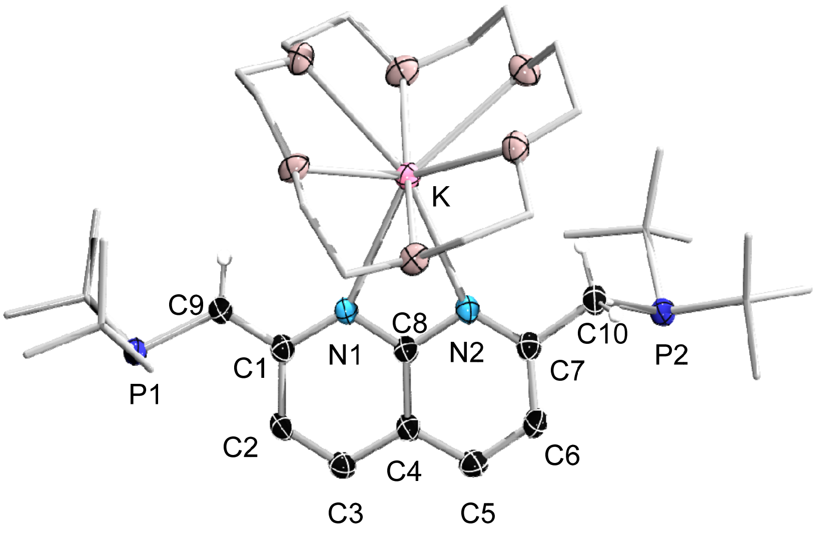

Figure S33. Crystallographically-derived solid-state structure of the cation of **5-18C6**. Atomic displacement ellipsoids drawn at 50% probability. Ellipsoids on ligand periphery and hydrogen atoms (except H bound to C9/C10) are omitted for clarity.

Note: The THF and the crown ether showed disorder. The atomic displacement parameters (ADPs) of overlapping atoms from different PARTs (O1, C2, C3, C4, C5 of RESIs 8/18; C1, O2, C3, C4, O5, C6, C7, C8, C9 of RESIs 7/17) were restrained using similarity restaint (SIMU) and rigid body restraint (RIGU). The C9-C1, C3-C4, C6-C7 distances as well as the C1-O2, O2-C3, C4-O5, O5-C6, C7-O8, O8-C9 distances of the (symmetry-generated) crown ether moieties (RESIs 7/17) were restrained using same distance restraint (SADI).

Crystal data for **6**: $C_{55}H_{87}K_4N_4P_4$, M_r = 1084.56, red plate, 0.026×0.084×0.145 mm^3, monoclinic, space group C2/c, a = 25.930(12) Å, b = 18.949(4) Å, c = 24.858(14) Å, α = 90°, β = 101.17(3) °, γ = 90 °, V = 11982(9) Å^3, Z = 8, ρ_{calcd} = 1.202 g·cm^{-3}, μ = 0.441 mm^{-1}, F(000) = 4648, T = 101(2) K, R_1 = 0.0815, wR^2 = 0.0969, 10982 independent reflections [2θ ≤ 50.750°] and 645 parameters.

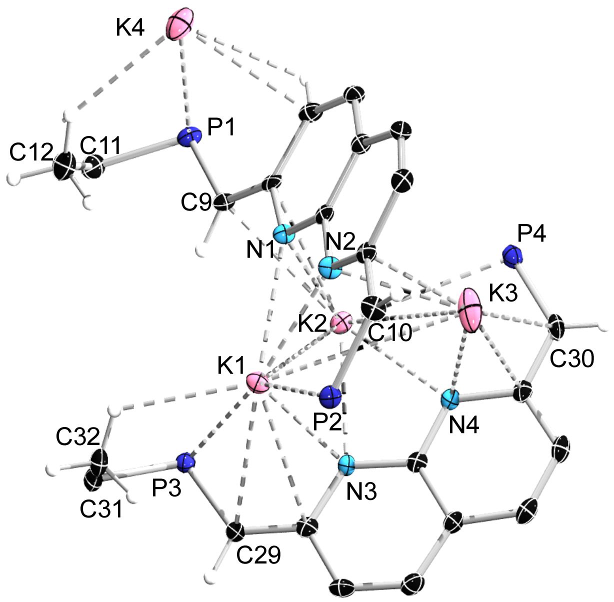

Figure S34. Crystallographically-derived solid-state structure of the cation of **6**. Atomic displacement ellipsoids drawn at 50% probability. Ellipsoids on ligand periphery and hydrogen atoms (except H bound to C9, C10, C29, C30, C12, C32) are omitted for clarity.

Note: Three reflections are omitted because they are affected by the beamstop. The hkl are (0 0 2) (1 1 1) and (-2 0 2).

Crystal data for **8**: $C_{56}H_{98}B_2N_6P_4$, M_r = 1000.90, yellow block, 0.719×0.299×0.227 mm^3, monoclinic space group *C*2/*c*, *a* = 27.958(11) Å, *b* = 21.987(11) Å, *c* = 10.318(5) Å, α = 90°, β = 99.77(2)°, γ = 90°, *V* = 6250(5)$Å^3$, *Z* = 8, ρ_{calcd} = 1.064 g·cm^{-3}, μ = 0.158 mm^{-1}, *F*(000) = 2184, *T* = 100(2) K, R_1 = 0.0819, wR^2 = 0.1858, 6828 independent reflections [2θ≤53.484°] and 322 parameters.

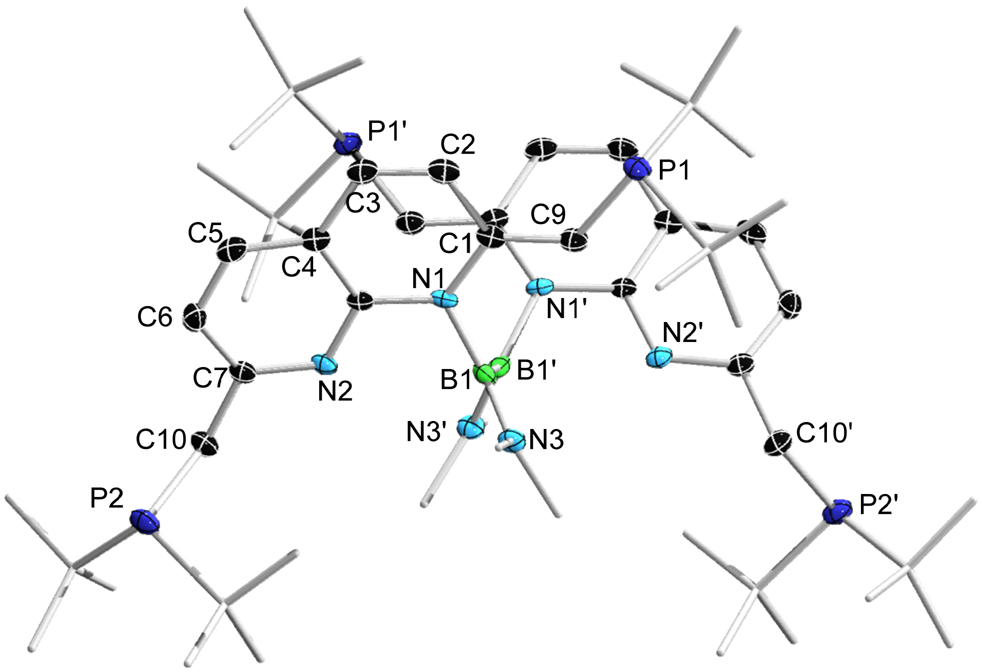

Figure S35. Crystallographically-derived solid-state structure of the cation of **8**. Atomic displacement ellipsoids drawn at 50% probability. Ellipsoids on ligand periphery and hydrogen atoms (except H bound to C9, C10, C29, C30, C12, C32) are omitted for clarity.

Note: The crystal was twinned by rotation around the [-0.5 1 1] real axis by 180.0º and was refined as a two-component twin with a final BASF of 0.28552. Two reflections are omitted because they are affected by the beam stop. The hkl are (2 0 0) (1 1 0).

HRMS spectra of compounds

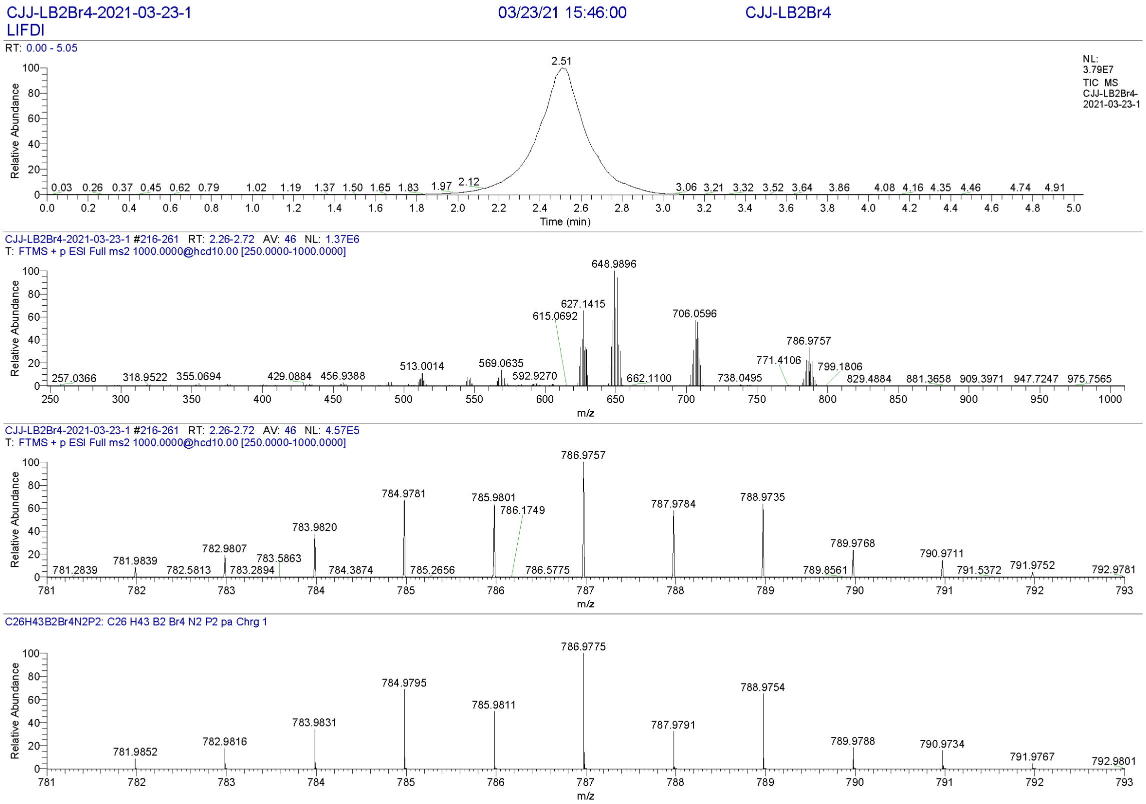

Figure S36. Fragmentation of **2** by eliminating a hydride.

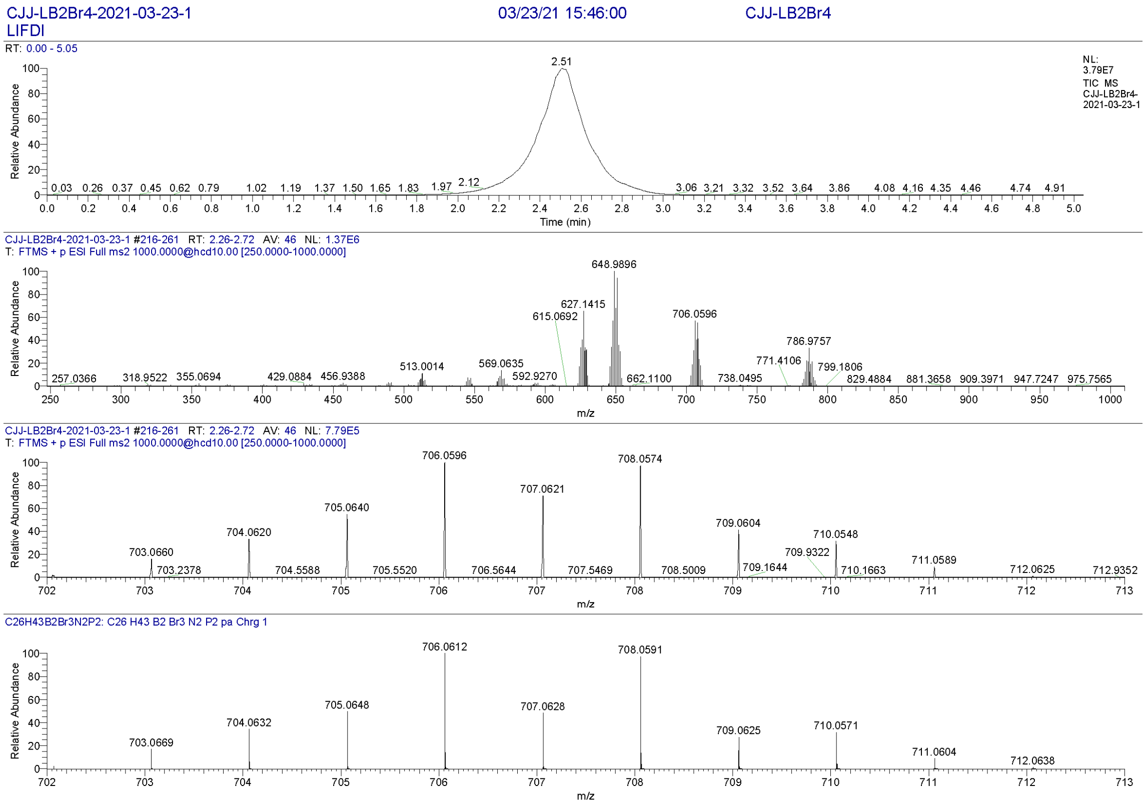

Figure S37. Fragmentation of **2** by eliminating HBr and a single electron.

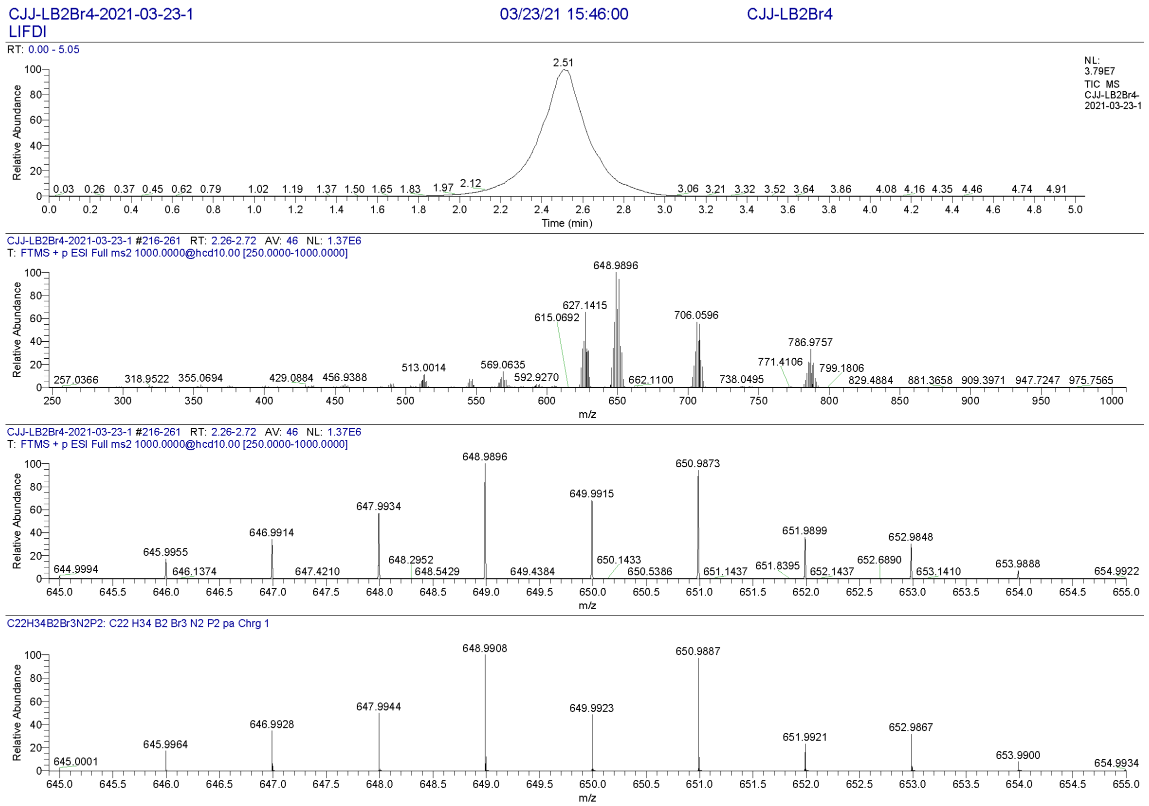

Figure S38. Further fragmentation from figure S37 by eliminating a neutal *t*Bu group.

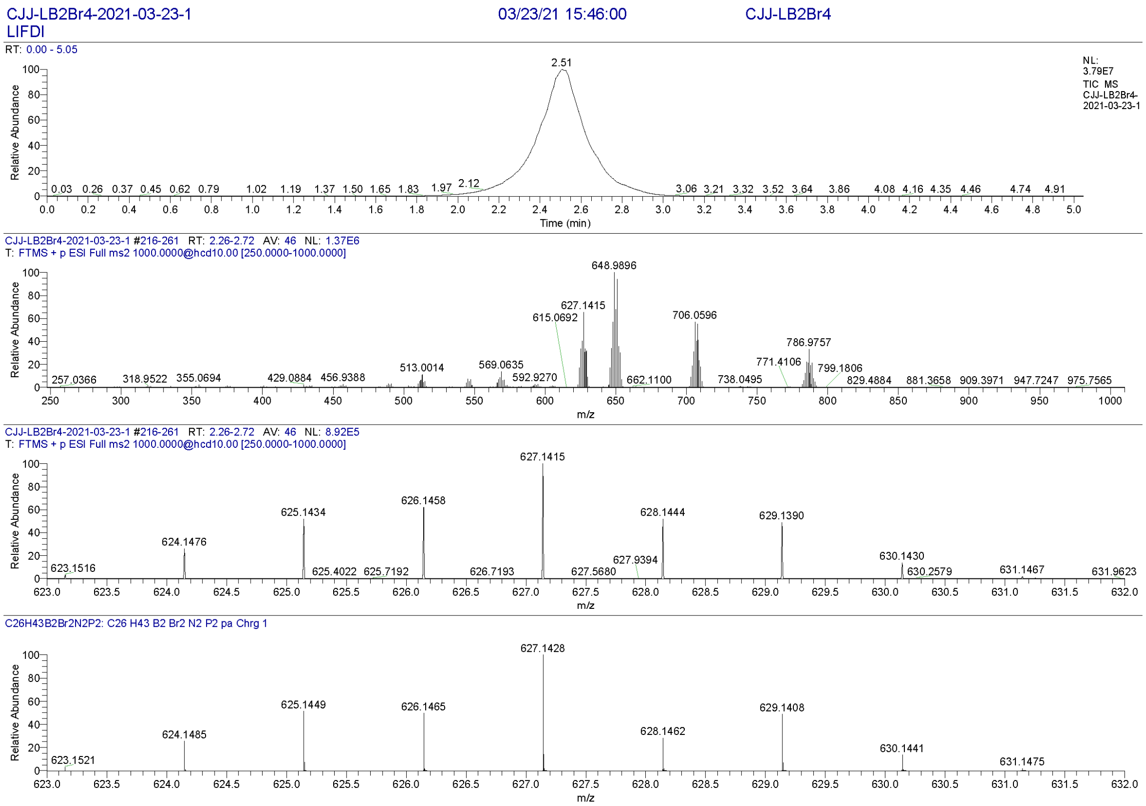

Figure S39. Further fragmentation from figure S37 by eliminating a bromine radical.

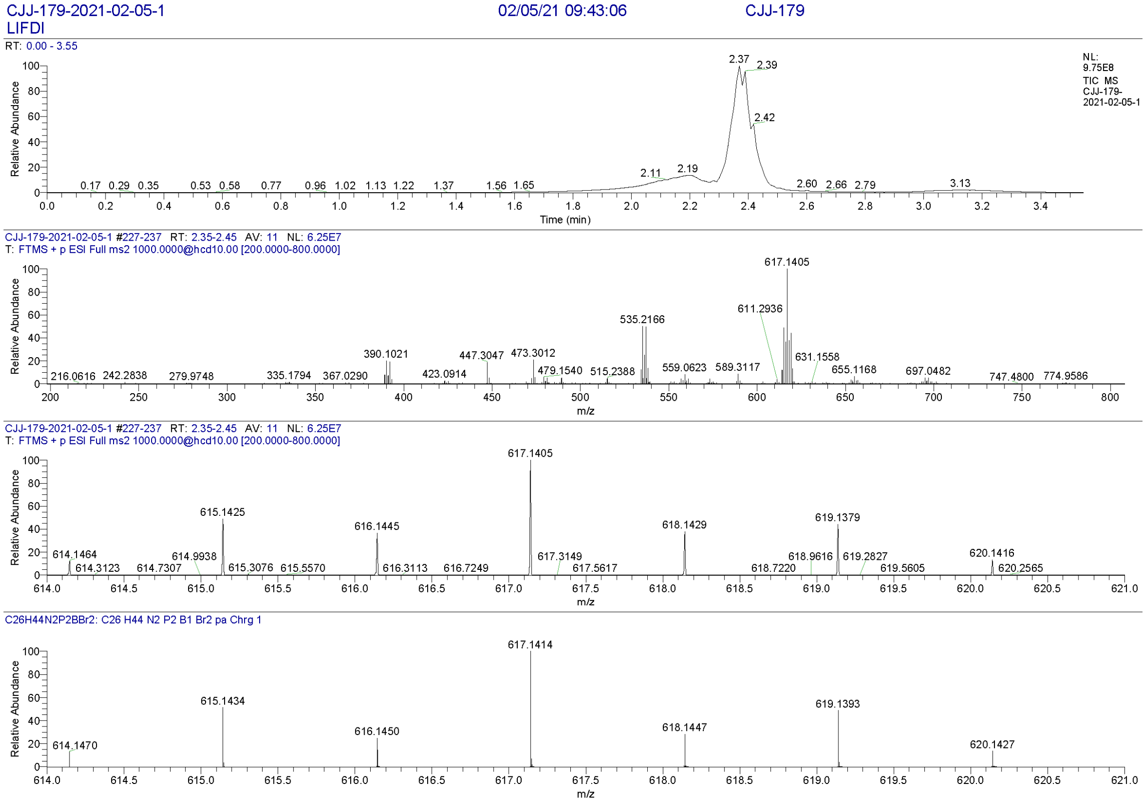

Figure S40. HRMS of compound **4b**.

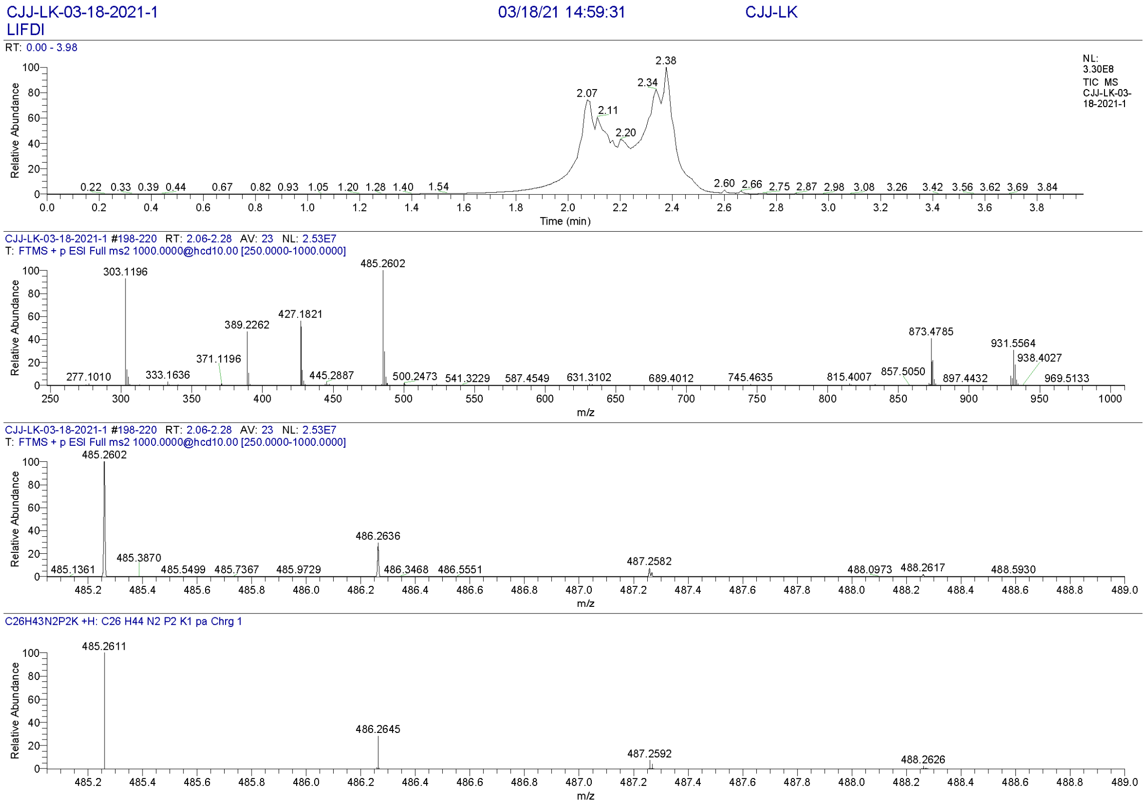

Figure S41. The $[M+H]^+$ peak for compound **5-18-C-6.**

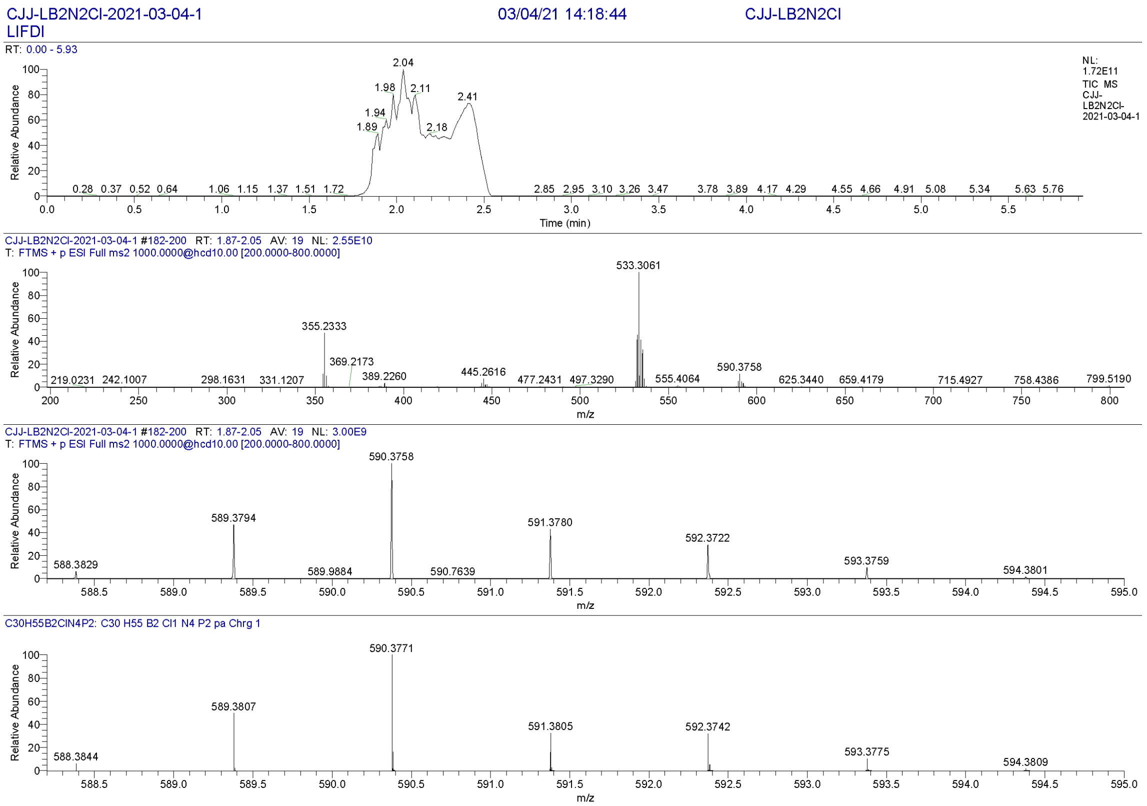

Figure S42. The molecular ion peak of compound **7**.

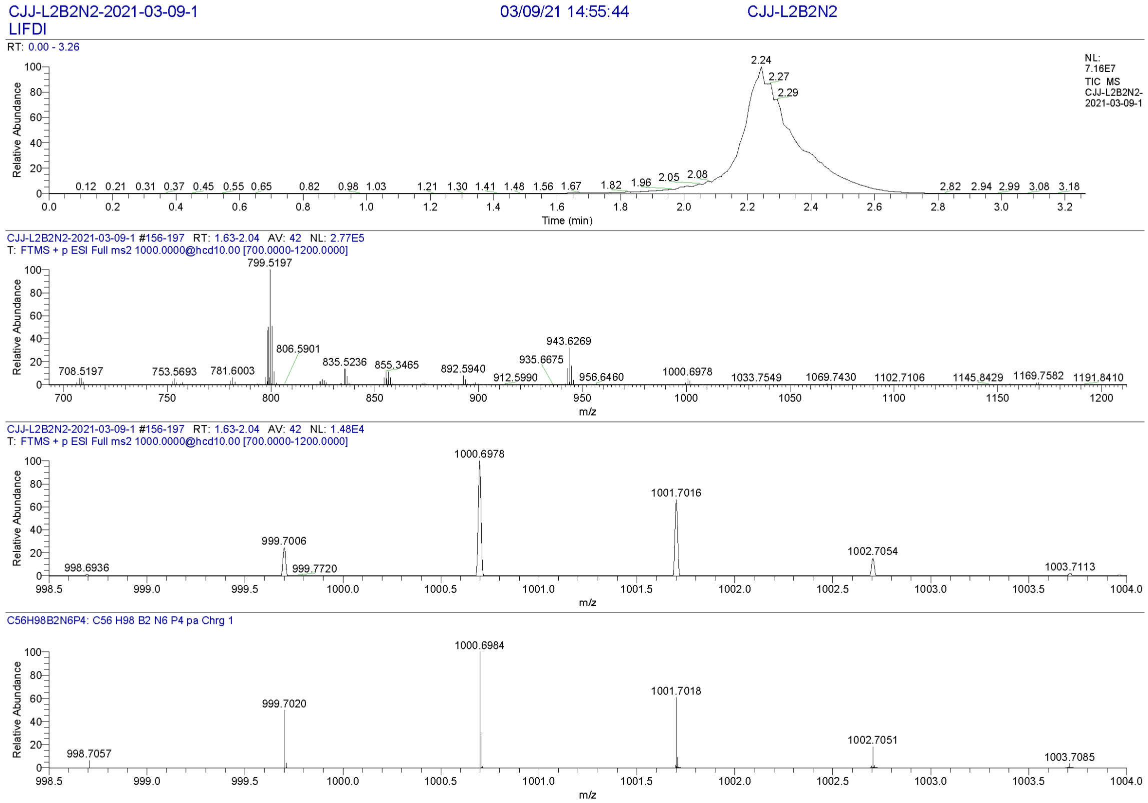

Figure S43. The molecular ion peak of compound **8**.

Computational details

Geometry optimizations and Hessian calculations were performed for **2** and **2a** at the density functional theory level. The PBE0[6] functional was employed in conjunction with the 6-31+G**[7] basis set for light elements, while Br was described with the LanL2DZ[8] basis set and effective core potential. Dispersion corrections were considered using Grimme's D3[9] model with the Becke-Johnson (BJ)[10] damping function. All optimized structures were characterized as minimum energy structures by the analysis of the computed vibrational frequencies, as in all cases only positive eigenvalues were found. The canonical Kohn-Sham molecular orbitals of the systems, their corresponding HOMO-LUMO gaps, and their electronic energies corrected by the zero-point energy (ZPE) were obtained at their corresponding optimized structures at the PBE0-D3(BJ)/6-31+G**/LanL2DZ(Br) level. Additionally, free energy calculations were conducted for investigating the relative stability of **2** and **2a** in chloroform ($CHCl_3$) medium. Solvation effects were considered using the solvation model based on density (SMD)[11] implicit solvation model with $CHCl_3$ (ε = 4.7113) as the solvent. The free energies were obtained from single-point calculations at the SMD($CHCl_3$)/PBE0-D3(BJ)/6-311++G**/LanL2DZ(Br) level. All calculations were performed using Gaussian 16, revision C.01.[12]

Cartesian coordinates

Compound **2**

N	0.526085000	0.545749000	-0.821997000
C	1.876063000	0.830519000	-0.998237000
C	2.212696000	1.844590000	-1.981721000
H	3.263064000	2.045580000	-2.156818000
C	1.283398000	2.563619000	-2.651117000
H	1.577442000	3.329020000	-3.362776000
C	-0.109524000	2.312771000	-2.410783000
C	-1.196773000	2.986200000	-2.967627000
H	-1.013422000	3.795198000	-3.669471000
C	-2.513023000	2.653066000	-2.625716000
H	-3.352648000	3.196212000	-3.043233000
C	-2.724676000	1.606478000	-1.744083000
N	-1.664527000	0.948900000	-1.236455000
C	-0.388315000	1.274683000	-1.501058000
C	2.801721000	0.173653000	-0.235694000
H	2.445212000	-0.550343000	0.490111000
P	4.544084000	0.257473000	-0.268198000
C	-4.030808000	1.067610000	-1.231332000
H	-4.418011000	0.324384000	-1.938349000
H	-4.780107000	1.853118000	-1.112688000
P	-3.608610000	0.170903000	0.348321000
H	4.985028000	0.942233000	-1.417008000
B	-1.801346000	-0.316696000	-0.355878000
B	-0.164534000	-0.422471000	0.234348000
Br	0.291546000	0.417754000	2.100399000
Br	0.633935000	-2.313736000	0.224830000
Br	-2.128464000	-1.794969000	-1.811939000
C	5.140768000	1.288745000	1.156298000
C	6.659985000	1.223117000	1.312002000
H	6.962715000	1.956762000	2.066876000
H	7.189562000	1.469527000	0.385596000

H	6.994921000	0.242796000	1.660374000
C	4.431904000	0.834724000	2.437419000
H	4.736409000	1.501002000	3.251720000
H	4.703249000	-0.183193000	2.727925000
H	3.342859000	0.892252000	2.346049000
C	4.702858000	2.718633000	0.807651000
H	3.620347000	2.787491000	0.659877000
H	5.212679000	3.102021000	-0.083152000
H	4.963408000	3.375557000	1.644346000
C	5.215533000	-1.460121000	-0.479907000
C	4.401405000	-2.078528000	-1.626200000
H	3.346287000	-2.196760000	-1.365799000
H	4.814910000	-3.069929000	-1.841109000
H	4.469639000	-1.487638000	-2.546678000
C	5.016051000	-2.272225000	0.802974000
H	5.635131000	-1.904578000	1.625933000
H	5.315314000	-3.306862000	0.604608000
H	3.969417000	-2.294928000	1.123226000
C	6.694643000	-1.400072000	-0.875080000
H	7.331802000	-0.998427000	-0.085457000
H	6.852781000	-0.812637000	-1.785846000
H	7.034938000	-2.419916000	-1.083366000
C	-3.614513000	1.502329000	1.678501000
C	-2.573450000	2.566080000	1.298473000
H	-2.541923000	3.302987000	2.108582000
H	-2.845129000	3.104224000	0.384831000
H	-1.567407000	2.156695000	1.199536000
C	-3.229056000	0.867194000	3.019864000
H	-3.132869000	1.664934000	3.764753000
H	-2.266963000	0.353034000	2.966607000
H	-3.985218000	0.170493000	3.386963000
C	-4.971177000	2.210158000	1.789183000
H	-5.764811000	1.572766000	2.174050000
H	-5.300182000	2.644404000	0.839904000

H	-4.852986000	3.042027000	2.492449000
C	-4.796641000	-1.252594000	0.690661000
C	-5.963899000	-0.874606000	1.608632000
H	-6.572188000	-1.775605000	1.742897000
H	-6.611242000	-0.113009000	1.165890000
H	-5.652572000	-0.549973000	2.602089000
C	-3.946079000	-2.343085000	1.361626000
H	-3.500561000	-1.996620000	2.299232000
H	-3.145387000	-2.700832000	0.709977000
H	-4.595075000	-3.193361000	1.597805000
C	-5.415085000	-1.765934000	-0.616948000
H	-6.007451000	-2.655060000	-0.374787000
H	-4.673710000	-2.055258000	-1.360669000
H	-6.100524000	-1.030895000	-1.051877000

Compound **2a**

N	-1.662788000	1.252535000	-0.886705000
C	-2.771813000	1.857099000	-1.463970000
C	-2.487700000	2.971809000	-2.340969000
H	-3.321414000	3.503322000	-2.787001000
C	-1.209193000	3.338198000	-2.608944000
H	-1.008067000	4.171823000	-3.275344000
C	-0.084640000	2.636660000	-2.028991000
C	1.259518000	2.889770000	-2.222038000
H	1.577175000	3.697354000	-2.875307000
C	2.219161000	2.097595000	-1.568801000
H	3.269069000	2.303016000	-1.732571000
C	1.838233000	1.067532000	-0.729560000
N	0.518725000	0.815403000	-0.501363000
C	-0.406804000	1.575796000	-1.149614000
C	-3.961270000	1.277926000	-1.132057000
H	-4.913957000	1.639487000	-1.496921000

P	-3.704440000	-0.236649000	-0.182586000
C	2.768851000	0.125040000	-0.034264000
H	2.392203000	-0.889204000	-0.223191000
H	2.672288000	0.240198000	1.054143000
P	4.536954000	0.080886000	-0.407821000
H	4.775276000	0.693210000	-1.652066000
B	-0.219125000	-0.380940000	0.318594000
B	-1.828294000	0.287002000	0.285617000
Br	0.242288000	-2.090087000	-0.830002000
Br	-1.946361000	1.646382000	1.912004000
Br	0.576254000	-0.721325000	2.176946000
C	-4.914253000	-0.428579000	1.248275000
C	-4.171882000	-1.162489000	2.374562000
H	-4.862378000	-1.296234000	3.214881000
H	-3.307514000	-0.601950000	2.735272000
H	-3.836704000	-2.156232000	2.061634000
C	-6.167124000	-1.226652000	0.876376000
H	-6.816646000	-1.240576000	1.758624000
H	-5.952376000	-2.265635000	0.619328000
H	-6.733515000	-0.763727000	0.064049000
C	-5.379166000	0.962678000	1.698873000
H	-5.992021000	1.443271000	0.930127000
H	-4.554386000	1.626989000	1.952744000
H	-6.003069000	0.835954000	2.591047000
C	-3.815962000	-1.623887000	-1.455090000
C	-2.811085000	-1.291490000	-2.566930000
H	-3.119246000	-0.403357000	-3.125741000
H	-2.787636000	-2.135454000	-3.265442000
H	-1.795303000	-1.151330000	-2.199948000
C	-5.196057000	-1.700457000	-2.120181000
H	-5.967138000	-2.122179000	-1.478423000

H	-5.109575000	-2.352374000	-2.996936000
H	-5.530828000	-0.721587000	-2.479654000
C	-3.449023000	-2.949713000	-0.783747000
H	-4.178942000	-3.246813000	-0.026104000
H	-2.458296000	-2.913246000	-0.322871000
H	-3.428925000	-3.738166000	-1.544743000
C	5.474957000	1.091939000	0.828683000
C	4.722967000	2.422990000	0.975762000
H	5.269230000	3.039866000	1.697029000
H	3.705564000	2.300181000	1.357063000
H	4.682060000	2.983988000	0.037087000
C	6.888961000	1.364993000	0.302742000
H	7.397573000	2.024162000	1.013783000
H	6.875997000	1.877700000	-0.664713000
H	7.490293000	0.459175000	0.209831000
C	5.518114000	0.360109000	2.174234000
H	6.127030000	-0.546480000	2.130500000
H	4.522254000	0.098170000	2.546747000
H	5.975256000	1.026101000	2.913210000
C	4.972455000	-1.702857000	-0.664662000
C	4.448000000	-2.560878000	0.494203000
H	4.701711000	-3.603458000	0.276175000
H	3.360981000	-2.514654000	0.607724000
H	4.910204000	-2.304964000	1.450043000
C	6.487837000	-1.862286000	-0.811798000
H	7.015364000	-1.682609000	0.128479000
H	6.907240000	-1.211812000	-1.586412000
H	6.694652000	-2.895955000	-1.107404000
C	4.272380000	-2.099613000	-1.975140000
H	4.478585000	-3.159180000	-2.159397000
H	4.652826000	-1.536191000	-2.833391000

H	3.183836000	-1.991424000	-1.929309000

References

[1] a) E. Kounalis, M. Lutz, D. L. J. Broere, *Chem. Eur. J.* **2019**, *25*, 13280-13284; b) E. Kounalis, M. Lutz, D. L. J. Broere, *Organometallics* **2020**, *39*, 585-592.

[2] M. Arrowsmith, J. Böhnke, H. Braunschweig, A. Deißenberger, R. D. Dewhurst, W. C. Ewing, C. Hörl, J. Mies, J. H. Muessig, *Chem. Commun.* **2017**, *53*, 8265-8267.

[3] S. Kundu, S. Sinhababu, M. M. Siddiqui, A. V. Luebben, B. Dittrich, T. Yang, G. Frenking, H. W. Roesky, *J. Am. Chem. Soc.* **2018**, *140*, 9409-9412.

[4] G. Sheldrick, *Acta Cryst.* **2015**, *A71*, 3–8.

[5] G. Sheldrick, *Acta Cryst*. **2008**, *A64*, 112–122.

[6] a) M. Ernzerhof, G. E. Scuseria, *J. Chem. Phys.* **1999**, *110*, 5029–5036; b) C. Adamo, V. Barone, *J. Chem. Phys.* **1999**, *110*, 6158–6170.

[7] a) R. Ditchfield, W. J. Hehre, J. A. Pople, *J. Chem. Phys.* **1971**, *54*, 724–728; b) W. J. Hehre, R. Ditchfield, J. A. Pople, *J. Chem. Phys.* **1972**, *56*, 2257–2261; c) P. C. Hariharan, J. A. Pople, *Theor. Chim. Acta* **1973**, *28*, 213–222; d) J. D. Dill, J. A. Pople, *J. Chem. Phys.* **1975**, *62*, 2921–2923; e) M. S. Gordon, J. S. Binkley, J. A. Pople, W. J. Pietro, W. J. Hehre, *J. Am. Chem. Soc.* **1982**, *104*, 2797–2803; f) M. M. Francl, W. J. Pietro, W. J. Hehre, J. S. Binkley, M. S. Gordon, D. J. DeFrees, J. A. Pople, *J. Chem. Phys.* **1982**, *77*, 3654–3665; g) T. Clark, J. Chandrasekhar, G. W. Spitznagel, P. V. R. Schleyer, *J. Comput. Chem.* **1983**, *4*, 294–301; h) G. W. Spitznagel, T. Clark, P. von Ragué Schleyer, W. J. Hehre, *J. Comput. Chem.* **1987**, *8*, 1109–1116.

[8] W. R. Wadt, P. J. Hay, *J. Chem. Phys.* **1985**, *82*, 284–298.

[9] S. Grimme, J. Antony, S. Ehrlich, H. Krieg, *J. Chem. Phys.* **2010**, *132*, 154104.

[10] S. Grimme, S. Ehrlich, L. Goerigk, *J. Comput. Chem.* **2011**, *32*, 1456–1465.

[11] A. V. Marenich, C. J. Cramer, D. G. Truhlar, *J. Phys. Chem. B* **2009**, *113*, 6378–6396.

[12] M. J. Frisch, G. W. Trucks, H. B. Schlegel, G. E. Scuseria, M. A. Robb, J. R. Cheeseman, G. Scalmani, V. Barone, B. Mennucci, G. A. Petersson, H. Nakatsuji, M. Caricato, X. Li, H. P. Hratchian, A. F. Izmaylov, J. Bloino, G. Zheng, J. L. Sonnenberg, M. Hada, M. Ehara, K. Toyota, R. Fukuda, J. Hasegawa, M. Ishida, T. Nakajima, Y. Honda, O. Kitao, H. Nakai, T. Vreven, J. A. Montgomery Jr., J. E. Peralta, F. Ogliaro, M. Bearpark, J. J. Heyd, E. Brothers, K. N. Kudin, V. N. Staroverov, R. Kobayashi, J. Normand, K. Raghavachari, A. Rendell, J. C. Burant, S. S. Iyengar, J. Tomasi, M. Cossi, N. Rega, J. M. Millam, M. Klene, J. E. Knox, J. B. Cross, V. Bakken, C. Adamo, J. Jaramillo, R. Gomperts, R. E. Stratmann, O. Yazyev, A. J. Austin, R. Cammi, C. Pomelli, J. W. Ochterski, R. L. Martin, K.

Morokuma, V. G. Zakrzewski, G. A. Voth, P. Salvador, J. J. Dannenberg, S. Dapprich, A. D. Daniels, Ö. Farkas, J. B. Foresman, J. V Ortiz, J. Cioslowski, D. J. Fox, *Gaussian 16, Revision C.01*, Gaussian, Inc., Wallingford CT, **2016**.
